# Supplementary material for: Variational Mode Decomposition Analysis of Electroencephalograms during General Anesthesia: Using the Grey Wolf Optimizer to Determine Hyperparameters
Source: Sensors (Basel). 2024 Sep 4;24(17):5749. doi: 10.3390/s24175749 (PMC11398215; doi:10.3390/s24175749)
Supplement: Supplementary file 1 [file sensors-24-05749-s001.zip › Supple1_Table_S1.pdf]

Table S1. Each patient characteristics and surgical and anesthetic time.

no.	age/sex	ht/bw	disease/surgery	surgery time	anesthesia time
1	17M	150/55	patellar luxation/medial patellofemoral ligament reconstruction	1:59	3:01
2	34F	164/65	ankle instability/lateral ankle ligament suture	1:07	2:08
3	41F	153/48	ovarian endometriosis cyst/ laparoscopic ovarian tumor resection	3:07	4:11

ht: height, bw: body weight
