# Supplementary material for: Variational Mode Decomposition Analysis of Electroencephalograms during General Anesthesia: Using the Grey Wolf Optimizer to Determine Hyperparameters
Source: Sensors (Basel). 2024 Sep 4;24(17):5749. doi: 10.3390/s24175749 (PMC11398215; doi:10.3390/s24175749)

VMD_python_PRO_maintenance-GWO_seq_1024

March 17, 2024

1 # Variational Mode Decomposition & Grey Wolf Optimizer: Python 3.8 Prop#1 maintenance¶

[1]:
```
import numpy as np
import pandas as pd
import matplotlib.pyplot as plt
import matplotlib.patches as patches
import random

from scipy.fftpack import fft
from scipy import signal as sig #Signal function import from SciPy package
from scipy import ndimage
import scipy.stats as stats
from scipy.stats import entropy
from scipy.signal import hilbert

from IPython.display import set_matplotlib_formats
#set_matplotlib_formats('svg')
```

2 # Get EEG data

[2]:
```
df_tsv = pd.read_table('./PRO_2020_1_eeg_bis_EME10.tsv')
```

[3]:
```
df_tsv.head(5)
```

[3]:

	Ch	Time	ch[0]	ch[1]	ch[2]	ch[3]	ch[4]	ch[5]	ch[6]	ch[7]	\
0	ch1:	11:59:57	-4.95	-5.10	-3.30	6.80	23.15	30.25	25.45	9.30	
1	ch1:	11:59:57	7.00	-6.20	-20.75	-24.20	-7.95	12.30	31.10	44.20	
2	ch1:	11:59:57	31.85	27.70	15.05	-2.25	-20.35	-29.20	-32.30	-28.25	
3	ch1:	11:59:57	-35.70	-20.65	4.45	25.05	30.60	20.70	2.90	-16.80	
4	ch1:	11:59:57	7.65	-24.55	-43.55	-36.55	-12.50	18.40	42.95	48.25	

	ch[8]	ch[9]	ch[10]	ch[11]	ch[12]	ch[13]	ch[14]	ch[15]
0	-15.70	-32.85	-26.55	-3.15	19.40	31.20	31.55	20.45
1	36.15	14.65	0.85	-11.05	-21.05	-20.70	-7.05	16.30
2	-9.55	14.10	31.10	33.70	16.20	-10.70	-28.75	-36.70

```
3 -29.80 -31.10  -21.10   -1.95   18.40   31.05   33.95   28.80
4  34.25  14.05   -3.65  -15.05  -14.05    2.55   27.30   45.95
```

[4]:
```
df_tsv.tail(5)
```

[4]:
```
        Ch      Time    ch[0]    ch[1]    ch[2]    ch[3]    ch[4]  ch[5]    ch[6]  \
4692  ch1:  12:09:55 -401.20 -151.55 -320.65 -351.10 -302.90 -340.2 -227.50
4693  ch1:  12:09:55 -247.25 -161.90 -298.00 -357.20 -212.50 -144.9 -385.45
4694  ch1:  12:09:55 -178.85 -468.35 -421.45  -30.60 -201.95 -363.7 -338.10
4695  ch1:  12:09:55 -325.05 -366.25 -395.75 -244.95  -96.55 -295.6 -391.10
4696  ch1:  12:09:55 -310.10 -317.05  -68.65 -199.90 -444.80 -277.5 -100.25

        ch[7]    ch[8]    ch[9]   ch[10]   ch[11]   ch[12]   ch[13]   ch[14]   ch[15]
4692 -151.60 -383.70 -409.30 -206.10 -215.85 -295.50 -194.55 -331.65 -275.75
4693 -255.25 -153.15 -306.65 -410.60 -205.35 -165.50 -247.55 -452.45 -203.15
4694 -484.55 -246.20 -167.90 -383.95 -331.75 -252.20 -450.05 -237.40 -105.75
4695 -195.55 -460.50 -290.55 -115.60 -301.55 -321.55  -74.15 -316.05 -332.35
4696 -297.45 -245.70 -305.60 -244.75 -168.40 -213.35 -373.90 -209.70 -204.60
```

[5]:
```
cols = ['Time', 'eeg']
temp_data = []  # Initialization

for row in range(len(df_tsv)):
#for row in range(0, 2048):
#for row in range(0, 4682):
#for row in range(0, 4696):
    t_tmp = df_tsv.iat[row, 1]
    for column in range(2, 18):
        eeg_tmp = df_tsv.iat[row, column]
        data_tmp = [t_tmp, eeg_tmp]  # add data as list
        temp_data.append(data_tmp)

# temp_data Making DataFrame from list
df_eeg = pd.DataFrame(temp_data, columns=cols)
```

[6]:
```
df_eeg
```

[6]:
```
           Time     eeg
0      11:59:57   -4.95
1      11:59:57   -5.10
2      11:59:57   -3.30
3      11:59:57    6.80
4      11:59:57   23.15
…           …       …
75147  12:09:55 -168.40
75148  12:09:55 -213.35
75149  12:09:55 -373.90
```

```
75150  12:09:55 -209.70
75151  12:09:55 -204.60

[75152 rows x 2 columns]
```

[7]:
```
df_eeg.to_csv("data_eeg_prop_2024_1S/eeg_linealized_eeg_bis_std2.csv")
```

[8]:
```
#Place brain wave data in the data frame
time = df_eeg['Time'].values
eeg = df_eeg['eeg'].values
```

[9]:
```
# Time-series graph display of EEG data
fig, ax = plt.subplots(figsize=(18,6))
plt.plot(eeg)
plt.savefig('data_eeg_prop_2024_1S/spectral_analysis_3_fig_1.svg')
```

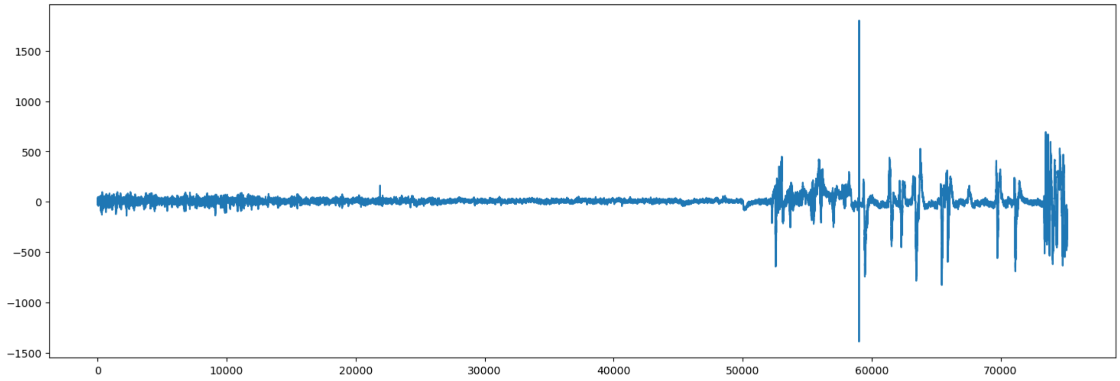

[10]:
```
eeg_ip = df_eeg['eeg'].values
```

[11]:
```
eeg_1024 = eeg_ip[0:1024]
time_1024 = time[0:1024]
```

[12]:
```
time_1024
```

[12]:
```
array(['11:59:57', '11:59:57', '11:59:57', …, '12:00:03', '12:00:03',
       '12:00:03'], dtype=object)
```

[13]:
```
fig, ax = plt.subplots(figsize=(18,6))
plt.ylim(-100, 100)
plt.plot(eeg_1024[0:1024], linewidth =0.5)
plt.savefig('data_eeg_prop_2024_1S/VMD_fig_eeg.svg')
```

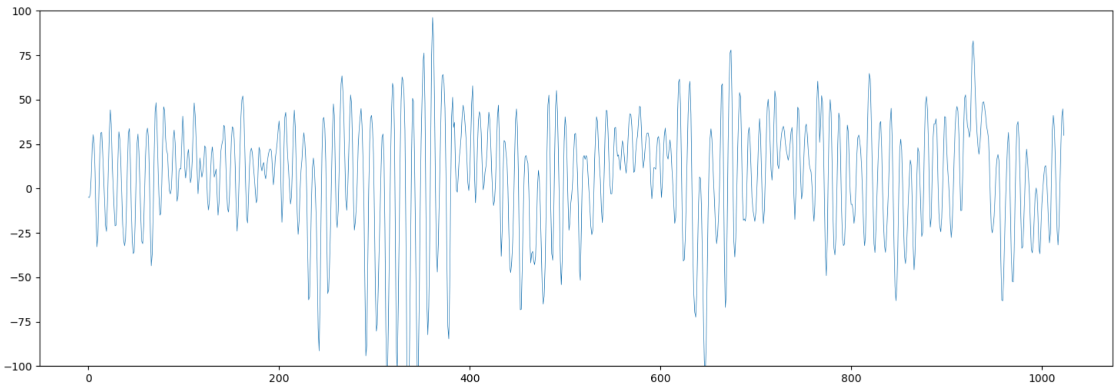

[14]:
```
N=len(eeg_1024[0:1024]) # n of data points
f_s = 128 #sampling frequency
T = 1.0/f_s  #data length
s_rate = 1/f_s #sampling length
```

[15]:
```
xfft = np.linspace(0, int(1.0/(2.0*T)), int(N/2))
yfft=fft(eeg_1024[0:1024])
```

[16]:
```
plt.plot(xfft, 2.0/N*abs(yfft[0:int(N/2)]), linewidth =0.5, ␣
↪label="periodogram")
plt.legend(bbox_to_anchor=(1, 1), loc='upper right', borderaxespad=1,␣
↪fontsize=12)
plt.grid()
plt.yscale("log")
plt.savefig('data_eeg_prop_2024_1S/eeg_fft_maintenance.svg')
```

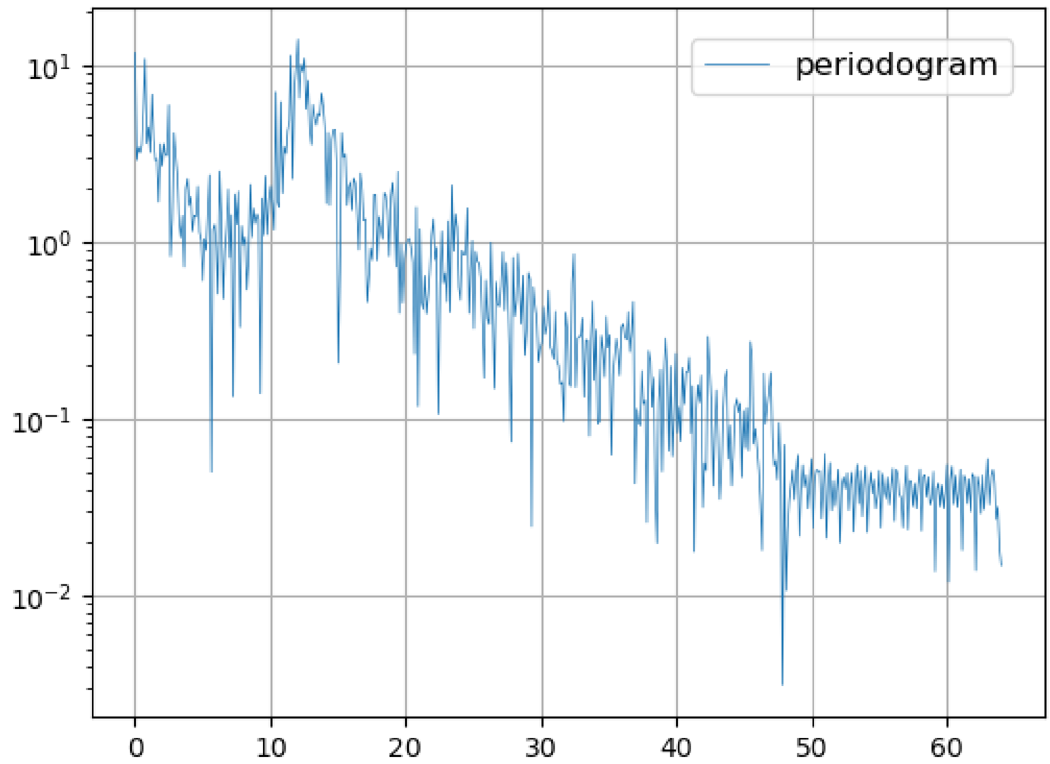

3 # Divide into 6 IMFs

[17]:
```
def  VMD(f, alpha, tau, K, DC, init, tol):
    """
    u,u_hat,omega = VMD(f, alpha, tau, K, DC, init, tol)
    Variational mode decomposition
    Python implementation by Vinícius Rezende Carvalho - vrcarva@gmail.com
    code based on Dominique Zosso's MATLAB code, available at:
    https://www.mathworks.com/matlabcentral/fileexchange/
↪44765-variational-mode-decomposition
    Original paper:
    Dragomiretskiy, K. and Zosso, D. (2014) ‘Variational Mode Decomposition’,
    IEEE Transactions on Signal Processing, 62(3), pp. 531-544. doi: 10.1109/
↪TSP.2013.2288675.


    Input and Parameters:
    ---------------------
    f       - the time domain signal (1D) to be decomposed
    alpha   - the balancing parameter of the data-fidelity constraint
    tau     - time-step of the dual ascent ( pick 0 for noise-slack )
```

```
    K       - the number of modes to be recovered
    DC      - true if the first mode is put and kept at DC (0-freq)
    init    - 0 = all omegas start at 0
                       1 = all omegas start uniformly distributed
                      2 = all omegas initialized randomly
    tol     - tolerance of convergence criterion; typically around 1e-6

    Output:
    -------
    u       - the collection of decomposed modes
    u_hat   - spectra of the modes
    omega   - estimated mode center-frequencies

    # ---------------------
    #  signal  - the time domain signal (1D) to be decomposed
    #
    #  alpha   - the balancing parameter of the data-fidelity constraint
    #
    #  tau     - time-step of the dual ascent ( pick 0 for noise-slack )
    #
    #  K       - the number of modes to be recovered
    #  DC      - true if the first mode is put and kept at DC (0-freq)
    #            DC
    #  init    - 0 = all omegas start at 0
    #                   1 = all omegas start uniformly distributed
    #                   2 = all omegas initialized randomly
    #  tol     - tolerance of convergence criterion; typically around 1e-6
    #                 1e-6
    #
    #  Output:
    #  -------
    #  u       - the collection of decomposed modes
    #  u_hat   - spectra of the modes
    #  omega   - estimated mode center-frequencies
    #
    """

    if len(f)%2:
       f = f[:-1]

    # Period and sampling frequency of input signal
    fs = 1./len(f)

    ltemp = len(f)//2
    fMirr =  np.append(np.flip(f[:ltemp],axis = 0),f)
    fMirr = np.append(fMirr,np.flip(f[-ltemp:],axis = 0))
```

```python
    # Time Domain 0 to T (of mirrored signal)
    T = len(fMirr)
    t = np.arange(1,T+1)/T

    # Spectral Domain discretization
    freqs = t-0.5-(1/T)

    # Maximum number of iterations (if not converged yet, then it won't anyway)
    Niter = 500
    # For future generalizations: individual alpha for each mode
    Alpha = alpha*np.ones(K)

    # Construct and center f_hat
    f_hat = np.fft.fftshift((np.fft.fft(fMirr)))
    f_hat_plus = np.copy(f_hat) #copy f_hat
    f_hat_plus[:T//2] = 0

    # Initialization of omega_k
    omega_plus = np.zeros([Niter, K])


    if init == 1:
        for i in range(K):
            omega_plus[0,i] = (0.5/K)*(i)
    elif init == 2:
        omega_plus[0,:] = np.sort(np.exp(np.log(fs) + (np.log(0.5)-np.
↪log(fs))*np.random.rand(1,K)))
    else:
        omega_plus[0,:] = 0

    # if DC mode imposed, set its omega to 0
    if DC:
        omega_plus[0,0] = 0

    # start with empty dual variables
    lambda_hat = np.zeros([Niter, len(freqs)], dtype = complex)

    # other inits
    uDiff = tol+np.spacing(1) # update step
    n = 0 # loop counter
    sum_uk = 0 # accumulator
    # matrix keeping track of every iterant // could be discarded for mem
    u_hat_plus = np.zeros([Niter, len(freqs), K],dtype=complex)

    #*** Main loop for iterative updates***
```

```
  while ( uDiff > tol and  n < Niter-1 ): # not converged and below␣
↪iterations limit
      # update first mode accumulator
      k = 0
      sum_uk = u_hat_plus[n,:,K-1] + sum_uk - u_hat_plus[n,:,0]

      # update spectrum of first mode through Wiener filter of residuals
      u_hat_plus[n+1,:,k] = (f_hat_plus - sum_uk - lambda_hat[n,:]/2)/(1.
↪+Alpha[k]*(freqs - omega_plus[n,k])**2)

      # update first omega if not held at 0
      if not(DC):
          omega_plus[n+1,k] = np.dot(freqs[T//2:T],(abs(u_hat_plus[n+1, T//2:
↪T, k])**2))/np.sum(abs(u_hat_plus[n+1,T//2:T,k])**2)

      # update of any other mode
      for k in np.arange(1,K):
          #accumulator
          sum_uk = u_hat_plus[n+1,:,k-1] + sum_uk - u_hat_plus[n,:,k]
          # mode spectrum
          u_hat_plus[n+1,:,k] = (f_hat_plus - sum_uk - lambda_hat[n,:]/2)/
↪(1+Alpha[k]*(freqs - omega_plus[n,k])**2)
          # center frequencies
          omega_plus[n+1,k] = np.dot(freqs[T//2:T],(abs(u_hat_plus[n+1, T//2:
↪T, k])**2))/np.sum(abs(u_hat_plus[n+1,T//2:T,k])**2)

      # Dual ascent
      lambda_hat[n+1,:] = lambda_hat[n,:] + tau*(np.sum(u_hat_plus[n+1,:,:
↪],axis = 1) - f_hat_plus)

      # loop counter
      n = n+1

      # converged yet?
      uDiff = np.spacing(1)
      for i in range(K):
          uDiff = uDiff + (1/T)*np.dot((u_hat_plus[n,:,i]-u_hat_plus[n-1,:
↪,i]),np.conj((u_hat_plus[n,:,i]-u_hat_plus[n-1,:,i])))

      uDiff = np.abs(uDiff)

  #Postprocessing and cleanup

  #discard empty space if converged early
  Niter = np.min([Niter,n])
  omega = omega_plus[:Niter,:]
```

```
    idxs = np.flip(np.arange(1,T//2+1),axis = 0)
    # Signal reconstruction
    u_hat = np.zeros([T, K],dtype = complex)
    u_hat[T//2:T,:] = u_hat_plus[Niter-1,T//2:T,:]
    u_hat[idxs,:] = np.conj(u_hat_plus[Niter-1,T//2:T,:])
    u_hat[0,:] = np.conj(u_hat[-1,:])

    u = np.zeros([K,len(t)])
    for k in range(K):
        u[k,:] = np.real(np.fft.ifft(np.fft.ifftshift(u_hat[:,k])))

    # remove mirror part
    u = u[:,T//4:3*T//4]

    # recompute spectrum
    u_hat = np.zeros([u.shape[1],K],dtype = complex)
    for k in range(K):
        u_hat[:,k]=np.fft.fftshift(np.fft.fft(u[k,:]))


    for k in range(1,K+1):
        filename_ifft = "data_eeg_prop_2024_1S/{}_imf-%d.txt" .format(str(n).
↪zfill(3)) %(k)
        np.savetxt(filename_ifft, u[k-1,:])

    #recompute FFT spectrum
    u_hat = np.zeros((T//2, K),dtype=complex)

    for k in range(1,K+1):
        u_hat[:, k-1]= ((np.fft.fft(u[k-1,:])).conj()).T

    fft_amp = np.zeros((T//2, K), dtype = 'float64')
    for k in range(1,K+1):
        filename_fft = "data_eeg_prop_2024_1S/{}_fft-%d.txt" .format(str(n).
↪zfill(3)) %(k)
        np.savetxt(filename_fft, np.abs(u_hat[:,k-1]/ (128 / 2)))
        fft_amp[:, k-1] = np.abs(u_hat[:,k-1] / (128 / 2))

    fft_axis = np.linspace(0, 128, int(T/2))

    fig = plt.figure()

    plt.plot(signal)
    plt.show()
    filename1 = "data_eeg_prop_2024_1S/eeg.svg"
    fig.savefig(filename1)
```

```
    fig4 = plt.figure()
    N=len(signal)
    f_sig= fft(signal)
    plt.plot(fft_axis[0:int(T/4)], 2.0/N*abs(f_sig[0:int(N/2)]))
    plt.show()
    filename4 = "data_eeg_prop_2024_1S/spectrum.svg"
    fig4.savefig(filename4)

    for i in range(1,K+1):
         fig2 = plt.figure()
         plt.plot(u[i-1,:])
         plt.show()
         filename2 = "data_eeg_prop_2024_1S/{}_imf-%d.svg" .format(str(n).
 ↪zfill(3)) %(i)
         fig2.savefig(filename2)

    u_t = u.T

    return (u,u_t,u_hat,omega)
```

[18]:
```
alpha = 2000
tau = 0
DC = 0
init = 0
tol = 1e-7

signal=eeg_1024
K =  6
u,u_t,u_hat,omega = VMD(signal, alpha, tau, K, DC, init, tol)
```

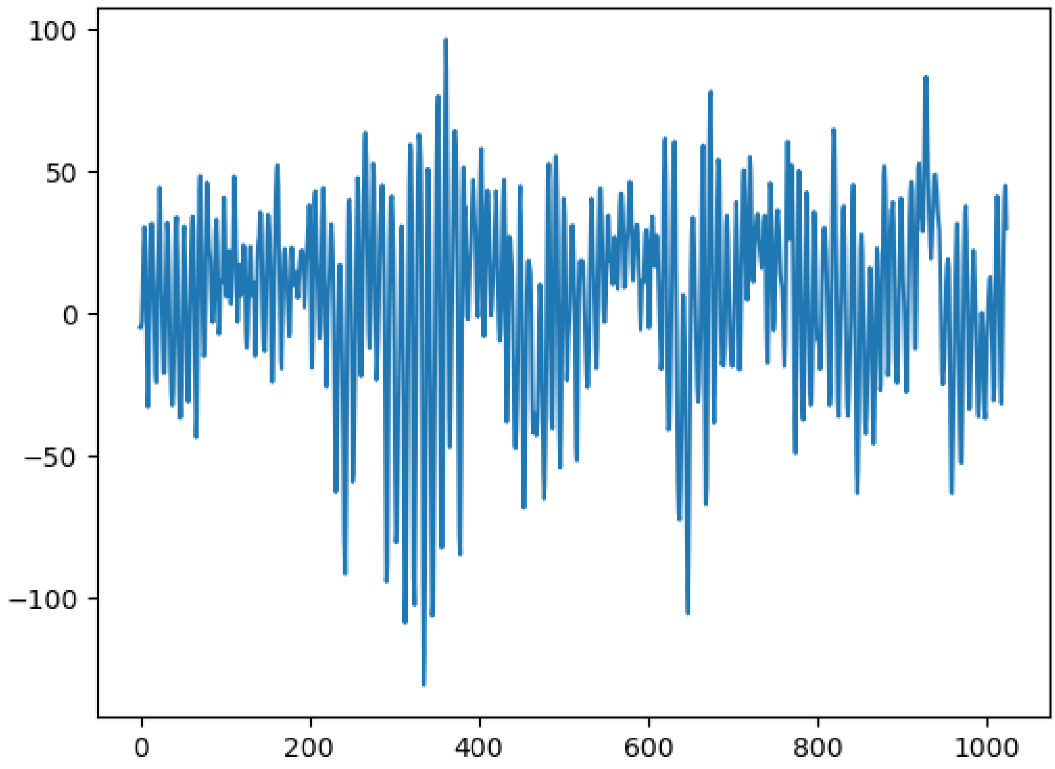
100
50
0
−50
−100
0
200
400
600
800
1000

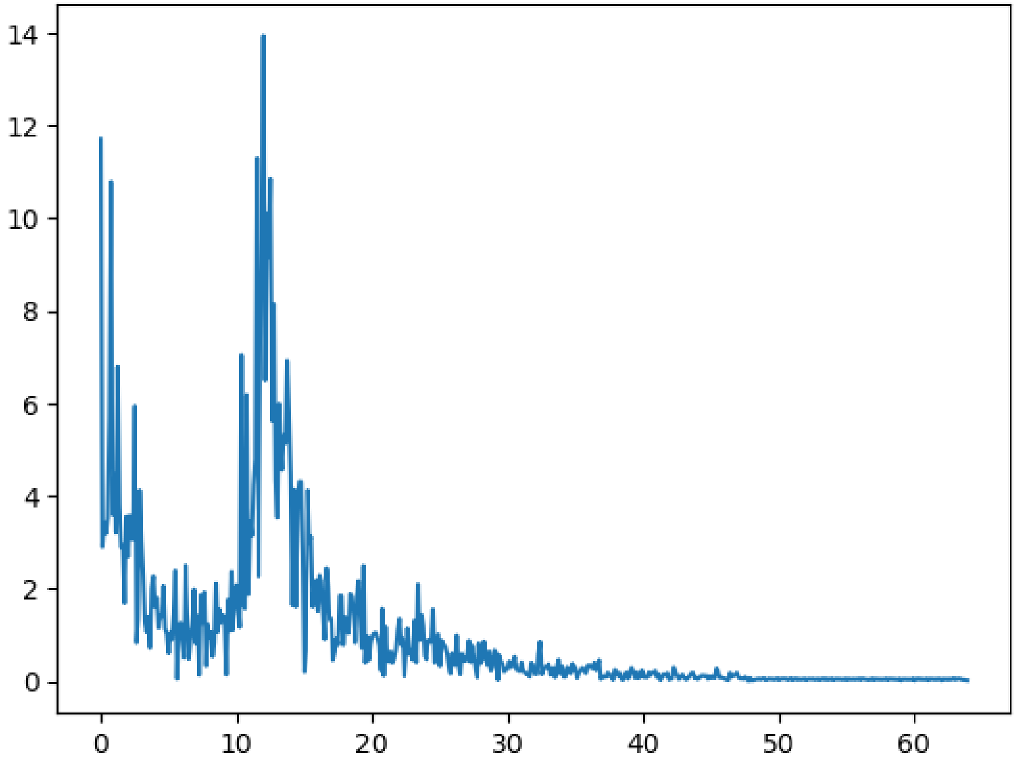
14
12
10
8
6
4
2
0
0
10
20
30
40
50
60

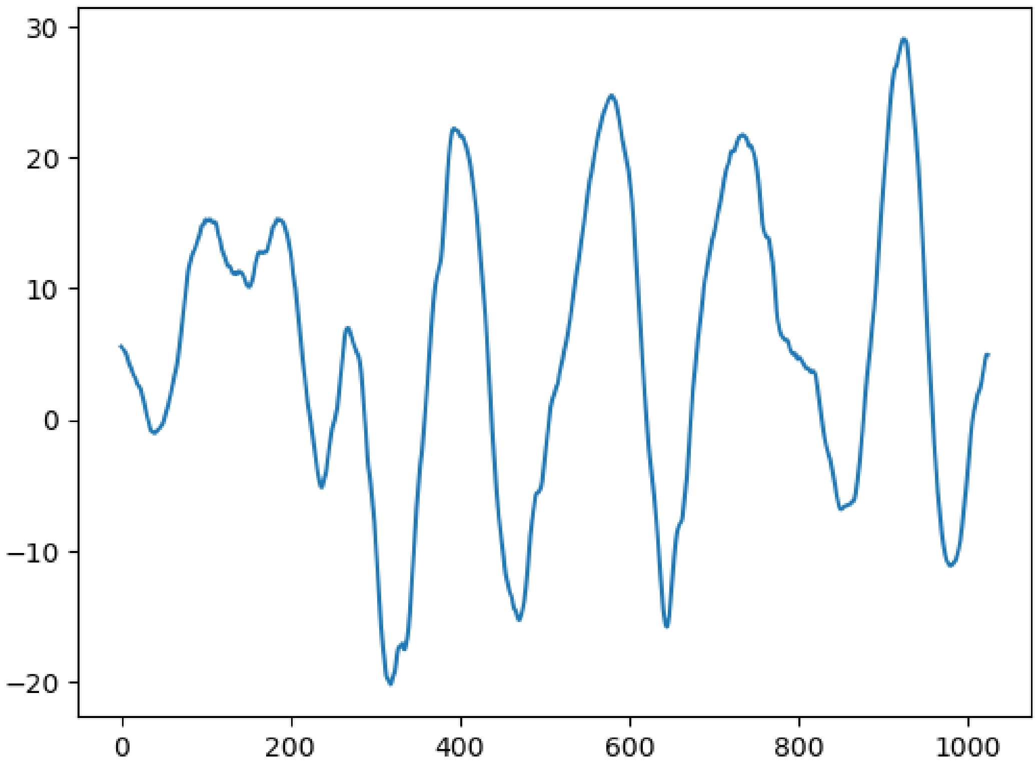
30
20
10
0
−10
−20
0
200
400
600
800
1000

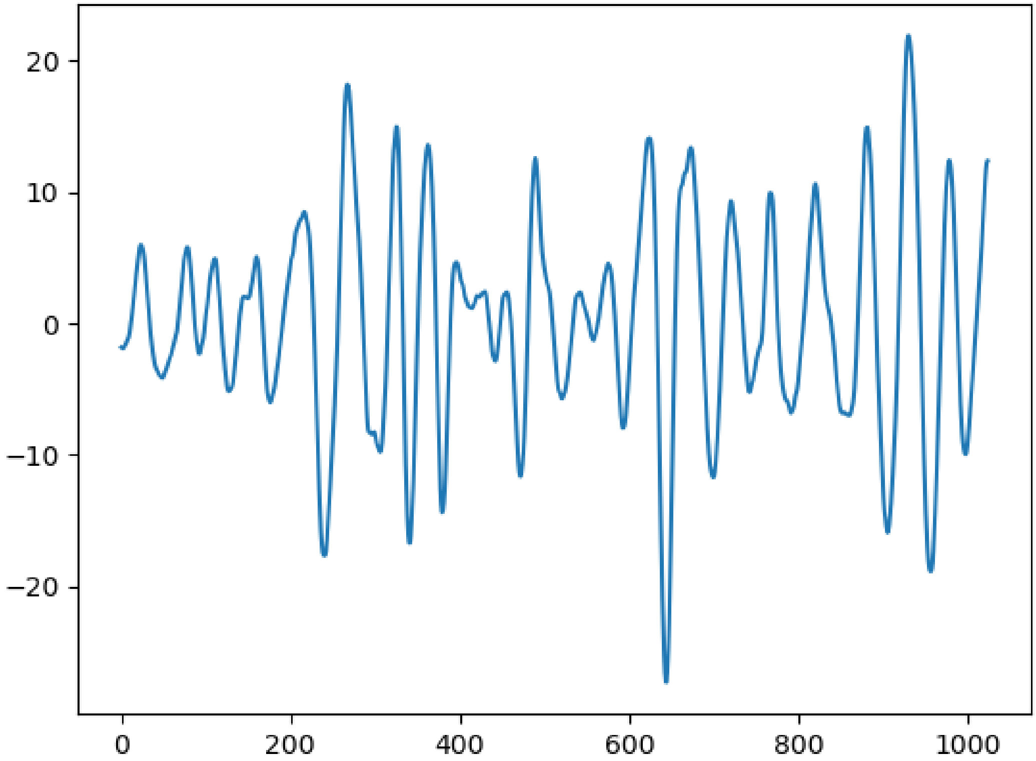
20
10
0
−10
−20
0
200
400
600
800
1000

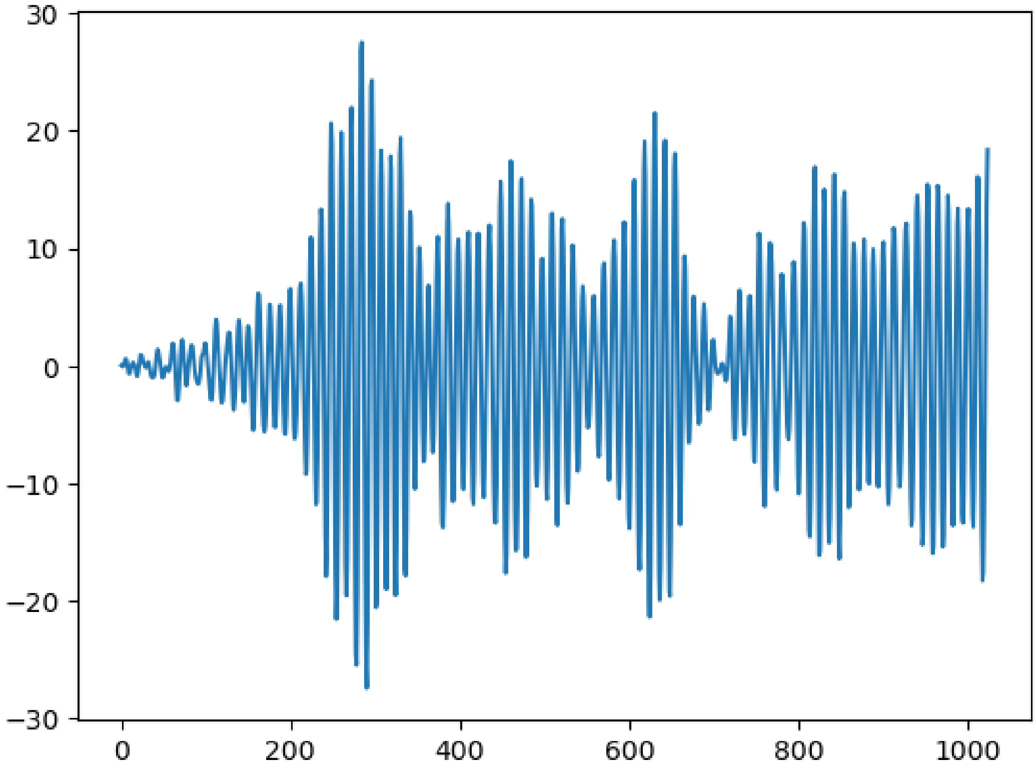
30
20
10
0
−10
−20
−30
0
200
400
600
800
1000

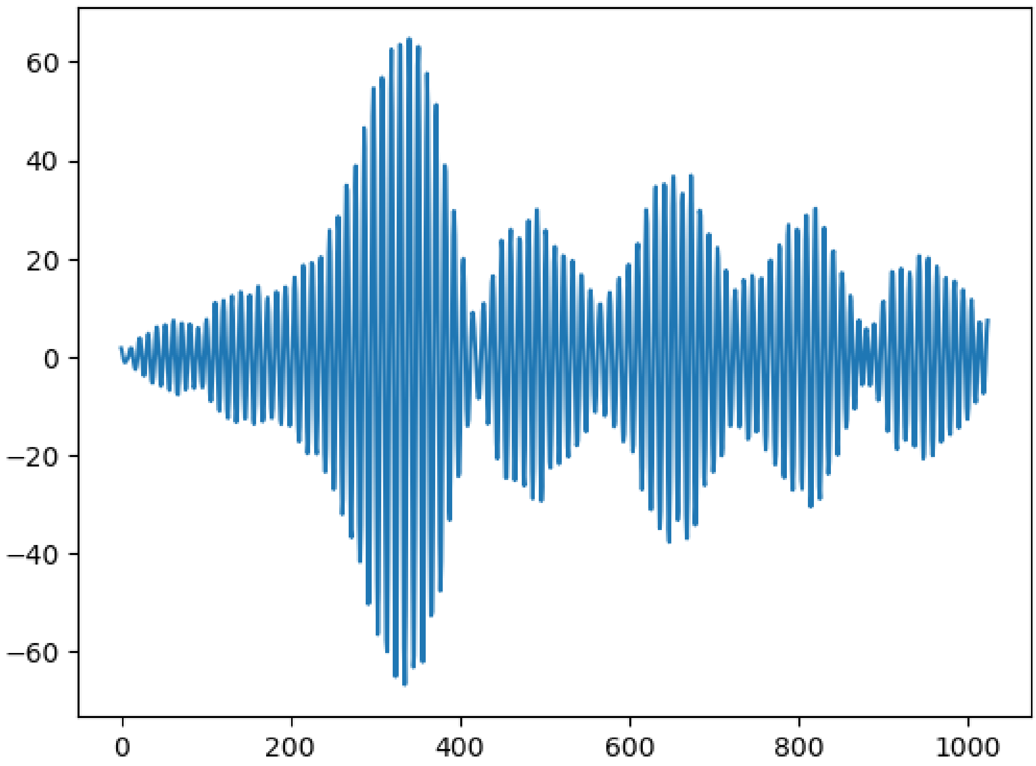
60
40
20
0
−20
−40
−60
0
200
400
600
800
1000

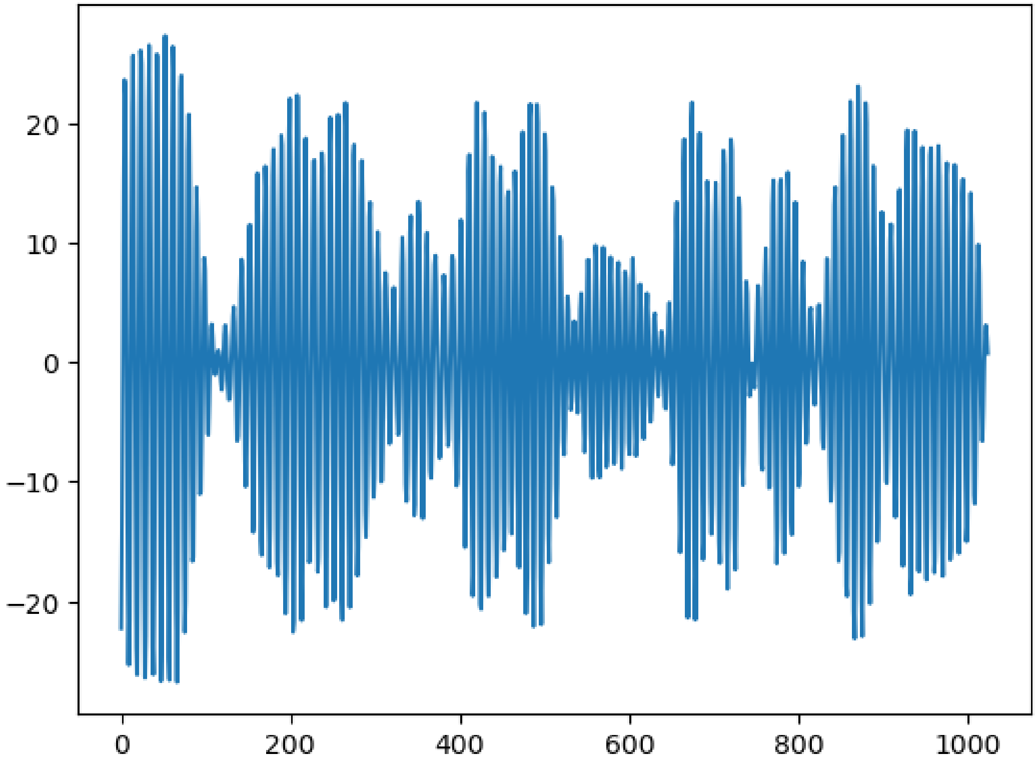
20
10
0
−10
−20
0
200
400
600
800
1000

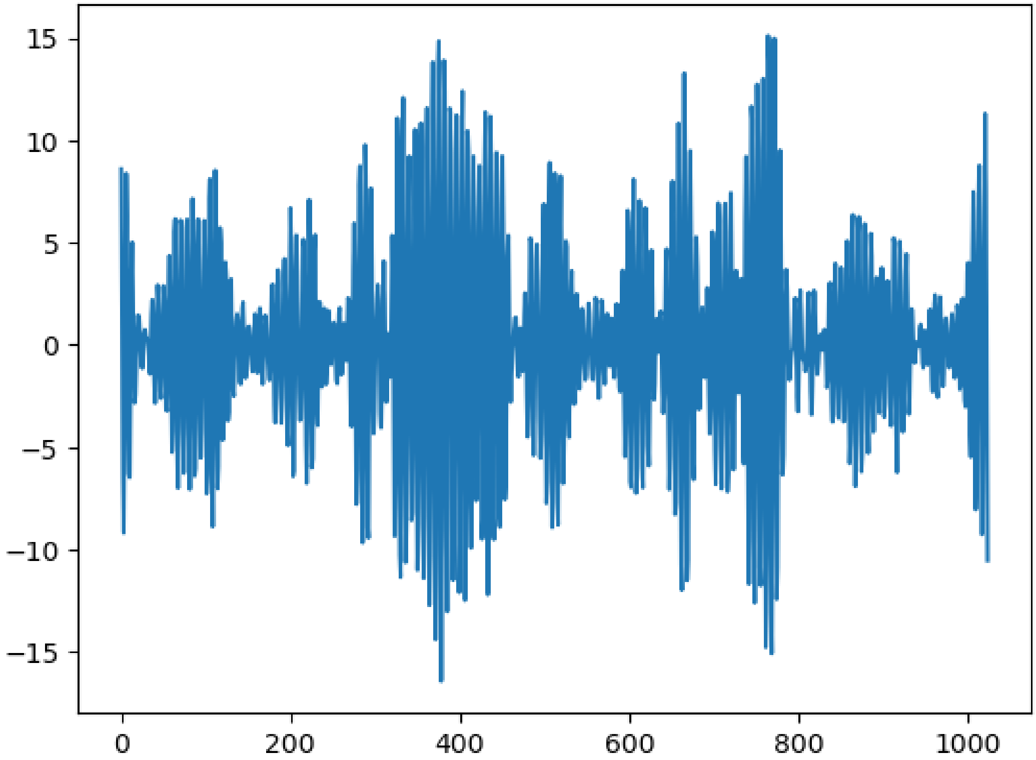
15
10
5
0
−5
−10
−15
0
200
400
600
800
1000

4 # EMD & VMD before GWO

[19]:
```
#pip install emd
```

[20]:
```
import emd
```

[21]:
```
# Get the default configuration for a sift
config = emd.sift.get_config('sift')
# Adjust the threshold for accepting an IMF
config['imf_opts/sd_thresh'] = 0.05
imf = emd.sift.sift(signal)
```

[22]:
```
emd.plotting.plot_imfs(imf, cmap=True)
plt.savefig('data_eeg_prop_2024_1S/emd.svg')
```

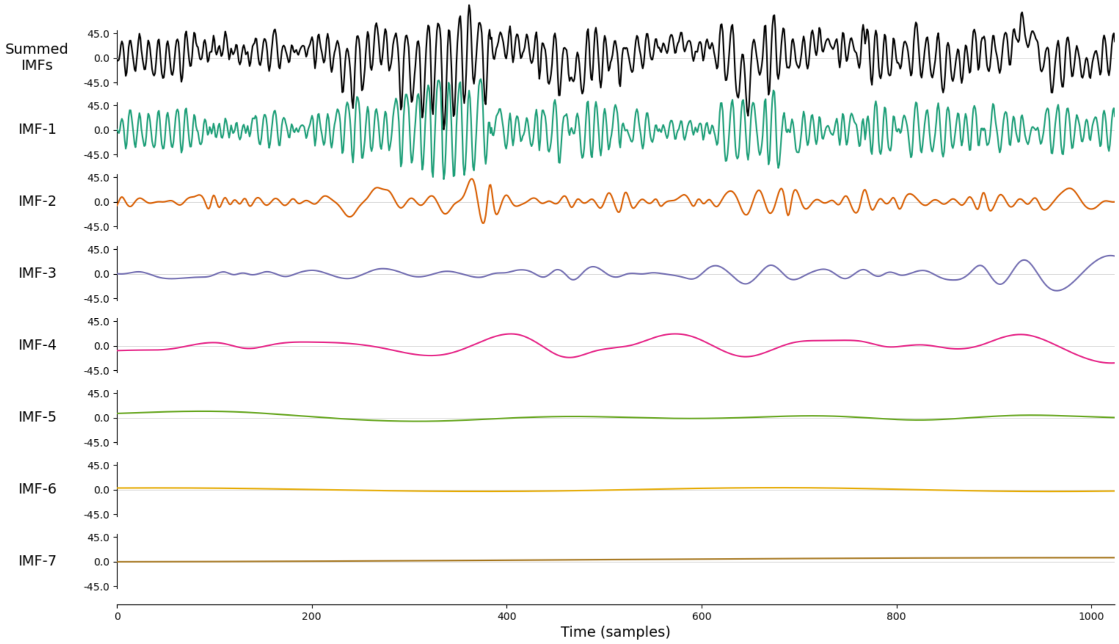

[23]:
```
emd.plotting.plot_imfs(u_t, cmap=True)
plt.savefig('data_eeg_prop_2024_1S/vmd.svg')
```

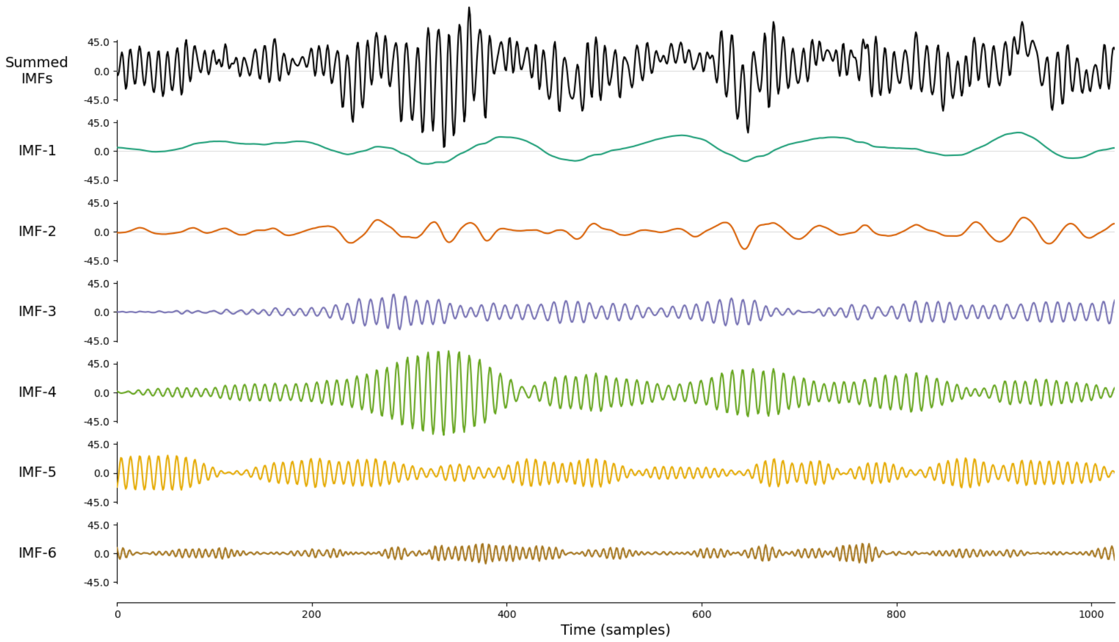

5 # GWO

6 # Preprocessing for GWO

[24]:
```
# python implementation of Grey wolf optimization (GWO)
# minimizing rastrigin and sphere function

import random
import math # cos() for Rastrigin
import copy # array-copying convenience
import sys          # max float
```

[25]:
```
def ent(data):
    ent = 0
    for i in range(data.size):
        ent += data[i]*np.log2(data[i])
    return -abs(ent)
```

[26]:
```
# wolf class
class wolf3:
    def __init__(self, fitness, minx1, maxx1, minx2, maxx2):
        self.rnd = random.Random(0)

        self.position1 = 0
```

```
        self.position2 = 0

        self.position1 = random.randint(minx1, maxx1)
        self.position2 = random.randint(minx2, maxx2)

        print("self.position1 = ", self.position1)
        print("self.position2 = ", self.position2)

        self.fitness = fitness(self.position1, self.position2) # curr fitness
```

[27]:
```
def VMD_wog(f, alpha, tau, K, DC, init, tol):

    """
    u,u_hat,omega = VMD(f, alpha, tau, K, DC, init, tol)
    Variational mode decomposition
    Python implementation by Vinícius Rezende Carvalho - vrcarva@gmail.com
    code based on Dominique Zosso's MATLAB code, available at:
    https://www.mathworks.com/matlabcentral/fileexchange/
↪44765-variational-mode-decomposition
    Original paper:
    Dragomiretskiy, K. and Zosso, D. (2014) ‘Variational Mode Decomposition’,
    IEEE Transactions on Signal Processing, 62(3), pp. 531-544. doi: 10.1109/
↪TSP.2013.2288675.


    Input and Parameters:
    ---------------------
    f       - the time domain signal (1D) to be decomposed
    alpha   - the balancing parameter of the data-fidelity constraint
    tau     - time-step of the dual ascent ( pick 0 for noise-slack )
    K       - the number of modes to be recovered
    DC      - true if the first mode is put and kept at DC (0-freq)
    init    - 0 = all omegas start at 0
                       1 = all omegas start uniformly distributed
                      2 = all omegas initialized randomly
    tol     - tolerance of convergence criterion; typically around 1e-6

    Output:
    -------
    u       - the collection of decomposed modes
    u_hat   - spectra of the modes
    omega   - estimated mode center-frequencies
    """

    if len(f)%2:
        f = f[:-1]
```

```python
    # Period and sampling frequency of input signal
    fs = 1./len(f)

    ltemp = len(f)//2
    fMirr =  np.append(np.flip(f[:ltemp],axis = 0),f)
    fMirr = np.append(fMirr,np.flip(f[-ltemp:],axis = 0))

    # Time Domain 0 to T (of mirrored signal)
    T = len(fMirr)
    t = np.arange(1,T+1)/T

    # Spectral Domain discretization
    freqs = t-0.5-(1/T)

    # Maximum number of iterations (if not converged yet, then it won't anyway)
    Niter = 500
    # For future generalizations: individual alpha for each mode
    Alpha = alpha*np.ones(K)

    # Construct and center f_hat
    f_hat = np.fft.fftshift((np.fft.fft(fMirr)))
    f_hat_plus = np.copy(f_hat) #copy f_hat
    f_hat_plus[:T//2] = 0

    # Initialization of omega_k
    omega_plus = np.zeros([Niter, K])


    if init == 1:
        for i in range(K):
            omega_plus[0,i] = (0.5/K)*(i)
    elif init == 2:
        omega_plus[0,:] = np.sort(np.exp(np.log(fs) + (np.log(0.5)-np.
↪log(fs))*np.random.rand(1,K)))
    else:
        omega_plus[0,:] = 0

    # if DC mode imposed, set its omega to 0
    if DC:
        omega_plus[0,0] = 0

    # start with empty dual variables
    lambda_hat = np.zeros([Niter, len(freqs)], dtype = complex)

    # other inits
    uDiff = tol+np.spacing(1) # update step
    n = 0 # loop counter
```

```python
    sum_uk = 0 # accumulator
    # matrix keeping track of every iterant // could be discarded for mem
    u_hat_plus = np.zeros([Niter, len(freqs), K],dtype=complex)

    #*** Main loop for iterative updates***

    while ( uDiff > tol and  n < Niter-1 ): # not converged and below iterations limit
        # update first mode accumulator
        k = 0
        sum_uk = u_hat_plus[n,:,K-1] + sum_uk - u_hat_plus[n,:,0]

        # update spectrum of first mode through Wiener filter of residuals
        u_hat_plus[n+1,:,k] = (f_hat_plus - sum_uk - lambda_hat[n,:]/2)/(1.+Alpha[k]*(freqs - omega_plus[n,k])**2)

        # update first omega if not held at 0
        if not(DC):
            omega_plus[n+1,k] = np.dot(freqs[T//2:T],(abs(u_hat_plus[n+1, T//2:T, k])**2))/np.sum(abs(u_hat_plus[n+1,T//2:T,k])**2)

        # update of any other mode
        for k in np.arange(1,K):
            #accumulator
            sum_uk = u_hat_plus[n+1,:,k-1] + sum_uk - u_hat_plus[n,:,k]
            # mode spectrum
            u_hat_plus[n+1,:,k] = (f_hat_plus - sum_uk - lambda_hat[n,:]/2)/(1+Alpha[k]*(freqs - omega_plus[n,k])**2)
            # center frequencies
            omega_plus[n+1,k] = np.dot(freqs[T//2:T],(abs(u_hat_plus[n+1, T//2:T, k])**2))/np.sum(abs(u_hat_plus[n+1,T//2:T,k])**2)

        # Dual ascent
        lambda_hat[n+1,:] = lambda_hat[n,:] + tau*(np.sum(u_hat_plus[n+1,:,:],axis = 1) - f_hat_plus)

        # loop counter
        n = n+1

        # converged yet?
        uDiff = np.spacing(1)
        for i in range(K):
            uDiff = uDiff + (1/T)*np.dot((u_hat_plus[n,:,i]-u_hat_plus[n-1,:,i]),np.conj((u_hat_plus[n,:,i]-u_hat_plus[n-1,:,i])))

        uDiff = np.abs(uDiff)
```

```
    #Postprocessing and cleanup

    #discard empty space if converged early
    Niter = np.min([Niter,n])
    omega = omega_plus[:Niter,:]

    idxs = np.flip(np.arange(1,T//2+1),axis = 0)
    # Signal reconstruction
    u_hat = np.zeros([T, K],dtype = complex)
    u_hat[T//2:T,:] = u_hat_plus[Niter-1,T//2:T,:]
    u_hat[idxs,:] = np.conj(u_hat_plus[Niter-1,T//2:T,:])
    u_hat[0,:] = np.conj(u_hat[-1,:])

    u = np.zeros([K,len(t)])
    for k in range(K):
        u[k,:] = np.real(np.fft.ifft(np.fft.ifftshift(u_hat[:,k])))

    # remove mirror part
    u = u[:,T//4:3*T//4]

    # recompute spectrum
    u_hat = np.zeros([u.shape[1],K],dtype = complex)
    for k in range(K):
        u_hat[:,k]=np.fft.fftshift(np.fft.fft(u[k,:]))

    u_t = u.T

    return (u,u_t,u_hat,omega)
```

[28]:
```
def vmd_gwo(_position1, _position2):
    from scipy.stats import entropy
    from scipy.signal import hilbert

    signal=eeg_1024

    dt = 1/128

    tau = 0
    DC = 0
    init = 0
    tol = 1e-7

    T1 = signal.size
    x_env_double = np.zeros((_position1, T1))
    entropy = np.zeros(_position1)
    entropy_double = np.zeros(_position1)
```

```
    p = np.zeros(T1)
    p_float = np.zeros(T1, dtype=float)

    x_env = []
    x_env = [0 for i in range(_position1)]

    # PF * 10x
    position_alpha = complex(_position2 * 10, 0)

    # Vmd class
    u,u_t,u_hat,omega = VMD_wog(signal, position_alpha, tau, _position1, DC,␣
 ↪init, tol)

    for k in range(T1):
        x_env_double[_position1-1][k] = 0.0

    for j in range(_position1):
        entropy[j] = 0.0

    for j in range(_position1):
        entropy_double[j] = 0.0

    for k in range(_position1):
        x_env[k] = np.abs(hilbert(u_t[:,k]))

    for k in range(_position1):
        for i in range(T1):
            p[i] = x_env[k][i] / np.sum(abs(x_env[k]))

        for i in range(T1):
            p_float[i] = float(p[i])

        #enttropy function
        entropy[k] = ent(p)

    #entropy_all = np.sum(entropy)/_position1
    entropy_max = np.amax(entropy)

    #return entropy_all
    return entropy_max
```

[29]:

```
# grey wolf optimization (GWO)
def gwo_KPF(fitness, max_iter, n, minx1, maxx1, minx2, maxx2):
        #rnd = random.Random(0)
        random.seed(0)

        fnew = 0.0
```

```
        # create n random wolves
        population = [ wolf3(fitness, minx1, maxx1, minx2, maxx2) for i in␣
↪range(n)]

        # On the basis of fitness values of wolves
        # sort the population I in asc order
        population = sorted(population, key = lambda temp: temp.fitness)

        # best 3 solutions will be called as
        # alpha, beta and gaama
        alpha_wolf, beta_wolf, gamma_wolf = copy.copy(population[: 3])
        print("pre_alpha_wolf = ", alpha_wolf.fitness)

        # main loop of gwo
        Iter = 0
        while Iter < max_iter:
            print("************************")
            print("Iter = ", Iter)
            # after every 10 iterations
            # print iteration number and best fitness value so far
            if Iter % 1 == 10 and Iter > 1:
                print("Iter = " + str(Iter) + " best fitness =:", alpha_wolf.
↪fitness)

            # linearly decreased from 2 to 0
            a = 2*(1 - Iter/max_iter)

            # updating each population member with the help of best three␣
↪members
            for i in range(n):
                print("------------------------")
                print("each population member: i = ", i)
                A1, A2, A3 = a * (2 * random.random() - 1), a * (
                  2 * random.random() - 1), a * (2 * random.random() - 1)

                B1, B2, B3 = a * (2 * random.random() - 1), a * (
                  2 * random.random() - 1), a * (2 * random.random() - 1)

                C1, C2, C3 = 2 * random.random(), 2*random.random(), 2*random.
↪random()
                D1, D2, D3 = 2 * random.random(), 2*random.random(), 2*random.
↪random()

                X1 = 0.0
                X2 = 0.0
                X3 = 0.0
                Xnew = 0.0
```

```
            Ynew = 0.0

            #for j in range(position1):
            X1 = int(alpha_wolf.position1 - A1 * abs(
                  C1 * alpha_wolf.position1 - population[i].position1))
            X2 = int(beta_wolf.position1 - A2 * abs(
                  C2 *  beta_wolf.position1 - population[i].position1))
            X3 = int(gamma_wolf.position1 - A3 * abs(
                  C3 * gamma_wolf.position1 - population[i].position1))
            Xnew += X1 + X2 + X3

            #for j in range(position1):
            Y1 = int(alpha_wolf.position2 - B1 * abs(
                  D1 * alpha_wolf.position2 - population[i].position2))
            Y2 = int(beta_wolf.position2 - B2 * abs(
                  D2 *  beta_wolf.position2 - population[i].position2))
            Y3 = int(gamma_wolf.position2 - B3 * abs(
                  D3 * gamma_wolf.position2 - population[i].position2))
            Ynew += Y1 + Y2 + Y3

            #for j in range(dim):
            Xnew= int(Xnew/3.0)
            Ynew= int(Ynew/3.0)

            if (Xnew>=minx1 and Xnew<=maxx1 and Ynew>=minx2 and␣
↪Ynew<=maxx2):
                # fitness calculation of new solution
                fnew = fitness(Xnew, Ynew);

                # greedy selection
                if fnew < population[i].fitness:
                    population[i].position1 = Xnew
                    population[i].position2 = Ynew
                    population[i].fitness = fnew

        for i in range(n):
            print("population-preordered", i, ":", population[i].position1,␣
↪", ", population[i].position2, ", ",  population[i].fitness)

        population = sorted(population, key = lambda temp: temp.fitness)
        print("- - - - - - - - - - - - - ")

        for i in range(n):
            print("population-postordered", i, ":", population[i].
↪position1, ", ", population[i].position2, ", ",  population[i].fitness)

        # best 3 solutions will be called as
```

```
            # alpha, beta and gaama
            alpha_wolf, beta_wolf, gamma_wolf = copy.copy(population[: 3])
            print("optimized alpha_wolf = ", alpha_wolf.fitness)

            Iter+= 1
        # end-while

        # returning the best solution
        return alpha_wolf.position1, alpha_wolf.position2

#----------------------------
```

[30]:
```
x_env_ori = np.abs(hilbert(eeg_1024))
x_env_ori_norm = x_env_ori/np.sum(x_env_ori)

entropy_ori = ent(x_env_ori_norm)
entropy_ori
```

[30]: -9.788829994422969

7 # Driver code for rastrigin function

[31]:
```
print("\nBegin grey wolf optimization on rastrigin function\n")
#dim = 3
fitness = vmd_gwo
```

```
Begin grey wolf optimization on rastrigin function
```

[32]:
```
num_particles =20
max_iter = 20

print("Setting num_particles = " + str(num_particles))
print("Setting max_iter    = " + str(max_iter))
print("\nStarting GWO algorithm\n")
```

```
Setting num_particles = 20
Setting max_iter    = 20

Starting GWO algorithm
```

[33]:
```
#PF is 10%PF. so multiplying 10x after GWO
best_position1, best_position2 = gwo_KPF(fitness, max_iter, num_particles, 2,␣
 ↪6, 1, 500)
```

```python
print("\nGWO completed\n")
print("\nBest solution found:")
print("\nBest solution 1:", best_position1)
print("\nBest solution 2:", best_position2)

err = fitness(best_position1, best_position2)
print("fitness of best solution =:", err)
print("\nEnd GWO\n")
```

```
self.position1 =  5
self.position2 =  389
self.position1 =  5
self.position2 =  21
self.position1 =  4
self.position2 =  495
self.position1 =  6
self.position2 =  249
self.position1 =  5
self.position2 =  471
self.position1 =  4
self.position2 =  496
self.position1 =  5
self.position2 =  184
self.position1 =  6
self.position2 =  457
self.position1 =  3
self.position2 =  259
self.position1 =  3
self.position2 =  145
self.position1 =  3
self.position2 =  387
self.position1 =  2
self.position2 =  317
self.position1 =  4
self.position2 =  466
self.position1 =  6
self.position2 =  362
self.position1 =  6
self.position2 =  462
self.position1 =  3
self.position2 =  159
self.position1 =  2
self.position2 =  374
self.position1 =  2
self.position2 =  461
self.position1 =  4
self.position2 =  242
self.position1 =  6
```

```
self.position2 =  52
pre_alpha_wolf =  -9.769566821006258
************************
Iter =  0
------------------------
each population member: i =  0
------------------------
each population member: i =  1
------------------------
each population member: i =  2
------------------------
each population member: i =  3
------------------------
each population member: i =  4
------------------------
each population member: i =  5
------------------------
each population member: i =  6
------------------------
each population member: i =  7
------------------------
each population member: i =  8
------------------------
each population member: i =  9
------------------------
each population member: i =  10
------------------------
each population member: i =  11
------------------------
each population member: i =  12
------------------------
each population member: i =  13
------------------------
each population member: i =  14
------------------------
each population member: i =  15
------------------------
each population member: i =  16
------------------------
each population member: i =  17
------------------------
each population member: i =  18
------------------------
each population member: i =  19
population-preordered 0 : 2 ,  317 ,  -9.769566821006258
population-preordered 1 : 2 ,  374 ,  -9.76664682056081
population-preordered 2 : 2 ,  461 ,  -9.763080124206187
population-preordered 3 : 3 ,  387 ,  -9.753822531261656
```

```
population-preordered 4 : 6 ,  362 ,  -9.690041530155908
population-preordered 5 : 6 ,  457 ,  -9.68863297041823
population-preordered 6 : 6 ,  462 ,  -9.688605962707262
population-preordered 7 : 6 ,  52 ,  -9.686916898663338
population-preordered 8 : 2 ,  318 ,  -9.769508205348043
population-preordered 9 : 5 ,  184 ,  -9.67962825060588
population-preordered 10 : 6 ,  249 ,  -9.672388124205348
population-preordered 11 : 2 ,  107 ,  -9.793257573381204
population-preordered 12 : 2 ,  358 ,  -9.767398391769605
population-preordered 13 : 2 ,  295 ,  -9.770966493739449
population-preordered 14 : 2 ,  192 ,  -9.781195825624554
population-preordered 15 : 4 ,  242 ,  -9.64228285438766
population-preordered 16 : 3 ,  259 ,  -9.640751543673383
population-preordered 17 : 2 ,  468 ,  -9.762820975641366
population-preordered 18 : 3 ,  159 ,  -9.63874779621141
population-preordered 19 : 5 ,  21 ,  -9.627858352999036
- - - - - - - - - - - -
population-postordered 0 : 2 ,  107 ,  -9.793257573381204
population-postordered 1 : 2 ,  192 ,  -9.781195825624554
population-postordered 2 : 2 ,  295 ,  -9.770966493739449
population-postordered 3 : 2 ,  317 ,  -9.769566821006258
population-postordered 4 : 2 ,  318 ,  -9.769508205348043
population-postordered 5 : 2 ,  358 ,  -9.767398391769605
population-postordered 6 : 2 ,  374 ,  -9.76664682056081
population-postordered 7 : 2 ,  461 ,  -9.763080124206187
population-postordered 8 : 2 ,  468 ,  -9.762820975641366
population-postordered 9 : 3 ,  387 ,  -9.753822531261656
population-postordered 10 : 6 ,  362 ,  -9.690041530155908
population-postordered 11 : 6 ,  457 ,  -9.68863297041823
population-postordered 12 : 6 ,  462 ,  -9.688605962707262
population-postordered 13 : 6 ,  52 ,  -9.686916898663338
population-postordered 14 : 5 ,  184 ,  -9.67962825060588
population-postordered 15 : 6 ,  249 ,  -9.672388124205348
population-postordered 16 : 4 ,  242 ,  -9.64228285438766
population-postordered 17 : 3 ,  259 ,  -9.640751543673383
population-postordered 18 : 3 ,  159 ,  -9.63874779621141
population-postordered 19 : 5 ,  21 ,  -9.627858352999036
optimized alpha_wolf =  -9.793257573381204
************************
Iter =  1
------------------------
each population member: i =  0
------------------------
each population member: i =  1
------------------------
each population member: i =  2
------------------------
each population member: i =  3
```

```
------------------------
each population member: i =  4
------------------------
each population member: i =  5
------------------------
each population member: i =  6
------------------------
each population member: i =  7
------------------------
each population member: i =  8
------------------------
each population member: i =  9
------------------------
each population member: i =  10
------------------------
each population member: i =  11
------------------------
each population member: i =  12
------------------------
each population member: i =  13
------------------------
each population member: i =  14
------------------------
each population member: i =  15
------------------------
each population member: i =  16
------------------------
each population member: i =  17
------------------------
each population member: i =  18
------------------------
each population member: i =  19
population-preordered 0 : 2 ,  107 ,  -9.793257573381204
population-preordered 1 : 2 ,  182 ,  -9.782432944575818
population-preordered 2 : 2 ,  189 ,  -9.781562529971154
population-preordered 3 : 2 ,  317 ,  -9.769566821006258
population-preordered 4 : 2 ,  318 ,  -9.769508205348043
population-preordered 5 : 2 ,  358 ,  -9.767398391769605
population-preordered 6 : 2 ,  374 ,  -9.76664682056081
population-preordered 7 : 2 ,  461 ,  -9.763080124206187
population-preordered 8 : 2 ,  468 ,  -9.762820975641366
population-preordered 9 : 3 ,  387 ,  -9.753822531261656
population-preordered 10 : 6 ,  362 ,  -9.690041530155908
population-preordered 11 : 6 ,  457 ,  -9.68863297041823
population-preordered 12 : 6 ,  462 ,  -9.688605962707262
population-preordered 13 : 2 ,  224 ,  -9.77751659809676
population-preordered 14 : 2 ,  106 ,  -9.793426915085258
population-preordered 15 : 6 ,  249 ,  -9.672388124205348
```

```
population-preordered 16 : 4 ,  242 ,  -9.64228285438766
population-preordered 17 : 3 ,  259 ,  -9.640751543673383
population-preordered 18 : 3 ,  159 ,  -9.63874779621141
population-preordered 19 : 5 ,  21 ,  -9.627858352999036
- - - - - - - - - - - -
population-postordered 0 : 2 ,  106 ,  -9.793426915085258
population-postordered 1 : 2 ,  107 ,  -9.793257573381204
population-postordered 2 : 2 ,  182 ,  -9.782432944575818
population-postordered 3 : 2 ,  189 ,  -9.781562529971154
population-postordered 4 : 2 ,  224 ,  -9.77751659809676
population-postordered 5 : 2 ,  317 ,  -9.769566821006258
population-postordered 6 : 2 ,  318 ,  -9.769508205348043
population-postordered 7 : 2 ,  358 ,  -9.767398391769605
population-postordered 8 : 2 ,  374 ,  -9.76664682056081
population-postordered 9 : 2 ,  461 ,  -9.763080124206187
population-postordered 10 : 2 ,  468 ,  -9.762820975641366
population-postordered 11 : 3 ,  387 ,  -9.753822531261656
population-postordered 12 : 6 ,  362 ,  -9.690041530155908
population-postordered 13 : 6 ,  457 ,  -9.68863297041823
population-postordered 14 : 6 ,  462 ,  -9.688605962707262
population-postordered 15 : 6 ,  249 ,  -9.672388124205348
population-postordered 16 : 4 ,  242 ,  -9.64228285438766
population-postordered 17 : 3 ,  259 ,  -9.640751543673383
population-postordered 18 : 3 ,  159 ,  -9.63874779621141
population-postordered 19 : 5 ,  21 ,  -9.627858352999036
optimized alpha_wolf =  -9.793426915085258
************************
Iter =  2
------------------------
each population member: i =  0
------------------------
each population member: i =  1
------------------------
each population member: i =  2
------------------------
each population member: i =  3
------------------------
each population member: i =  4
------------------------
each population member: i =  5
------------------------
each population member: i =  6
------------------------
each population member: i =  7
------------------------
each population member: i =  8
------------------------
each population member: i =  9
```

```
------------------------
each population member: i =  10
------------------------
each population member: i =  11
------------------------
each population member: i =  12
------------------------
each population member: i =  13
------------------------
each population member: i =  14
------------------------
each population member: i =  15
------------------------
each population member: i =  16
------------------------
each population member: i =  17
------------------------
each population member: i =  18
------------------------
each population member: i =  19
population-preordered 0 : 2 ,  72 ,  -9.799457752354563
population-preordered 1 : 2 ,  107 ,  -9.793257573381204
population-preordered 2 : 2 ,  182 ,  -9.782432944575818
population-preordered 3 : 2 ,  189 ,  -9.781562529971154
population-preordered 4 : 2 ,  224 ,  -9.77751659809676
population-preordered 5 : 2 ,  83 ,  -9.79745853555857
population-preordered 6 : 2 ,  318 ,  -9.769508205348043
population-preordered 7 : 2 ,  213 ,  -9.778733716594184
population-preordered 8 : 2 ,  374 ,  -9.76664682056081
population-preordered 9 : 2 ,  461 ,  -9.763080124206187
population-preordered 10 : 2 ,  468 ,  -9.762820975641366
population-preordered 11 : 3 ,  387 ,  -9.753822531261656
population-preordered 12 : 2 ,  271 ,  -9.772885956337182
population-preordered 13 : 6 ,  457 ,  -9.68863297041823
population-preordered 14 : 2 ,  42 ,  -9.804892425277657
population-preordered 15 : 6 ,  249 ,  -9.672388124205348
population-preordered 16 : 4 ,  242 ,  -9.64228285438766
population-preordered 17 : 3 ,  259 ,  -9.640751543673383
population-preordered 18 : 3 ,  262 ,  -9.640869179516123
population-preordered 19 : 5 ,  21 ,  -9.627858352999036
- - - - - - - - - - - - -
population-postordered 0 : 2 ,  42 ,  -9.804892425277657
population-postordered 1 : 2 ,  72 ,  -9.799457752354563
population-postordered 2 : 2 ,  83 ,  -9.79745853555857
population-postordered 3 : 2 ,  107 ,  -9.793257573381204
population-postordered 4 : 2 ,  182 ,  -9.782432944575818
population-postordered 5 : 2 ,  189 ,  -9.781562529971154
population-postordered 6 : 2 ,  213 ,  -9.778733716594184
```

```
population-postordered 7 : 2 ,  224 ,  -9.77751659809676
population-postordered 8 : 2 ,  271 ,  -9.772885956337182
population-postordered 9 : 2 ,  318 ,  -9.769508205348043
population-postordered 10 : 2 ,  374 ,  -9.76664682056081
population-postordered 11 : 2 ,  461 ,  -9.763080124206187
population-postordered 12 : 2 ,  468 ,  -9.762820975641366
population-postordered 13 : 3 ,  387 ,  -9.753822531261656
population-postordered 14 : 6 ,  457 ,  -9.68863297041823
population-postordered 15 : 6 ,  249 ,  -9.672388124205348
population-postordered 16 : 4 ,  242 ,  -9.64228285438766
population-postordered 17 : 3 ,  262 ,  -9.640869179516123
population-postordered 18 : 3 ,  259 ,  -9.640751543673383
population-postordered 19 : 5 ,  21 ,  -9.627858352999036
optimized alpha_wolf =  -9.804892425277657
************************
Iter =  3
------------------------
each population member: i =  0
------------------------
each population member: i =  1
------------------------
each population member: i =  2
------------------------
each population member: i =  3
------------------------
each population member: i =  4
------------------------
each population member: i =  5
------------------------
each population member: i =  6
------------------------
each population member: i =  7
------------------------
each population member: i =  8
------------------------
each population member: i =  9
------------------------
each population member: i =  10
------------------------
each population member: i =  11
------------------------
each population member: i =  12
------------------------
each population member: i =  13
------------------------
each population member: i =  14
------------------------
each population member: i =  15
```

```
------------------------
each population member: i =  16
------------------------
each population member: i =  17
------------------------
each population member: i =  18
------------------------
each population member: i =  19
population-preordered 0 : 2 ,  42 ,  -9.804892425277657
population-preordered 1 : 2 ,  72 ,  -9.799457752354563
population-preordered 2 : 2 ,  83 ,  -9.79745853555857
population-preordered 3 : 2 ,  107 ,  -9.793257573381204
population-preordered 4 : 2 ,  182 ,  -9.782432944575818
population-preordered 5 : 2 ,  189 ,  -9.781562529971154
population-preordered 6 : 2 ,  45 ,  -9.804369557073995
population-preordered 7 : 2 ,  224 ,  -9.77751659809676
population-preordered 8 : 2 ,  271 ,  -9.772885956337182
population-preordered 9 : 2 ,  286 ,  -9.771616406685204
population-preordered 10 : 2 ,  374 ,  -9.76664682056081
population-preordered 11 : 2 ,  224 ,  -9.77751659809676
population-preordered 12 : 2 ,  468 ,  -9.762820975641366
population-preordered 13 : 3 ,  387 ,  -9.753822531261656
population-preordered 14 : 6 ,  457 ,  -9.68863297041823
population-preordered 15 : 6 ,  249 ,  -9.672388124205348
population-preordered 16 : 4 ,  242 ,  -9.64228285438766
population-preordered 17 : 3 ,  262 ,  -9.640869179516123
population-preordered 18 : 3 ,  259 ,  -9.640751543673383
population-preordered 19 : 2 ,  41 ,  -9.80506435788289
- - - - - - - - - - - -
population-postordered 0 : 2 ,  41 ,  -9.80506435788289
population-postordered 1 : 2 ,  42 ,  -9.804892425277657
population-postordered 2 : 2 ,  45 ,  -9.804369557073995
population-postordered 3 : 2 ,  72 ,  -9.799457752354563
population-postordered 4 : 2 ,  83 ,  -9.79745853555857
population-postordered 5 : 2 ,  107 ,  -9.793257573381204
population-postordered 6 : 2 ,  182 ,  -9.782432944575818
population-postordered 7 : 2 ,  189 ,  -9.781562529971154
population-postordered 8 : 2 ,  224 ,  -9.77751659809676
population-postordered 9 : 2 ,  224 ,  -9.77751659809676
population-postordered 10 : 2 ,  271 ,  -9.772885956337182
population-postordered 11 : 2 ,  286 ,  -9.771616406685204
population-postordered 12 : 2 ,  374 ,  -9.76664682056081
population-postordered 13 : 2 ,  468 ,  -9.762820975641366
population-postordered 14 : 3 ,  387 ,  -9.753822531261656
population-postordered 15 : 6 ,  457 ,  -9.68863297041823
population-postordered 16 : 6 ,  249 ,  -9.672388124205348
population-postordered 17 : 4 ,  242 ,  -9.64228285438766
population-postordered 18 : 3 ,  262 ,  -9.640869179516123
```

```
population-postordered 19 : 3 ,  259 ,  -9.640751543673383
optimized alpha_wolf =  -9.80506435788289
************************
Iter =  4
------------------------
each population member: i =  0
------------------------
each population member: i =  1
------------------------
each population member: i =  2
------------------------
each population member: i =  3
------------------------
each population member: i =  4
------------------------
each population member: i =  5
------------------------
each population member: i =  6
------------------------
each population member: i =  7
------------------------
each population member: i =  8
------------------------
each population member: i =  9
------------------------
each population member: i =  10
------------------------
each population member: i =  11
------------------------
each population member: i =  12
------------------------
each population member: i =  13
------------------------
each population member: i =  14
------------------------
each population member: i =  15
------------------------
each population member: i =  16
------------------------
each population member: i =  17
------------------------
each population member: i =  18
------------------------
each population member: i =  19
population-preordered 0 : 2 ,  41 ,  -9.80506435788289
population-preordered 1 : 2 ,  42 ,  -9.804892425277657
population-preordered 2 : 2 ,  45 ,  -9.804369557073995
population-preordered 3 : 2 ,  47 ,  -9.804016015787624
```

```
population-preordered 4 : 2 ,  83 ,  -9.79745853555857
population-preordered 5 : 2 ,  58 ,  -9.802026640074809
population-preordered 6 : 2 ,  182 ,  -9.782432944575818
population-preordered 7 : 2 ,  189 ,  -9.781562529971154
population-preordered 8 : 2 ,  224 ,  -9.77751659809676
population-preordered 9 : 2 ,  40 ,  -9.805234983898817
population-preordered 10 : 2 ,  37 ,  -9.805737779102293
population-preordered 11 : 2 ,  286 ,  -9.771616406685204
population-preordered 12 : 2 ,  374 ,  -9.76664682056081
population-preordered 13 : 2 ,  468 ,  -9.762820975641366
population-preordered 14 : 3 ,  387 ,  -9.753822531261656
population-preordered 15 : 6 ,  457 ,  -9.68863297041823
population-preordered 16 : 6 ,  249 ,  -9.672388124205348
population-preordered 17 : 4 ,  242 ,  -9.64228285438766
population-preordered 18 : 3 ,  262 ,  -9.640869179516123
population-preordered 19 : 3 ,  259 ,  -9.640751543673383
- - - - - - - - - - - -
population-postordered 0 : 2 ,  37 ,  -9.805737779102293
population-postordered 1 : 2 ,  40 ,  -9.805234983898817
population-postordered 2 : 2 ,  41 ,  -9.80506435788289
population-postordered 3 : 2 ,  42 ,  -9.804892425277657
population-postordered 4 : 2 ,  45 ,  -9.804369557073995
population-postordered 5 : 2 ,  47 ,  -9.804016015787624
population-postordered 6 : 2 ,  58 ,  -9.802026640074809
population-postordered 7 : 2 ,  83 ,  -9.79745853555857
population-postordered 8 : 2 ,  182 ,  -9.782432944575818
population-postordered 9 : 2 ,  189 ,  -9.781562529971154
population-postordered 10 : 2 ,  224 ,  -9.77751659809676
population-postordered 11 : 2 ,  286 ,  -9.771616406685204
population-postordered 12 : 2 ,  374 ,  -9.76664682056081
population-postordered 13 : 2 ,  468 ,  -9.762820975641366
population-postordered 14 : 3 ,  387 ,  -9.753822531261656
population-postordered 15 : 6 ,  457 ,  -9.68863297041823
population-postordered 16 : 6 ,  249 ,  -9.672388124205348
population-postordered 17 : 4 ,  242 ,  -9.64228285438766
population-postordered 18 : 3 ,  262 ,  -9.640869179516123
population-postordered 19 : 3 ,  259 ,  -9.640751543673383
optimized alpha_wolf =  -9.805737779102293
************************
Iter =  5
------------------------
each population member: i =  0
------------------------
each population member: i =  1
------------------------
each population member: i =  2
------------------------
each population member: i =  3
```

```
------------------------
each population member: i =  4
------------------------
each population member: i =  5
------------------------
each population member: i =  6
------------------------
each population member: i =  7
------------------------
each population member: i =  8
------------------------
each population member: i =  9
------------------------
each population member: i =  10
------------------------
each population member: i =  11
------------------------
each population member: i =  12
------------------------
each population member: i =  13
------------------------
each population member: i =  14
------------------------
each population member: i =  15
------------------------
each population member: i =  16
------------------------
each population member: i =  17
------------------------
each population member: i =  18
------------------------
each population member: i =  19
population-preordered 0 : 2 ,  37 ,  -9.805737779102293
population-preordered 1 : 2 ,  40 ,  -9.805234983898817
population-preordered 2 : 2 ,  41 ,  -9.80506435788289
population-preordered 3 : 2 ,  42 ,  -9.804892425277657
population-preordered 4 : 2 ,  45 ,  -9.804369557073995
population-preordered 5 : 2 ,  35 ,  -9.806064199003348
population-preordered 6 : 2 ,  58 ,  -9.802026640074809
population-preordered 7 : 2 ,  41 ,  -9.80506435788289
population-preordered 8 : 2 ,  182 ,  -9.782432944575818
population-preordered 9 : 2 ,  10 ,  -9.808713154776056
population-preordered 10 : 2 ,  224 ,  -9.77751659809676
population-preordered 11 : 2 ,  286 ,  -9.771616406685204
population-preordered 12 : 2 ,  159 ,  -9.785441390101083
population-preordered 13 : 2 ,  468 ,  -9.762820975641366
population-preordered 14 : 3 ,  387 ,  -9.753822531261656
population-preordered 15 : 6 ,  457 ,  -9.68863297041823
```

```
population-preordered 16 : 6 ,  249 ,  -9.672388124205348
population-preordered 17 : 3 ,  45 ,  -9.707773048432884
population-preordered 18 : 3 ,  262 ,  -9.640869179516123
population-preordered 19 : 3 ,  259 ,  -9.640751543673383
- - - - - - - - - - - -
population-postordered 0 : 2 ,  10 ,  -9.808713154776056
population-postordered 1 : 2 ,  35 ,  -9.806064199003348
population-postordered 2 : 2 ,  37 ,  -9.805737779102293
population-postordered 3 : 2 ,  40 ,  -9.805234983898817
population-postordered 4 : 2 ,  41 ,  -9.80506435788289
population-postordered 5 : 2 ,  41 ,  -9.80506435788289
population-postordered 6 : 2 ,  42 ,  -9.804892425277657
population-postordered 7 : 2 ,  45 ,  -9.804369557073995
population-postordered 8 : 2 ,  58 ,  -9.802026640074809
population-postordered 9 : 2 ,  159 ,  -9.785441390101083
population-postordered 10 : 2 ,  182 ,  -9.782432944575818
population-postordered 11 : 2 ,  224 ,  -9.77751659809676
population-postordered 12 : 2 ,  286 ,  -9.771616406685204
population-postordered 13 : 2 ,  468 ,  -9.762820975641366
population-postordered 14 : 3 ,  387 ,  -9.753822531261656
population-postordered 15 : 3 ,  45 ,  -9.707773048432884
population-postordered 16 : 6 ,  457 ,  -9.68863297041823
population-postordered 17 : 6 ,  249 ,  -9.672388124205348
population-postordered 18 : 3 ,  262 ,  -9.640869179516123
population-postordered 19 : 3 ,  259 ,  -9.640751543673383
optimized alpha_wolf =  -9.808713154776056
************************
Iter =  6
------------------------
each population member: i =  0
------------------------
each population member: i =  1
------------------------
each population member: i =  2
------------------------
each population member: i =  3
------------------------
each population member: i =  4
------------------------
each population member: i =  5
------------------------
each population member: i =  6
------------------------
each population member: i =  7
------------------------
each population member: i =  8
------------------------
each population member: i =  9
```

```
------------------------
each population member: i =  10
------------------------
each population member: i =  11
------------------------
each population member: i =  12
------------------------
each population member: i =  13
------------------------
each population member: i =  14
------------------------
each population member: i =  15
------------------------
each population member: i =  16
------------------------
each population member: i =  17
------------------------
each population member: i =  18
------------------------
each population member: i =  19
population-preordered 0 : 2 ,  10 ,  -9.808713154776056
population-preordered 1 : 2 ,  35 ,  -9.806064199003348
population-preordered 2 : 2 ,  37 ,  -9.805737779102293
population-preordered 3 : 2 ,  40 ,  -9.805234983898817
population-preordered 4 : 2 ,  41 ,  -9.80506435788289
population-preordered 5 : 2 ,  41 ,  -9.80506435788289
population-preordered 6 : 2 ,  42 ,  -9.804892425277657
population-preordered 7 : 2 ,  45 ,  -9.804369557073995
population-preordered 8 : 2 ,  58 ,  -9.802026640074809
population-preordered 9 : 2 ,  159 ,  -9.785441390101083
population-preordered 10 : 2 ,  172 ,  -9.783712603520826
population-preordered 11 : 2 ,  224 ,  -9.77751659809676
population-preordered 12 : 2 ,  286 ,  -9.771616406685204
population-preordered 13 : 2 ,  468 ,  -9.762820975641366
population-preordered 14 : 3 ,  387 ,  -9.753822531261656
population-preordered 15 : 2 ,  23 ,  -9.80779262819478
population-preordered 16 : 6 ,  457 ,  -9.68863297041823
population-preordered 17 : 6 ,  249 ,  -9.672388124205348
population-preordered 18 : 2 ,  123 ,  -9.790649057095498
population-preordered 19 : 3 ,  259 ,  -9.640751543673383
- - - - - - - - - - - -
population-postordered 0 : 2 ,  10 ,  -9.808713154776056
population-postordered 1 : 2 ,  23 ,  -9.80779262819478
population-postordered 2 : 2 ,  35 ,  -9.806064199003348
population-postordered 3 : 2 ,  37 ,  -9.805737779102293
population-postordered 4 : 2 ,  40 ,  -9.805234983898817
population-postordered 5 : 2 ,  41 ,  -9.80506435788289
population-postordered 6 : 2 ,  41 ,  -9.80506435788289
```

```
population-postordered 7 : 2 ,  42 ,  -9.804892425277657
population-postordered 8 : 2 ,  45 ,  -9.804369557073995
population-postordered 9 : 2 ,  58 ,  -9.802026640074809
population-postordered 10 : 2 ,  123 ,  -9.790649057095498
population-postordered 11 : 2 ,  159 ,  -9.785441390101083
population-postordered 12 : 2 ,  172 ,  -9.783712603520826
population-postordered 13 : 2 ,  224 ,  -9.77751659809676
population-postordered 14 : 2 ,  286 ,  -9.771616406685204
population-postordered 15 : 2 ,  468 ,  -9.762820975641366
population-postordered 16 : 3 ,  387 ,  -9.753822531261656
population-postordered 17 : 6 ,  457 ,  -9.68863297041823
population-postordered 18 : 6 ,  249 ,  -9.672388124205348
population-postordered 19 : 3 ,  259 ,  -9.640751543673383
optimized alpha_wolf =  -9.808713154776056
************************
Iter =  7
------------------------
each population member: i =  0
------------------------
each population member: i =  1
------------------------
each population member: i =  2
------------------------
each population member: i =  3
------------------------
each population member: i =  4
------------------------
each population member: i =  5
------------------------
each population member: i =  6
------------------------
each population member: i =  7
------------------------
each population member: i =  8
------------------------
each population member: i =  9
------------------------
each population member: i =  10
------------------------
each population member: i =  11
------------------------
each population member: i =  12
------------------------
each population member: i =  13
------------------------
each population member: i =  14
------------------------
each population member: i =  15
```

```
------------------------
each population member: i =  16
------------------------
each population member: i =  17
------------------------
each population member: i =  18
------------------------
each population member: i =  19
population-preordered 0 : 2 ,  10 ,  -9.808713154776056
population-preordered 1 : 2 ,  23 ,  -9.80779262819478
population-preordered 2 : 2 ,  35 ,  -9.806064199003348
population-preordered 3 : 2 ,  19 ,  -9.808230325125505
population-preordered 4 : 2 ,  40 ,  -9.805234983898817
population-preordered 5 : 2 ,  35 ,  -9.806064199003348
population-preordered 6 : 2 ,  41 ,  -9.80506435788289
population-preordered 7 : 2 ,  42 ,  -9.804892425277657
population-preordered 8 : 2 ,  45 ,  -9.804369557073995
population-preordered 9 : 2 ,  19 ,  -9.808230325125505
population-preordered 10 : 2 ,  71 ,  -9.79964088972756
population-preordered 11 : 2 ,  121 ,  -9.790962631625304
population-preordered 12 : 2 ,  172 ,  -9.783712603520826
population-preordered 13 : 2 ,  224 ,  -9.77751659809676
population-preordered 14 : 2 ,  286 ,  -9.771616406685204
population-preordered 15 : 2 ,  98 ,  -9.794801203675961
population-preordered 16 : 3 ,  387 ,  -9.753822531261656
population-preordered 17 : 6 ,  457 ,  -9.68863297041823
population-preordered 18 : 6 ,  249 ,  -9.672388124205348
population-preordered 19 : 3 ,  259 ,  -9.640751543673383
- - - - - - - - - - - - -
population-postordered 0 : 2 ,  10 ,  -9.808713154776056
population-postordered 1 : 2 ,  19 ,  -9.808230325125505
population-postordered 2 : 2 ,  19 ,  -9.808230325125505
population-postordered 3 : 2 ,  23 ,  -9.80779262819478
population-postordered 4 : 2 ,  35 ,  -9.806064199003348
population-postordered 5 : 2 ,  35 ,  -9.806064199003348
population-postordered 6 : 2 ,  40 ,  -9.805234983898817
population-postordered 7 : 2 ,  41 ,  -9.80506435788289
population-postordered 8 : 2 ,  42 ,  -9.804892425277657
population-postordered 9 : 2 ,  45 ,  -9.804369557073995
population-postordered 10 : 2 ,  71 ,  -9.79964088972756
population-postordered 11 : 2 ,  98 ,  -9.794801203675961
population-postordered 12 : 2 ,  121 ,  -9.790962631625304
population-postordered 13 : 2 ,  172 ,  -9.783712603520826
population-postordered 14 : 2 ,  224 ,  -9.77751659809676
population-postordered 15 : 2 ,  286 ,  -9.771616406685204
population-postordered 16 : 3 ,  387 ,  -9.753822531261656
population-postordered 17 : 6 ,  457 ,  -9.68863297041823
population-postordered 18 : 6 ,  249 ,  -9.672388124205348
```

```
population-postordered 19 : 3 ,  259 ,  -9.640751543673383
optimized alpha_wolf =  -9.808713154776056
************************
Iter =  8
------------------------
each population member: i =  0
------------------------
each population member: i =  1
------------------------
each population member: i =  2
------------------------
each population member: i =  3
------------------------
each population member: i =  4
------------------------
each population member: i =  5
------------------------
each population member: i =  6
------------------------
each population member: i =  7
------------------------
each population member: i =  8
------------------------
each population member: i =  9
------------------------
each population member: i =  10
------------------------
each population member: i =  11
------------------------
each population member: i =  12
------------------------
each population member: i =  13
------------------------
each population member: i =  14
------------------------
each population member: i =  15
------------------------
each population member: i =  16
------------------------
each population member: i =  17
------------------------
each population member: i =  18
------------------------
each population member: i =  19
population-preordered 0 : 2 ,  10 ,  -9.808713154776056
population-preordered 1 : 2 ,  19 ,  -9.808230325125505
population-preordered 2 : 2 ,  19 ,  -9.808230325125505
population-preordered 3 : 2 ,  23 ,  -9.80779262819478
```

```
population-preordered 4 : 2 ,  25 ,  -9.80754111494395
population-preordered 5 : 2 ,  16 ,  -9.808485178623366
population-preordered 6 : 2 ,  15 ,  -9.808552543638303
population-preordered 7 : 2 ,  41 ,  -9.80506435788289
population-preordered 8 : 2 ,  42 ,  -9.804892425277657
population-preordered 9 : 2 ,  45 ,  -9.804369557073995
population-preordered 10 : 2 ,  71 ,  -9.79964088972756
population-preordered 11 : 2 ,  41 ,  -9.80506435788289
population-preordered 12 : 2 ,  121 ,  -9.790962631625304
population-preordered 13 : 2 ,  172 ,  -9.783712603520826
population-preordered 14 : 2 ,  224 ,  -9.77751659809676
population-preordered 15 : 2 ,  286 ,  -9.771616406685204
population-preordered 16 : 2 ,  16 ,  -9.808485178623366
population-preordered 17 : 6 ,  457 ,  -9.68863297041823
population-preordered 18 : 6 ,  249 ,  -9.672388124205348
population-preordered 19 : 3 ,  259 ,  -9.640751543673383
- - - - - - - - - - - -
population-postordered 0 : 2 ,  10 ,  -9.808713154776056
population-postordered 1 : 2 ,  15 ,  -9.808552543638303
population-postordered 2 : 2 ,  16 ,  -9.808485178623366
population-postordered 3 : 2 ,  16 ,  -9.808485178623366
population-postordered 4 : 2 ,  19 ,  -9.808230325125505
population-postordered 5 : 2 ,  19 ,  -9.808230325125505
population-postordered 6 : 2 ,  23 ,  -9.80779262819478
population-postordered 7 : 2 ,  25 ,  -9.80754111494395
population-postordered 8 : 2 ,  41 ,  -9.80506435788289
population-postordered 9 : 2 ,  41 ,  -9.80506435788289
population-postordered 10 : 2 ,  42 ,  -9.804892425277657
population-postordered 11 : 2 ,  45 ,  -9.804369557073995
population-postordered 12 : 2 ,  71 ,  -9.79964088972756
population-postordered 13 : 2 ,  121 ,  -9.790962631625304
population-postordered 14 : 2 ,  172 ,  -9.783712603520826
population-postordered 15 : 2 ,  224 ,  -9.77751659809676
population-postordered 16 : 2 ,  286 ,  -9.771616406685204
population-postordered 17 : 6 ,  457 ,  -9.68863297041823
population-postordered 18 : 6 ,  249 ,  -9.672388124205348
population-postordered 19 : 3 ,  259 ,  -9.640751543673383
optimized alpha_wolf =  -9.808713154776056
************************
Iter =  9
------------------------
each population member: i =  0
------------------------
each population member: i =  1
------------------------
each population member: i =  2
------------------------
each population member: i =  3
```

```
------------------------
each population member: i =  4
------------------------
each population member: i =  5
------------------------
each population member: i =  6
------------------------
each population member: i =  7
------------------------
each population member: i =  8
------------------------
each population member: i =  9
------------------------
each population member: i =  10
------------------------
each population member: i =  11
------------------------
each population member: i =  12
------------------------
each population member: i =  13
------------------------
each population member: i =  14
------------------------
each population member: i =  15
------------------------
each population member: i =  16
------------------------
each population member: i =  17
------------------------
each population member: i =  18
------------------------
each population member: i =  19
population-preordered 0 : 2 ,  10 ,  -9.808713154776056
population-preordered 1 : 2 ,  15 ,  -9.808552543638303
population-preordered 2 : 2 ,  16 ,  -9.808485178623366
population-preordered 3 : 2 ,  16 ,  -9.808485178623366
population-preordered 4 : 2 ,  19 ,  -9.808230325125505
population-preordered 5 : 2 ,  19 ,  -9.808230325125505
population-preordered 6 : 2 ,  23 ,  -9.80779262819478
population-preordered 7 : 2 ,  25 ,  -9.80754111494395
population-preordered 8 : 2 ,  41 ,  -9.80506435788289
population-preordered 9 : 2 ,  41 ,  -9.80506435788289
population-preordered 10 : 2 ,  42 ,  -9.804892425277657
population-preordered 11 : 2 ,  45 ,  -9.804369557073995
population-preordered 12 : 2 ,  71 ,  -9.79964088972756
population-preordered 13 : 2 ,  22 ,  -9.807910874428904
population-preordered 14 : 2 ,  172 ,  -9.783712603520826
population-preordered 15 : 2 ,  224 ,  -9.77751659809676
```

```
population-preordered 16 : 2 ,  286 ,  -9.771616406685204
population-preordered 17 : 6 ,  457 ,  -9.68863297041823
population-preordered 18 : 6 ,  249 ,  -9.672388124205348
population-preordered 19 : 2 ,  47 ,  -9.804016015787624
- - - - - - - - - - - -
population-postordered 0 : 2 ,  10 ,  -9.808713154776056
population-postordered 1 : 2 ,  15 ,  -9.808552543638303
population-postordered 2 : 2 ,  16 ,  -9.808485178623366
population-postordered 3 : 2 ,  16 ,  -9.808485178623366
population-postordered 4 : 2 ,  19 ,  -9.808230325125505
population-postordered 5 : 2 ,  19 ,  -9.808230325125505
population-postordered 6 : 2 ,  22 ,  -9.807910874428904
population-postordered 7 : 2 ,  23 ,  -9.80779262819478
population-postordered 8 : 2 ,  25 ,  -9.80754111494395
population-postordered 9 : 2 ,  41 ,  -9.80506435788289
population-postordered 10 : 2 ,  41 ,  -9.80506435788289
population-postordered 11 : 2 ,  42 ,  -9.804892425277657
population-postordered 12 : 2 ,  45 ,  -9.804369557073995
population-postordered 13 : 2 ,  47 ,  -9.804016015787624
population-postordered 14 : 2 ,  71 ,  -9.79964088972756
population-postordered 15 : 2 ,  172 ,  -9.783712603520826
population-postordered 16 : 2 ,  224 ,  -9.77751659809676
population-postordered 17 : 2 ,  286 ,  -9.771616406685204
population-postordered 18 : 6 ,  457 ,  -9.68863297041823
population-postordered 19 : 6 ,  249 ,  -9.672388124205348
optimized alpha_wolf =  -9.808713154776056
************************
Iter =  10
------------------------
each population member: i =  0
------------------------
each population member: i =  1
------------------------
each population member: i =  2
------------------------
each population member: i =  3
------------------------
each population member: i =  4
------------------------
each population member: i =  5
------------------------
each population member: i =  6
------------------------
each population member: i =  7
------------------------
each population member: i =  8
------------------------
each population member: i =  9
```

```
------------------------
each population member: i =  10
------------------------
each population member: i =  11
------------------------
each population member: i =  12
------------------------
each population member: i =  13
------------------------
each population member: i =  14
------------------------
each population member: i =  15
------------------------
each population member: i =  16
------------------------
each population member: i =  17
------------------------
each population member: i =  18
------------------------
each population member: i =  19
population-preordered 0 : 2 ,  10 ,  -9.808713154776056
population-preordered 1 : 2 ,  15 ,  -9.808552543638303
population-preordered 2 : 2 ,  15 ,  -9.808552543638303
population-preordered 3 : 2 ,  15 ,  -9.808552543638303
population-preordered 4 : 2 ,  19 ,  -9.808230325125505
population-preordered 5 : 2 ,  19 ,  -9.808230325125505
population-preordered 6 : 2 ,  22 ,  -9.807910874428904
population-preordered 7 : 2 ,  23 ,  -9.80779262819478
population-preordered 8 : 2 ,  25 ,  -9.80754111494395
population-preordered 9 : 2 ,  41 ,  -9.80506435788289
population-preordered 10 : 2 ,  41 ,  -9.80506435788289
population-preordered 11 : 2 ,  42 ,  -9.804892425277657
population-preordered 12 : 2 ,  45 ,  -9.804369557073995
population-preordered 13 : 2 ,  10 ,  -9.808713154776056
population-preordered 14 : 2 ,  71 ,  -9.79964088972756
population-preordered 15 : 2 ,  172 ,  -9.783712603520826
population-preordered 16 : 2 ,  72 ,  -9.799457752354563
population-preordered 17 : 2 ,  286 ,  -9.771616406685204
population-preordered 18 : 6 ,  457 ,  -9.68863297041823
population-preordered 19 : 6 ,  249 ,  -9.672388124205348
- - - - - - - - - - - -
population-postordered 0 : 2 ,  10 ,  -9.808713154776056
population-postordered 1 : 2 ,  10 ,  -9.808713154776056
population-postordered 2 : 2 ,  15 ,  -9.808552543638303
population-postordered 3 : 2 ,  15 ,  -9.808552543638303
population-postordered 4 : 2 ,  15 ,  -9.808552543638303
population-postordered 5 : 2 ,  19 ,  -9.808230325125505
population-postordered 6 : 2 ,  19 ,  -9.808230325125505
```

```
population-postordered 7 : 2 ,  22 ,  -9.807910874428904
population-postordered 8 : 2 ,  23 ,  -9.80779262819478
population-postordered 9 : 2 ,  25 ,  -9.80754111494395
population-postordered 10 : 2 ,  41 ,  -9.80506435788289
population-postordered 11 : 2 ,  41 ,  -9.80506435788289
population-postordered 12 : 2 ,  42 ,  -9.804892425277657
population-postordered 13 : 2 ,  45 ,  -9.804369557073995
population-postordered 14 : 2 ,  71 ,  -9.79964088972756
population-postordered 15 : 2 ,  72 ,  -9.799457752354563
population-postordered 16 : 2 ,  172 ,  -9.783712603520826
population-postordered 17 : 2 ,  286 ,  -9.771616406685204
population-postordered 18 : 6 ,  457 ,  -9.68863297041823
population-postordered 19 : 6 ,  249 ,  -9.672388124205348
optimized alpha_wolf =  -9.808713154776056
************************
Iter =  11
------------------------
each population member: i =  0
------------------------
each population member: i =  1
------------------------
each population member: i =  2
------------------------
each population member: i =  3
------------------------
each population member: i =  4
------------------------
each population member: i =  5
------------------------
each population member: i =  6
------------------------
each population member: i =  7
------------------------
each population member: i =  8
------------------------
each population member: i =  9
------------------------
each population member: i =  10
------------------------
each population member: i =  11
------------------------
each population member: i =  12
------------------------
each population member: i =  13
------------------------
each population member: i =  14
------------------------
each population member: i =  15
```

```
------------------------
each population member: i =  16
------------------------
each population member: i =  17
------------------------
each population member: i =  18
------------------------
each population member: i =  19
population-preordered 0 : 2 ,  10 ,  -9.808713154776056
population-preordered 1 : 2 ,  10 ,  -9.808713154776056
population-preordered 2 : 2 ,  11 ,  -9.808708701829195
population-preordered 3 : 2 ,  15 ,  -9.808552543638303
population-preordered 4 : 2 ,  15 ,  -9.808552543638303
population-preordered 5 : 2 ,  19 ,  -9.808230325125505
population-preordered 6 : 2 ,  19 ,  -9.808230325125505
population-preordered 7 : 2 ,  22 ,  -9.807910874428904
population-preordered 8 : 2 ,  23 ,  -9.80779262819478
population-preordered 9 : 2 ,  25 ,  -9.80754111494395
population-preordered 10 : 2 ,  41 ,  -9.80506435788289
population-preordered 11 : 2 ,  41 ,  -9.80506435788289
population-preordered 12 : 2 ,  42 ,  -9.804892425277657
population-preordered 13 : 2 ,  45 ,  -9.804369557073995
population-preordered 14 : 2 ,  71 ,  -9.79964088972756
population-preordered 15 : 2 ,  72 ,  -9.799457752354563
population-preordered 16 : 2 ,  172 ,  -9.783712603520826
population-preordered 17 : 2 ,  286 ,  -9.771616406685204
population-preordered 18 : 2 ,  225 ,  -9.777408395843187
population-preordered 19 : 6 ,  249 ,  -9.672388124205348
- - - - - - - - - - - -
population-postordered 0 : 2 ,  10 ,  -9.808713154776056
population-postordered 1 : 2 ,  10 ,  -9.808713154776056
population-postordered 2 : 2 ,  11 ,  -9.808708701829195
population-postordered 3 : 2 ,  15 ,  -9.808552543638303
population-postordered 4 : 2 ,  15 ,  -9.808552543638303
population-postordered 5 : 2 ,  19 ,  -9.808230325125505
population-postordered 6 : 2 ,  19 ,  -9.808230325125505
population-postordered 7 : 2 ,  22 ,  -9.807910874428904
population-postordered 8 : 2 ,  23 ,  -9.80779262819478
population-postordered 9 : 2 ,  25 ,  -9.80754111494395
population-postordered 10 : 2 ,  41 ,  -9.80506435788289
population-postordered 11 : 2 ,  41 ,  -9.80506435788289
population-postordered 12 : 2 ,  42 ,  -9.804892425277657
population-postordered 13 : 2 ,  45 ,  -9.804369557073995
population-postordered 14 : 2 ,  71 ,  -9.79964088972756
population-postordered 15 : 2 ,  72 ,  -9.799457752354563
population-postordered 16 : 2 ,  172 ,  -9.783712603520826
population-postordered 17 : 2 ,  225 ,  -9.777408395843187
population-postordered 18 : 2 ,  286 ,  -9.771616406685204
```

```
population-postordered 19 : 6 ,  249 ,  -9.672388124205348
optimized alpha_wolf =  -9.808713154776056
************************
Iter =  12
------------------------
each population member: i =  0
------------------------
each population member: i =  1
------------------------
each population member: i =  2
------------------------
each population member: i =  3
------------------------
each population member: i =  4
------------------------
each population member: i =  5
------------------------
each population member: i =  6
------------------------
each population member: i =  7
------------------------
each population member: i =  8
------------------------
each population member: i =  9
------------------------
each population member: i =  10
------------------------
each population member: i =  11
------------------------
each population member: i =  12
------------------------
each population member: i =  13
------------------------
each population member: i =  14
------------------------
each population member: i =  15
------------------------
each population member: i =  16
------------------------
each population member: i =  17
------------------------
each population member: i =  18
------------------------
each population member: i =  19
population-preordered 0 : 2 ,  10 ,  -9.808713154776056
population-preordered 1 : 2 ,  10 ,  -9.808713154776056
population-preordered 2 : 2 ,  11 ,  -9.808708701829195
population-preordered 3 : 2 ,  15 ,  -9.808552543638303
```

```
population-preordered 4 : 2 ,  15 ,  -9.808552543638303
population-preordered 5 : 2 ,  19 ,  -9.808230325125505
population-preordered 6 : 2 ,  8 ,  -9.808669655892167
population-preordered 7 : 2 ,  22 ,  -9.807910874428904
population-preordered 8 : 2 ,  23 ,  -9.80779262819478
population-preordered 9 : 2 ,  25 ,  -9.80754111494395
population-preordered 10 : 2 ,  21 ,  -9.808023562642509
population-preordered 11 : 2 ,  41 ,  -9.80506435788289
population-preordered 12 : 2 ,  42 ,  -9.804892425277657
population-preordered 13 : 2 ,  45 ,  -9.804369557073995
population-preordered 14 : 2 ,  71 ,  -9.79964088972756
population-preordered 15 : 2 ,  72 ,  -9.799457752354563
population-preordered 16 : 2 ,  72 ,  -9.799457752354563
population-preordered 17 : 2 ,  225 ,  -9.777408395843187
population-preordered 18 : 2 ,  286 ,  -9.771616406685204
population-preordered 19 : 6 ,  249 ,  -9.672388124205348
- - - - - - - - - - - -
population-postordered 0 : 2 ,  10 ,  -9.808713154776056
population-postordered 1 : 2 ,  10 ,  -9.808713154776056
population-postordered 2 : 2 ,  11 ,  -9.808708701829195
population-postordered 3 : 2 ,  8 ,  -9.808669655892167
population-postordered 4 : 2 ,  15 ,  -9.808552543638303
population-postordered 5 : 2 ,  15 ,  -9.808552543638303
population-postordered 6 : 2 ,  19 ,  -9.808230325125505
population-postordered 7 : 2 ,  21 ,  -9.808023562642509
population-postordered 8 : 2 ,  22 ,  -9.807910874428904
population-postordered 9 : 2 ,  23 ,  -9.80779262819478
population-postordered 10 : 2 ,  25 ,  -9.80754111494395
population-postordered 11 : 2 ,  41 ,  -9.80506435788289
population-postordered 12 : 2 ,  42 ,  -9.804892425277657
population-postordered 13 : 2 ,  45 ,  -9.804369557073995
population-postordered 14 : 2 ,  71 ,  -9.79964088972756
population-postordered 15 : 2 ,  72 ,  -9.799457752354563
population-postordered 16 : 2 ,  72 ,  -9.799457752354563
population-postordered 17 : 2 ,  225 ,  -9.777408395843187
population-postordered 18 : 2 ,  286 ,  -9.771616406685204
population-postordered 19 : 6 ,  249 ,  -9.672388124205348
optimized alpha_wolf =  -9.808713154776056
************************
Iter =  13
------------------------
each population member: i =  0
------------------------
each population member: i =  1
------------------------
each population member: i =  2
------------------------
each population member: i =  3
```

```
------------------------
each population member: i =  4
------------------------
each population member: i =  5
------------------------
each population member: i =  6
------------------------
each population member: i =  7
------------------------
each population member: i =  8
------------------------
each population member: i =  9
------------------------
each population member: i =  10
------------------------
each population member: i =  11
------------------------
each population member: i =  12
------------------------
each population member: i =  13
------------------------
each population member: i =  14
------------------------
each population member: i =  15
------------------------
each population member: i =  16
------------------------
each population member: i =  17
------------------------
each population member: i =  18
------------------------
each population member: i =  19
population-preordered 0 : 2 ,  10 ,  -9.808713154776056
population-preordered 1 : 2 ,  10 ,  -9.808713154776056
population-preordered 2 : 2 ,  11 ,  -9.808708701829195
population-preordered 3 : 2 ,  8 ,  -9.808669655892167
population-preordered 4 : 2 ,  8 ,  -9.808669655892167
population-preordered 5 : 2 ,  15 ,  -9.808552543638303
population-preordered 6 : 2 ,  19 ,  -9.808230325125505
population-preordered 7 : 2 ,  21 ,  -9.808023562642509
population-preordered 8 : 2 ,  22 ,  -9.807910874428904
population-preordered 9 : 2 ,  10 ,  -9.808713154776056
population-preordered 10 : 2 ,  25 ,  -9.80754111494395
population-preordered 11 : 2 ,  41 ,  -9.80506435788289
population-preordered 12 : 2 ,  42 ,  -9.804892425277657
population-preordered 13 : 2 ,  45 ,  -9.804369557073995
population-preordered 14 : 2 ,  71 ,  -9.79964088972756
population-preordered 15 : 2 ,  72 ,  -9.799457752354563
```

```
population-preordered 16 : 2 ,  72 ,  -9.799457752354563
population-preordered 17 : 2 ,  225 ,  -9.777408395843187
population-preordered 18 : 2 ,  286 ,  -9.771616406685204
population-preordered 19 : 6 ,  249 ,  -9.672388124205348
- - - - - - - - - - - -
population-postordered 0 : 2 ,  10 ,  -9.808713154776056
population-postordered 1 : 2 ,  10 ,  -9.808713154776056
population-postordered 2 : 2 ,  10 ,  -9.808713154776056
population-postordered 3 : 2 ,  11 ,  -9.808708701829195
population-postordered 4 : 2 ,  8 ,  -9.808669655892167
population-postordered 5 : 2 ,  8 ,  -9.808669655892167
population-postordered 6 : 2 ,  15 ,  -9.808552543638303
population-postordered 7 : 2 ,  19 ,  -9.808230325125505
population-postordered 8 : 2 ,  21 ,  -9.808023562642509
population-postordered 9 : 2 ,  22 ,  -9.807910874428904
population-postordered 10 : 2 ,  25 ,  -9.80754111494395
population-postordered 11 : 2 ,  41 ,  -9.80506435788289
population-postordered 12 : 2 ,  42 ,  -9.804892425277657
population-postordered 13 : 2 ,  45 ,  -9.804369557073995
population-postordered 14 : 2 ,  71 ,  -9.79964088972756
population-postordered 15 : 2 ,  72 ,  -9.799457752354563
population-postordered 16 : 2 ,  72 ,  -9.799457752354563
population-postordered 17 : 2 ,  225 ,  -9.777408395843187
population-postordered 18 : 2 ,  286 ,  -9.771616406685204
population-postordered 19 : 6 ,  249 ,  -9.672388124205348
optimized alpha_wolf =  -9.808713154776056
************************
Iter =  14
------------------------
each population member: i =  0
------------------------
each population member: i =  1
------------------------
each population member: i =  2
------------------------
each population member: i =  3
------------------------
each population member: i =  4
------------------------
each population member: i =  5
------------------------
each population member: i =  6
------------------------
each population member: i =  7
------------------------
each population member: i =  8
------------------------
each population member: i =  9
```

```
------------------------
each population member: i =  10
------------------------
each population member: i =  11
------------------------
each population member: i =  12
------------------------
each population member: i =  13
------------------------
each population member: i =  14
------------------------
each population member: i =  15
------------------------
each population member: i =  16
------------------------
each population member: i =  17
------------------------
each population member: i =  18
------------------------
each population member: i =  19
population-preordered 0 : 2 ,  10 ,  -9.808713154776056
population-preordered 1 : 2 ,  10 ,  -9.808713154776056
population-preordered 2 : 2 ,  10 ,  -9.808713154776056
population-preordered 3 : 2 ,  11 ,  -9.808708701829195
population-preordered 4 : 2 ,  8 ,  -9.808669655892167
population-preordered 5 : 2 ,  8 ,  -9.808669655892167
population-preordered 6 : 2 ,  15 ,  -9.808552543638303
population-preordered 7 : 2 ,  19 ,  -9.808230325125505
population-preordered 8 : 2 ,  7 ,  -9.80861820158067
population-preordered 9 : 2 ,  22 ,  -9.807910874428904
population-preordered 10 : 2 ,  7 ,  -9.80861820158067
population-preordered 11 : 2 ,  41 ,  -9.80506435788289
population-preordered 12 : 2 ,  9 ,  -9.808700730578108
population-preordered 13 : 2 ,  11 ,  -9.808708701829195
population-preordered 14 : 2 ,  71 ,  -9.79964088972756
population-preordered 15 : 2 ,  72 ,  -9.799457752354563
population-preordered 16 : 2 ,  72 ,  -9.799457752354563
population-preordered 17 : 2 ,  225 ,  -9.777408395843187
population-preordered 18 : 2 ,  286 ,  -9.771616406685204
population-preordered 19 : 6 ,  249 ,  -9.672388124205348
- - - - - - - - - - - -
population-postordered 0 : 2 ,  10 ,  -9.808713154776056
population-postordered 1 : 2 ,  10 ,  -9.808713154776056
population-postordered 2 : 2 ,  10 ,  -9.808713154776056
population-postordered 3 : 2 ,  11 ,  -9.808708701829195
population-postordered 4 : 2 ,  11 ,  -9.808708701829195
population-postordered 5 : 2 ,  9 ,  -9.808700730578108
population-postordered 6 : 2 ,  8 ,  -9.808669655892167
```

```
population-postordered 7 : 2 ,  8 ,  -9.808669655892167
population-postordered 8 : 2 ,  7 ,  -9.80861820158067
population-postordered 9 : 2 ,  7 ,  -9.80861820158067
population-postordered 10 : 2 ,  15 ,  -9.808552543638303
population-postordered 11 : 2 ,  19 ,  -9.808230325125505
population-postordered 12 : 2 ,  22 ,  -9.807910874428904
population-postordered 13 : 2 ,  41 ,  -9.80506435788289
population-postordered 14 : 2 ,  71 ,  -9.79964088972756
population-postordered 15 : 2 ,  72 ,  -9.799457752354563
population-postordered 16 : 2 ,  72 ,  -9.799457752354563
population-postordered 17 : 2 ,  225 ,  -9.777408395843187
population-postordered 18 : 2 ,  286 ,  -9.771616406685204
population-postordered 19 : 6 ,  249 ,  -9.672388124205348
optimized alpha_wolf =  -9.808713154776056
************************
Iter =  15
------------------------
each population member: i =  0
------------------------
each population member: i =  1
------------------------
each population member: i =  2
------------------------
each population member: i =  3
------------------------
each population member: i =  4
------------------------
each population member: i =  5
------------------------
each population member: i =  6
------------------------
each population member: i =  7
------------------------
each population member: i =  8
------------------------
each population member: i =  9
------------------------
each population member: i =  10
------------------------
each population member: i =  11
------------------------
each population member: i =  12
------------------------
each population member: i =  13
------------------------
each population member: i =  14
------------------------
each population member: i =  15
```

```
------------------------
each population member: i =  16
------------------------
each population member: i =  17
------------------------
each population member: i =  18
------------------------
each population member: i =  19
population-preordered 0 : 2 ,  10 ,  -9.808713154776056
population-preordered 1 : 2 ,  10 ,  -9.808713154776056
population-preordered 2 : 2 ,  10 ,  -9.808713154776056
population-preordered 3 : 2 ,  11 ,  -9.808708701829195
population-preordered 4 : 2 ,  11 ,  -9.808708701829195
population-preordered 5 : 2 ,  9 ,  -9.808700730578108
population-preordered 6 : 2 ,  8 ,  -9.808669655892167
population-preordered 7 : 2 ,  8 ,  -9.808669655892167
population-preordered 8 : 2 ,  7 ,  -9.80861820158067
population-preordered 9 : 2 ,  7 ,  -9.80861820158067
population-preordered 10 : 2 ,  15 ,  -9.808552543638303
population-preordered 11 : 2 ,  19 ,  -9.808230325125505
population-preordered 12 : 2 ,  22 ,  -9.807910874428904
population-preordered 13 : 2 ,  41 ,  -9.80506435788289
population-preordered 14 : 2 ,  71 ,  -9.79964088972756
population-preordered 15 : 2 ,  72 ,  -9.799457752354563
population-preordered 16 : 2 ,  72 ,  -9.799457752354563
population-preordered 17 : 2 ,  225 ,  -9.777408395843187
population-preordered 18 : 2 ,  286 ,  -9.771616406685204
population-preordered 19 : 3 ,  22 ,  -9.755709878840403
- - - - - - - - - - - -
population-postordered 0 : 2 ,  10 ,  -9.808713154776056
population-postordered 1 : 2 ,  10 ,  -9.808713154776056
population-postordered 2 : 2 ,  10 ,  -9.808713154776056
population-postordered 3 : 2 ,  11 ,  -9.808708701829195
population-postordered 4 : 2 ,  11 ,  -9.808708701829195
population-postordered 5 : 2 ,  9 ,  -9.808700730578108
population-postordered 6 : 2 ,  8 ,  -9.808669655892167
population-postordered 7 : 2 ,  8 ,  -9.808669655892167
population-postordered 8 : 2 ,  7 ,  -9.80861820158067
population-postordered 9 : 2 ,  7 ,  -9.80861820158067
population-postordered 10 : 2 ,  15 ,  -9.808552543638303
population-postordered 11 : 2 ,  19 ,  -9.808230325125505
population-postordered 12 : 2 ,  22 ,  -9.807910874428904
population-postordered 13 : 2 ,  41 ,  -9.80506435788289
population-postordered 14 : 2 ,  71 ,  -9.79964088972756
population-postordered 15 : 2 ,  72 ,  -9.799457752354563
population-postordered 16 : 2 ,  72 ,  -9.799457752354563
population-postordered 17 : 2 ,  225 ,  -9.777408395843187
population-postordered 18 : 2 ,  286 ,  -9.771616406685204
```

```
population-postordered 19 : 3 ,  22 ,  -9.755709878840403
optimized alpha_wolf =  -9.808713154776056
************************
Iter =  16
------------------------
each population member: i =  0
------------------------
each population member: i =  1
------------------------
each population member: i =  2
------------------------
each population member: i =  3
------------------------
each population member: i =  4
------------------------
each population member: i =  5
------------------------
each population member: i =  6
------------------------
each population member: i =  7
------------------------
each population member: i =  8
------------------------
each population member: i =  9
------------------------
each population member: i =  10
------------------------
each population member: i =  11
------------------------
each population member: i =  12
------------------------
each population member: i =  13
------------------------
each population member: i =  14
------------------------
each population member: i =  15
------------------------
each population member: i =  16
------------------------
each population member: i =  17
------------------------
each population member: i =  18
------------------------
each population member: i =  19
population-preordered 0 : 2 ,  10 ,  -9.808713154776056
population-preordered 1 : 2 ,  10 ,  -9.808713154776056
population-preordered 2 : 2 ,  10 ,  -9.808713154776056
population-preordered 3 : 2 ,  11 ,  -9.808708701829195
```

```
population-preordered 4 : 2 ,  10 ,  -9.808713154776056
population-preordered 5 : 2 ,  9 ,  -9.808700730578108
population-preordered 6 : 2 ,  8 ,  -9.808669655892167
population-preordered 7 : 2 ,  8 ,  -9.808669655892167
population-preordered 8 : 2 ,  7 ,  -9.80861820158067
population-preordered 9 : 2 ,  7 ,  -9.80861820158067
population-preordered 10 : 2 ,  15 ,  -9.808552543638303
population-preordered 11 : 2 ,  19 ,  -9.808230325125505
population-preordered 12 : 2 ,  22 ,  -9.807910874428904
population-preordered 13 : 2 ,  41 ,  -9.80506435788289
population-preordered 14 : 2 ,  71 ,  -9.79964088972756
population-preordered 15 : 2 ,  72 ,  -9.799457752354563
population-preordered 16 : 2 ,  10 ,  -9.808713154776056
population-preordered 17 : 2 ,  225 ,  -9.777408395843187
population-preordered 18 : 2 ,  286 ,  -9.771616406685204
population-preordered 19 : 3 ,  22 ,  -9.755709878840403
- - - - - - - - - - - -
population-postordered 0 : 2 ,  10 ,  -9.808713154776056
population-postordered 1 : 2 ,  10 ,  -9.808713154776056
population-postordered 2 : 2 ,  10 ,  -9.808713154776056
population-postordered 3 : 2 ,  10 ,  -9.808713154776056
population-postordered 4 : 2 ,  10 ,  -9.808713154776056
population-postordered 5 : 2 ,  11 ,  -9.808708701829195
population-postordered 6 : 2 ,  9 ,  -9.808700730578108
population-postordered 7 : 2 ,  8 ,  -9.808669655892167
population-postordered 8 : 2 ,  8 ,  -9.808669655892167
population-postordered 9 : 2 ,  7 ,  -9.80861820158067
population-postordered 10 : 2 ,  7 ,  -9.80861820158067
population-postordered 11 : 2 ,  15 ,  -9.808552543638303
population-postordered 12 : 2 ,  19 ,  -9.808230325125505
population-postordered 13 : 2 ,  22 ,  -9.807910874428904
population-postordered 14 : 2 ,  41 ,  -9.80506435788289
population-postordered 15 : 2 ,  71 ,  -9.79964088972756
population-postordered 16 : 2 ,  72 ,  -9.799457752354563
population-postordered 17 : 2 ,  225 ,  -9.777408395843187
population-postordered 18 : 2 ,  286 ,  -9.771616406685204
population-postordered 19 : 3 ,  22 ,  -9.755709878840403
optimized alpha_wolf =  -9.808713154776056
************************
Iter =  17
------------------------
each population member: i =  0
------------------------
each population member: i =  1
------------------------
each population member: i =  2
------------------------
each population member: i =  3
```

```
------------------------
each population member: i =  4
------------------------
each population member: i =  5
------------------------
each population member: i =  6
------------------------
each population member: i =  7
------------------------
each population member: i =  8
------------------------
each population member: i =  9
------------------------
each population member: i =  10
------------------------
each population member: i =  11
------------------------
each population member: i =  12
------------------------
each population member: i =  13
------------------------
each population member: i =  14
------------------------
each population member: i =  15
------------------------
each population member: i =  16
------------------------
each population member: i =  17
------------------------
each population member: i =  18
------------------------
each population member: i =  19
population-preordered 0 : 2 ,  10 ,  -9.808713154776056
population-preordered 1 : 2 ,  10 ,  -9.808713154776056
population-preordered 2 : 2 ,  10 ,  -9.808713154776056
population-preordered 3 : 2 ,  10 ,  -9.808713154776056
population-preordered 4 : 2 ,  10 ,  -9.808713154776056
population-preordered 5 : 2 ,  11 ,  -9.808708701829195
population-preordered 6 : 2 ,  9 ,  -9.808700730578108
population-preordered 7 : 2 ,  8 ,  -9.808669655892167
population-preordered 8 : 2 ,  8 ,  -9.808669655892167
population-preordered 9 : 2 ,  7 ,  -9.80861820158067
population-preordered 10 : 2 ,  7 ,  -9.80861820158067
population-preordered 11 : 2 ,  15 ,  -9.808552543638303
population-preordered 12 : 2 ,  19 ,  -9.808230325125505
population-preordered 13 : 2 ,  22 ,  -9.807910874428904
population-preordered 14 : 2 ,  41 ,  -9.80506435788289
population-preordered 15 : 2 ,  71 ,  -9.79964088972756
```

```
population-preordered 16 : 2 ,  72 ,  -9.799457752354563
population-preordered 17 : 2 ,  46 ,  -9.804193229091599
population-preordered 18 : 2 ,  286 ,  -9.771616406685204
population-preordered 19 : 2 ,  10 ,  -9.808713154776056
- - - - - - - - - - - -
population-postordered 0 : 2 ,  10 ,  -9.808713154776056
population-postordered 1 : 2 ,  10 ,  -9.808713154776056
population-postordered 2 : 2 ,  10 ,  -9.808713154776056
population-postordered 3 : 2 ,  10 ,  -9.808713154776056
population-postordered 4 : 2 ,  10 ,  -9.808713154776056
population-postordered 5 : 2 ,  10 ,  -9.808713154776056
population-postordered 6 : 2 ,  11 ,  -9.808708701829195
population-postordered 7 : 2 ,  9 ,  -9.808700730578108
population-postordered 8 : 2 ,  8 ,  -9.808669655892167
population-postordered 9 : 2 ,  8 ,  -9.808669655892167
population-postordered 10 : 2 ,  7 ,  -9.80861820158067
population-postordered 11 : 2 ,  7 ,  -9.80861820158067
population-postordered 12 : 2 ,  15 ,  -9.808552543638303
population-postordered 13 : 2 ,  19 ,  -9.808230325125505
population-postordered 14 : 2 ,  22 ,  -9.807910874428904
population-postordered 15 : 2 ,  41 ,  -9.80506435788289
population-postordered 16 : 2 ,  46 ,  -9.804193229091599
population-postordered 17 : 2 ,  71 ,  -9.79964088972756
population-postordered 18 : 2 ,  72 ,  -9.799457752354563
population-postordered 19 : 2 ,  286 ,  -9.771616406685204
optimized alpha_wolf =  -9.808713154776056
************************
Iter =  18
------------------------
each population member: i =  0
------------------------
each population member: i =  1
------------------------
each population member: i =  2
------------------------
each population member: i =  3
------------------------
each population member: i =  4
------------------------
each population member: i =  5
------------------------
each population member: i =  6
------------------------
each population member: i =  7
------------------------
each population member: i =  8
------------------------
each population member: i =  9
```

```
------------------------
each population member: i =  10
------------------------
each population member: i =  11
------------------------
each population member: i =  12
------------------------
each population member: i =  13
------------------------
each population member: i =  14
------------------------
each population member: i =  15
------------------------
each population member: i =  16
------------------------
each population member: i =  17
------------------------
each population member: i =  18
------------------------
each population member: i =  19
population-preordered 0 : 2 ,  10 ,  -9.808713154776056
population-preordered 1 : 2 ,  10 ,  -9.808713154776056
population-preordered 2 : 2 ,  10 ,  -9.808713154776056
population-preordered 3 : 2 ,  10 ,  -9.808713154776056
population-preordered 4 : 2 ,  10 ,  -9.808713154776056
population-preordered 5 : 2 ,  10 ,  -9.808713154776056
population-preordered 6 : 2 ,  11 ,  -9.808708701829195
population-preordered 7 : 2 ,  9 ,  -9.808700730578108
population-preordered 8 : 2 ,  8 ,  -9.808669655892167
population-preordered 9 : 2 ,  8 ,  -9.808669655892167
population-preordered 10 : 2 ,  7 ,  -9.80861820158067
population-preordered 11 : 2 ,  7 ,  -9.80861820158067
population-preordered 12 : 2 ,  15 ,  -9.808552543638303
population-preordered 13 : 2 ,  19 ,  -9.808230325125505
population-preordered 14 : 2 ,  8 ,  -9.808669655892167
population-preordered 15 : 2 ,  41 ,  -9.80506435788289
population-preordered 16 : 2 ,  46 ,  -9.804193229091599
population-preordered 17 : 2 ,  71 ,  -9.79964088972756
population-preordered 18 : 2 ,  72 ,  -9.799457752354563
population-preordered 19 : 2 ,  286 ,  -9.771616406685204
- - - - - - - - - - - -
population-postordered 0 : 2 ,  10 ,  -9.808713154776056
population-postordered 1 : 2 ,  10 ,  -9.808713154776056
population-postordered 2 : 2 ,  10 ,  -9.808713154776056
population-postordered 3 : 2 ,  10 ,  -9.808713154776056
population-postordered 4 : 2 ,  10 ,  -9.808713154776056
population-postordered 5 : 2 ,  10 ,  -9.808713154776056
population-postordered 6 : 2 ,  11 ,  -9.808708701829195
```

```
population-postordered 7 : 2 ,  9 ,  -9.808700730578108
population-postordered 8 : 2 ,  8 ,  -9.808669655892167
population-postordered 9 : 2 ,  8 ,  -9.808669655892167
population-postordered 10 : 2 ,  8 ,  -9.808669655892167
population-postordered 11 : 2 ,  7 ,  -9.80861820158067
population-postordered 12 : 2 ,  7 ,  -9.80861820158067
population-postordered 13 : 2 ,  15 ,  -9.808552543638303
population-postordered 14 : 2 ,  19 ,  -9.808230325125505
population-postordered 15 : 2 ,  41 ,  -9.80506435788289
population-postordered 16 : 2 ,  46 ,  -9.804193229091599
population-postordered 17 : 2 ,  71 ,  -9.79964088972756
population-postordered 18 : 2 ,  72 ,  -9.799457752354563
population-postordered 19 : 2 ,  286 ,  -9.771616406685204
optimized alpha_wolf =  -9.808713154776056
************************
Iter =  19
------------------------
each population member: i =  0
------------------------
each population member: i =  1
------------------------
each population member: i =  2
------------------------
each population member: i =  3
------------------------
each population member: i =  4
------------------------
each population member: i =  5
------------------------
each population member: i =  6
------------------------
each population member: i =  7
------------------------
each population member: i =  8
------------------------
each population member: i =  9
------------------------
each population member: i =  10
------------------------
each population member: i =  11
------------------------
each population member: i =  12
------------------------
each population member: i =  13
------------------------
each population member: i =  14
------------------------
each population member: i =  15
```

```
------------------------
each population member: i =  16
------------------------
each population member: i =  17
------------------------
each population member: i =  18
------------------------
each population member: i =  19
population-preordered 0 : 2 ,  10 ,  -9.808713154776056
population-preordered 1 : 2 ,  10 ,  -9.808713154776056
population-preordered 2 : 2 ,  10 ,  -9.808713154776056
population-preordered 3 : 2 ,  10 ,  -9.808713154776056
population-preordered 4 : 2 ,  10 ,  -9.808713154776056
population-preordered 5 : 2 ,  10 ,  -9.808713154776056
population-preordered 6 : 2 ,  11 ,  -9.808708701829195
population-preordered 7 : 2 ,  9 ,  -9.808700730578108
population-preordered 8 : 2 ,  8 ,  -9.808669655892167
population-preordered 9 : 2 ,  9 ,  -9.808700730578108
population-preordered 10 : 2 ,  8 ,  -9.808669655892167
population-preordered 11 : 2 ,  7 ,  -9.80861820158067
population-preordered 12 : 2 ,  7 ,  -9.80861820158067
population-preordered 13 : 2 ,  15 ,  -9.808552543638303
population-preordered 14 : 2 ,  19 ,  -9.808230325125505
population-preordered 15 : 2 ,  41 ,  -9.80506435788289
population-preordered 16 : 2 ,  46 ,  -9.804193229091599
population-preordered 17 : 2 ,  71 ,  -9.79964088972756
population-preordered 18 : 2 ,  72 ,  -9.799457752354563
population-preordered 19 : 2 ,  286 ,  -9.771616406685204
- - - - - - - - - - - -
population-postordered 0 : 2 ,  10 ,  -9.808713154776056
population-postordered 1 : 2 ,  10 ,  -9.808713154776056
population-postordered 2 : 2 ,  10 ,  -9.808713154776056
population-postordered 3 : 2 ,  10 ,  -9.808713154776056
population-postordered 4 : 2 ,  10 ,  -9.808713154776056
population-postordered 5 : 2 ,  10 ,  -9.808713154776056
population-postordered 6 : 2 ,  11 ,  -9.808708701829195
population-postordered 7 : 2 ,  9 ,  -9.808700730578108
population-postordered 8 : 2 ,  9 ,  -9.808700730578108
population-postordered 9 : 2 ,  8 ,  -9.808669655892167
population-postordered 10 : 2 ,  8 ,  -9.808669655892167
population-postordered 11 : 2 ,  7 ,  -9.80861820158067
population-postordered 12 : 2 ,  7 ,  -9.80861820158067
population-postordered 13 : 2 ,  15 ,  -9.808552543638303
population-postordered 14 : 2 ,  19 ,  -9.808230325125505
population-postordered 15 : 2 ,  41 ,  -9.80506435788289
population-postordered 16 : 2 ,  46 ,  -9.804193229091599
population-postordered 17 : 2 ,  71 ,  -9.79964088972756
population-postordered 18 : 2 ,  72 ,  -9.799457752354563
```

```
population-postordered 19 : 2 ,  286 ,  -9.771616406685204
optimized alpha_wolf =  -9.808713154776056

GWO completed


Best solution found:

Best solution 1: 2

Best solution 2: 10
fitness of best solution =: -9.808713154776056

End GWO
```

8 # Final

[34]:
```
#signal=eeg8[0:511]

signal=eeg_1024
#signal=f1

alpha = 100
tau = 0
DC = 0
init = 0
tol = 1e-7
K = 2
u,u_t,u_hat,omega = VMD(signal, alpha, tau, K, DC, init, tol)
```

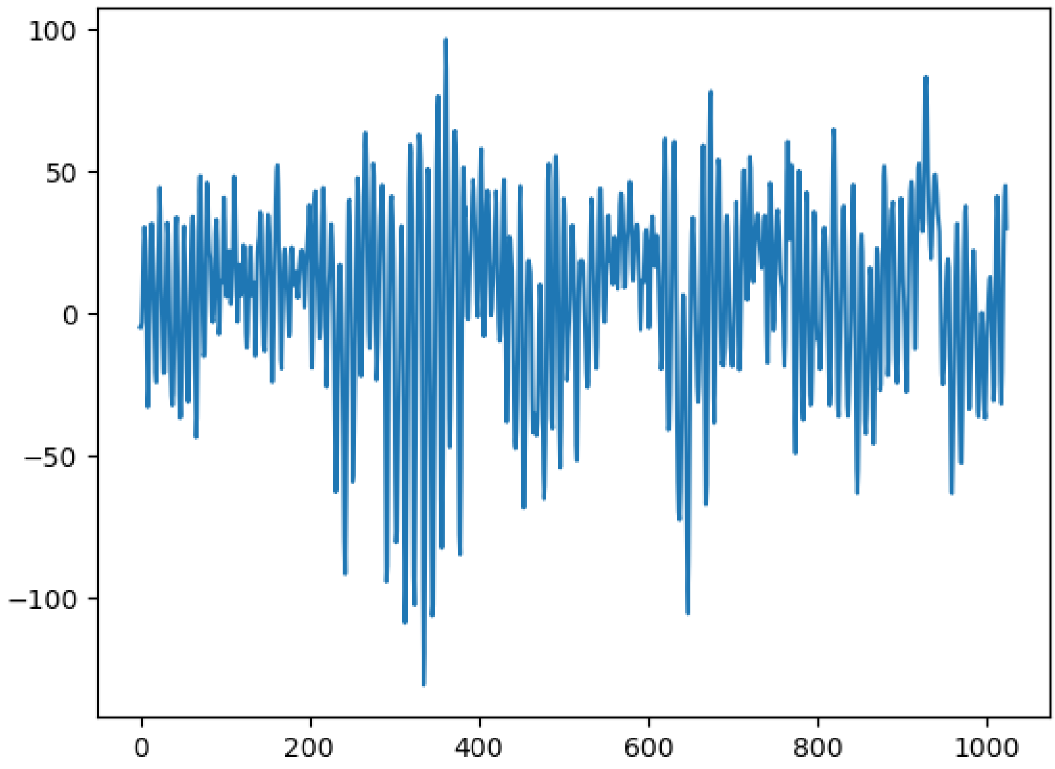
100
50
0
−50
−100
0
200
400
600
800
1000

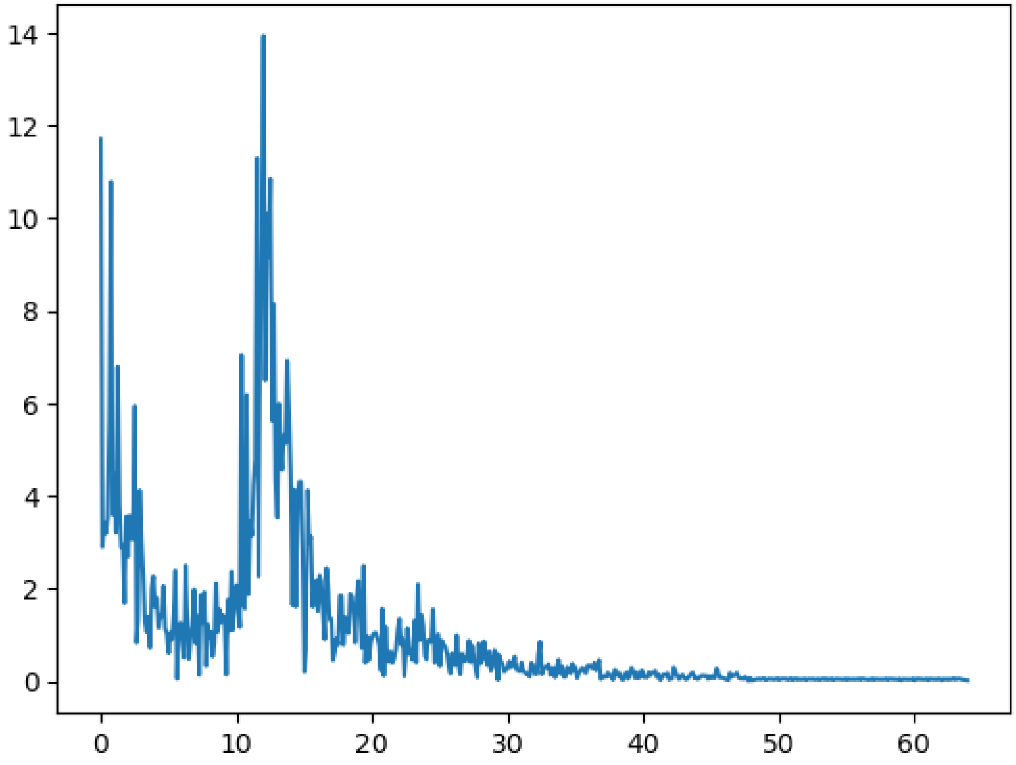
14
12
10
8
6
4
2
0
0
10
20
30
40
50
60

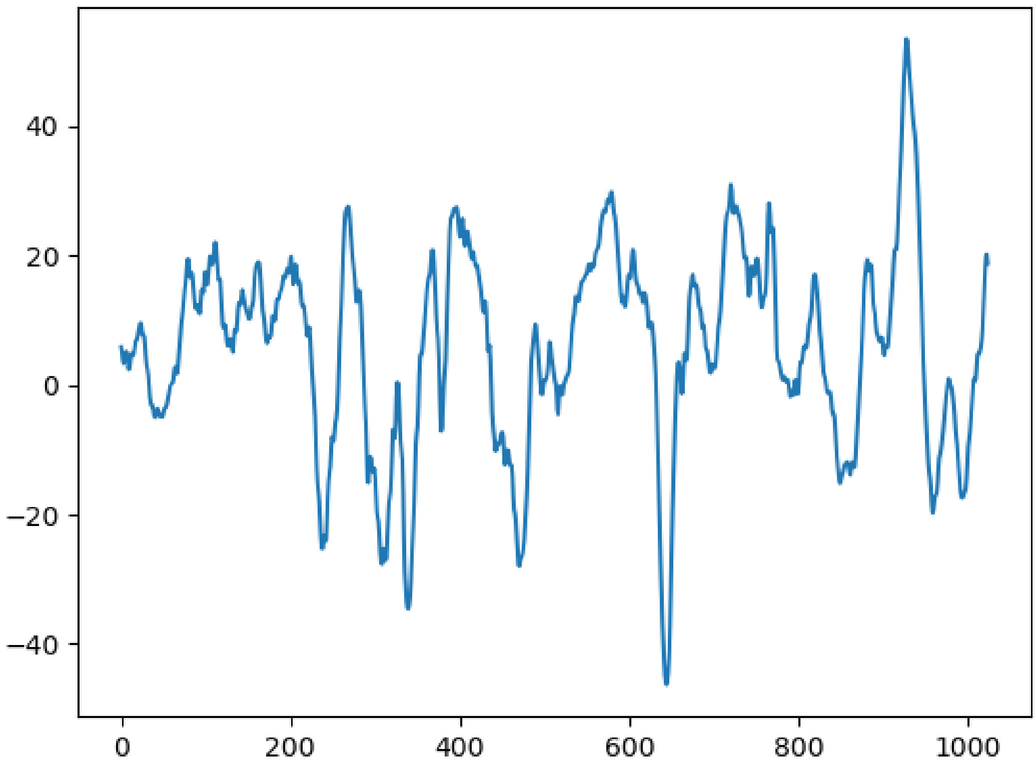
40
20
0
−20
−40
0
200
400
600
800
1000

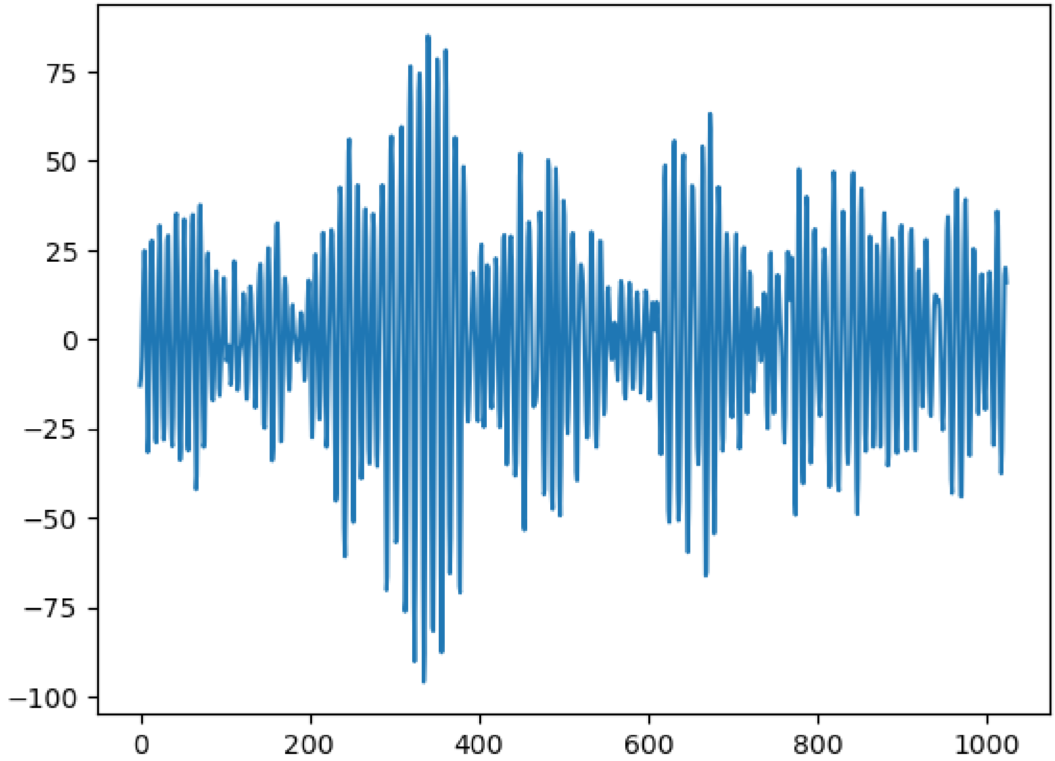

[35]:
```
dt = 1/128
sig = eeg_1024
env_0 = np.abs(hilbert(sig))
p_0 = env_0 / sum(env_0)
ent(p_0)
```

[35]: -9.788829994422958

[36]:
```
np.sqrt(sum(eeg_1024 ** 2)/len(eeg_1024))
```

[36]: 33.26682698039821

[37]:
```
dt = 1/128
sig = u_t[:,0]
env_1 = np.abs(hilbert(sig))
p_1 = env_1 / sum(env_1)
ent(p_1)
```

[37]: -9.825626020288496

[38]:
```
np.sqrt(sum(u_t[:,0] ** 2)/len(u_t[:,0]))
```

[38]: 16.324733478482642

[39]:
```
dt = 1/128
sig = u_t[:,1]
env_2 = np.abs(hilbert(sig))
p_2 = env_2 / sum(env_2)
ent(p_2)
```

[39]: -9.808713154776052

[40]:
```
np.sqrt(sum(u_t[:,1] ** 2)/len(u_t[:,1]))
```

[40]: 27.52689928988346

[41]:
```
#dt = 1/128
#sig = u_t[:,2]
#env_3 = np.abs(hilbert(sig))
#p_3 = env_3 / sum(env_3)
#ent(p_3)
```

[42]:
```
#np.sqrt(sum(u_t[:,2] ** 2)/len(u_t[:,2]))
```

[43]:
```
#dt = 1/128
#sig = u_t[:,3]
#env_4 = np.abs(hilbert(sig))
#p_4 = env_4 / sum(env_4)
#ent(p_4)
```

[44]:
```
#dt = 1/128
#sig = u_t[:,4]
#env_5 = np.abs(hilbert(sig))
#p_5 = env_5 / sum(env_5)
#ent(p_5)
```

[45]:
```
#dt = 1/128
#sig = u_t[:,5]
#env_6 = np.abs(hilbert(sig))
#p_6 = env_6 / sum(env_6)
#ent(p_6)
```

[46]:
```
(ent(p_1)+ent(p_2))/2
#(ent(p_1)+ent(p_2)+ent(p_3))/3
#(ent(p_1)+ent(p_2)+ent(p_3)+ent(p_4))/4
#(ent(p_1)+ent(p_2)+ent(p_3)+ent(p_4)+ent(p_5))/5
#(ent(p_1)+ent(p_2)+ent(p_3)+ent(p_4)+ent(p_5)+ent(p_6))/6
```

[46]: -9.817169587532273

```
[47]: #. Visualize decomposed modes
plt.figure()
plt.subplot(2,1,1)
plt.plot(eeg_1024)
plt.title('Original signal')
plt.xlabel('time (s)')
plt.subplot(2,1,2)
plt.plot(u.T)
plt.title('Decomposed modes')
plt.xlabel('time (s)')
plt.legend(['Mode %d'%(m_i+1) for m_i in range(u.shape[0])])
plt.tight_layout()
plt.savefig('data_eeg_prop_2024_1S/fig_14.svg')
```

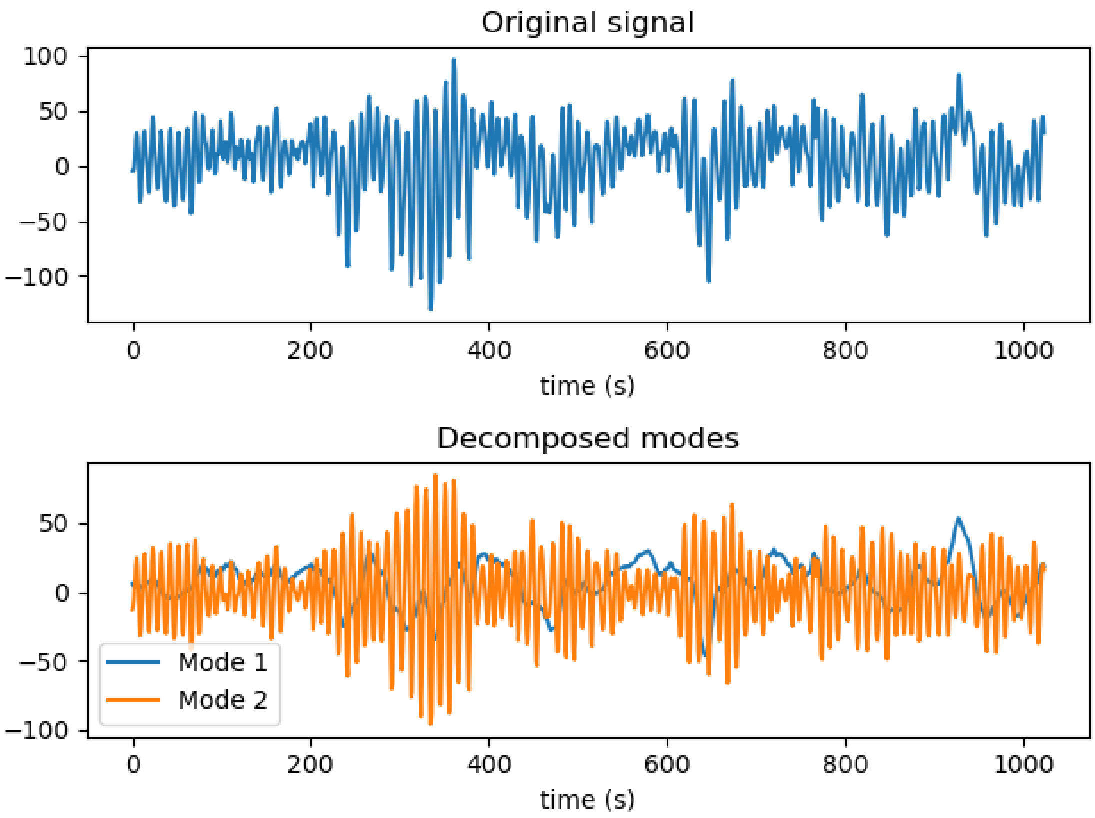


```
[48]: #. Visualize decomposed modes
plt.figure()
plt.subplot(2,1,1)
plt.plot(u.T)
plt.title('Decomposed modes')
plt.xlabel('time (s)')
```

```
plt.subplot(2,1,2)
plt.plot(env_1.T)
plt.plot(env_2.T)
#plt.plot(env_3.T)
plt.title('Envelops of decomposed modes')
plt.xlabel('time (s)')
plt.legend(['Mode %d'%(m_i+1) for m_i in range(u.shape[0])])
plt.tight_layout()
plt.savefig('data_eeg_prop_2024_1S/fig_15.svg')
```

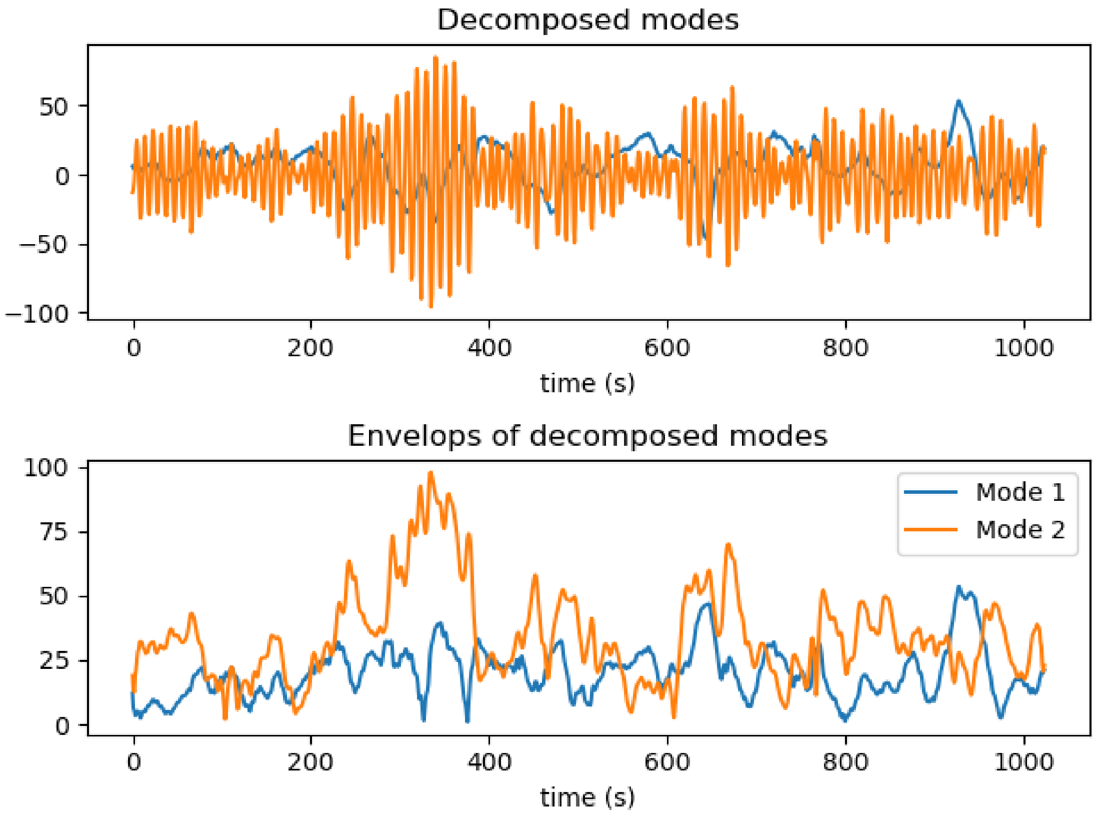

9 # EMD & VMD after GWO

[49]:
```
# Get the default configuration for a sift
config = emd.sift.get_config('sift')
# Adjust the threshold for accepting an IMF
config['imf_opts/sd_thresh'] = 0.05
imf = emd.sift.sift(signal)
```

[50]:
```
emd.plotting.plot_imfs(imf, cmap=True)
plt.savefig('data_eeg_prop_2024_1S/emd2.svg')
```

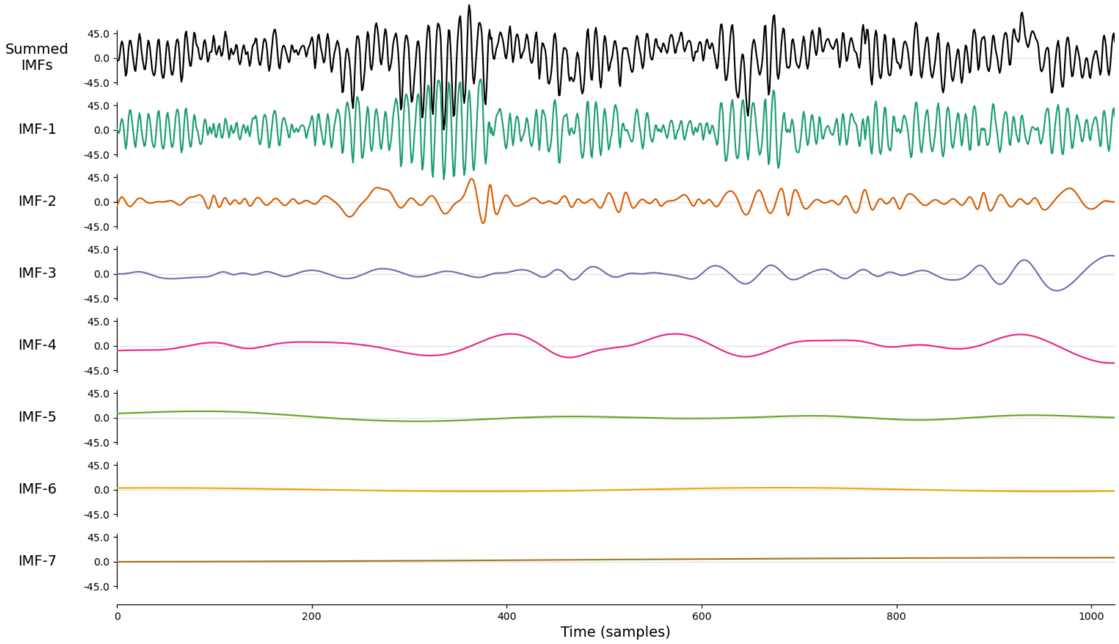

[51]:
```
emd.plotting.plot_imfs(u_t, cmap=True)
plt.savefig('data_eeg_prop_2024_1S/vmd2.svg')
```

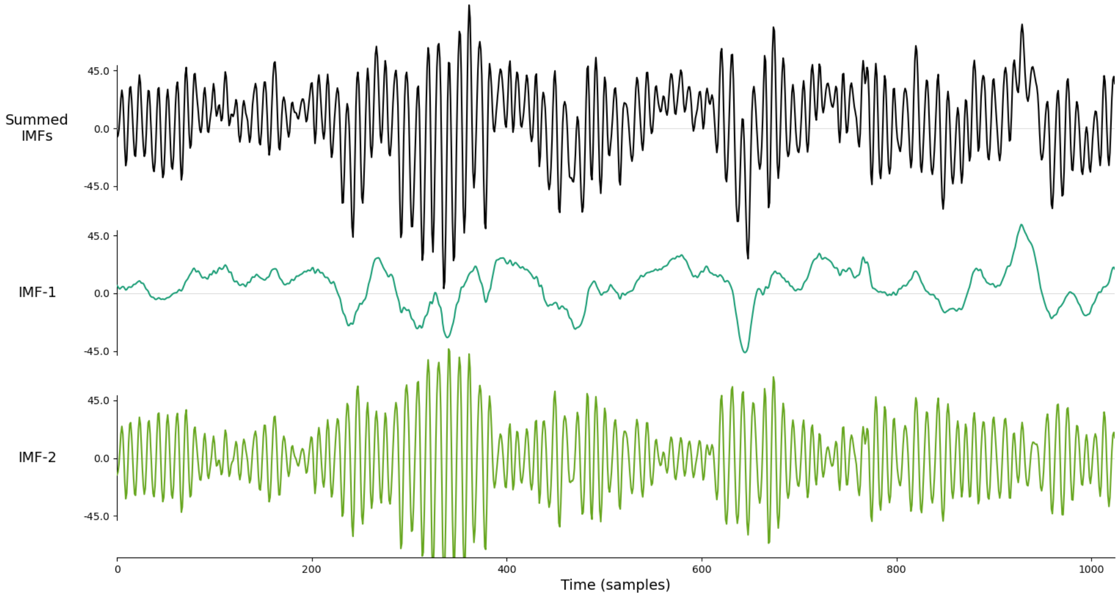

10 # Hilbert Spectrogram

[52]:
```
sample_rate = 128
seconds = 8
num_samples = sample_rate*seconds
time_vect = np.linspace(0, seconds, num_samples)
```

[53]:
```
IP, IF, IA = emd.spectra.frequency_transform(u_t, sample_rate, 'hilbert')
```

[54]:
```
freq_edges, freq_bins = emd.spectra.define_hist_bins(0.1, 64, 128)
#hht = emd.spectra.hilberthuang(IF, IA, freq_edges)

freq_range = (0, 64, 128)  # 0 to 64Hz in 128 steps
f, hht = emd.spectra.hilberthuang(IF, IA, freq_range, sum_time=False)
```

[55]:
```
freq_edges, freq_centres = emd.spectra.define_hist_bins(0, 100, 128, 'linear')

# Amplitude weighted HHT per IMF
f, spec_weighted = emd.spectra.hilberthuang(IF, IA, freq_edges, sum_imfs=False)

# Unweighted HHT per IMF - we replace the instantaneous amplitude values with␣
↪ones
f, spec_unweighted = emd.spectra.hilberthuang(IF, np.ones_like(IA), freq_edges,␣
↪sum_imfs=False)
```

[56]:
```
plt.figure(figsize=(10, 4))
plt.subplots_adjust(hspace=0.4)
plt.subplot(121)
plt.plot(freq_centres, spec_unweighted)
plt.xticks(np.arange(10)*10)
plt.xlim(0, 47)
plt.ylim(0, 800)
plt.xlabel('Frequency (Hz)')
plt.ylabel('Count')
plt.title('unweighted\nHilbert-Huang Transform')

plt.subplot(122)
plt.plot(freq_centres, spec_weighted)
plt.xticks(np.arange(10)*10)
plt.xlim(0, 47)
plt.xlabel('Frequency (Hz)')
plt.ylabel('Power')
plt.title('IA-weighted\nHilbert-Huang Transform')
plt.legend(['IMF-1', 'IMF-2', 'IMF-3', 'IMF-4', 'IMF-5', 'IMF-6'],␣
↪frameon=False)
plt.savefig('data_eeg_prop_2024_1S/HHT_freq.svg')
```

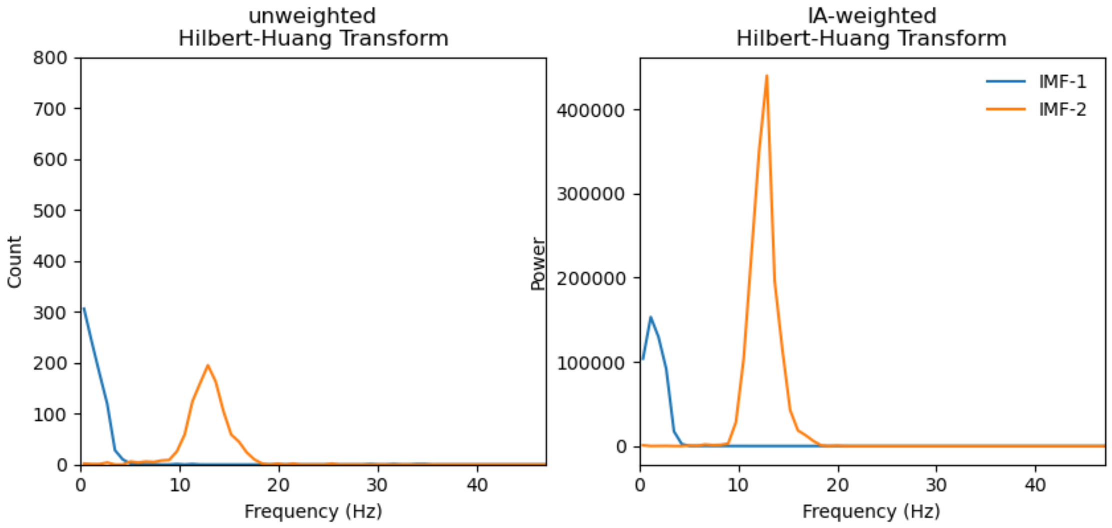

[57]:
```
fig = plt.figure(figsize=(6, 4))
emd.plotting.plot_hilberthuang(hht, time_vect, freq_bins,
                               time_lims=(0, 8), freq_lims=(0.1, 47), vmax=100,
                               fig=fig, log_y=False)
plt.savefig('data_eeg_prop_2024_1S/HHT_spectrum-1.svg')
```

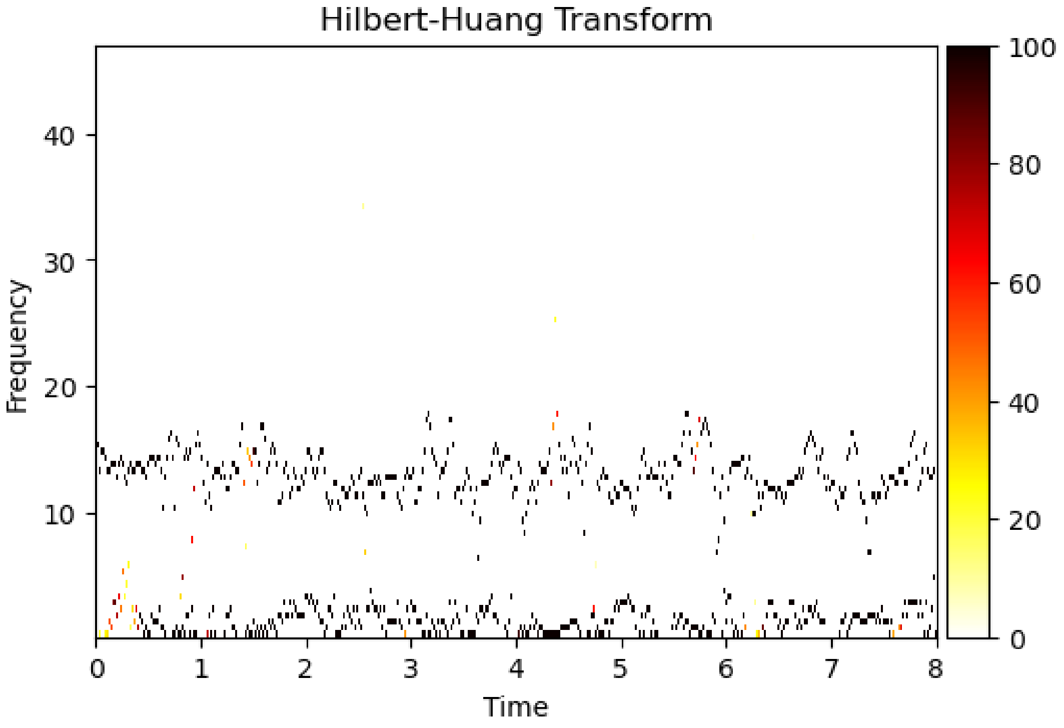

[58]:
```python
fig = plt.figure(figsize=(6, 4))
emd.plotting.plot_hilberthuang(hht, time_vect, freq_bins,cmap='ocean_r',
                               time_lims=(0, 8), freq_lims=(0.1, 47), vmax=100,
                               fig=fig, log_y=False)
plt.savefig('data_eeg_prop_2024_1S/HHT_spectrum-1_1.svg')
```

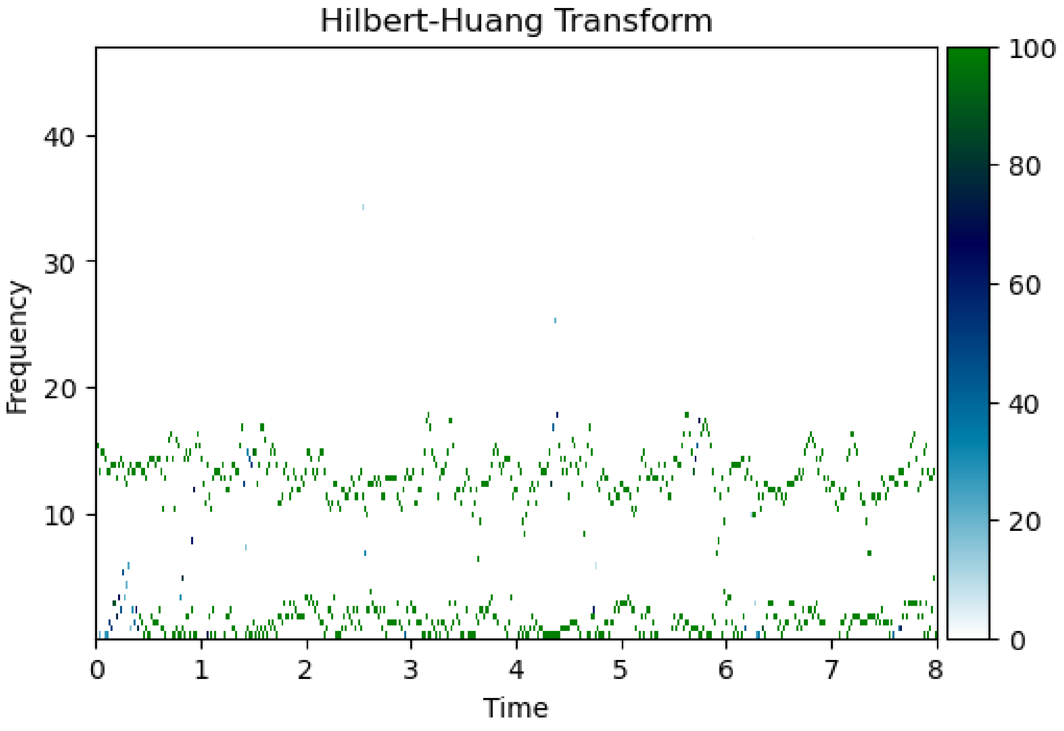

[59]:
```python
print(emd.plotting.plot_hilberthuang.__doc__)
```

```
Create a quick summary plot for a Hilbert-Huang Transform.

    Parameters
    ----------
    hht : 2d array
        Hilbert-Huang spectrum to be plotted - output from
emd.spectra.hilberthuang
    time_vect : vector
        Vector of time samples
    freq_vect : vector
        Vector of frequency bins
    time_lims : optional tuple or list (start_val, end_val)
        Optional time-limits to zoom in time on the x-axis
    freq_lims : optional tuple or list (start_val, end_val)
        Optional time-limits to zoom in frequency on the y-axis
    fig : optional figure handle
```

```
        Figure to plot inside
    ax : optional axis handle
        Axis to plot inside
    cmap : optional str or matplotlib.cm
        Colormap specification

    Returns
    -------
    ax
        Handle of plot axis
```

[60]:
```python
fig = plt.figure(figsize=(6, 4))
emd.plotting.plot_hilberthuang(hht, time_vect, freq_bins, cmap='jet',
                               time_lims=(0, 8), freq_lims=(0.1, 47),vmax=100,
                               fig=fig, log_y=False)
plt.savefig('data_eeg_prop_2024_1S/HHT_spectrum-2.svg')
```

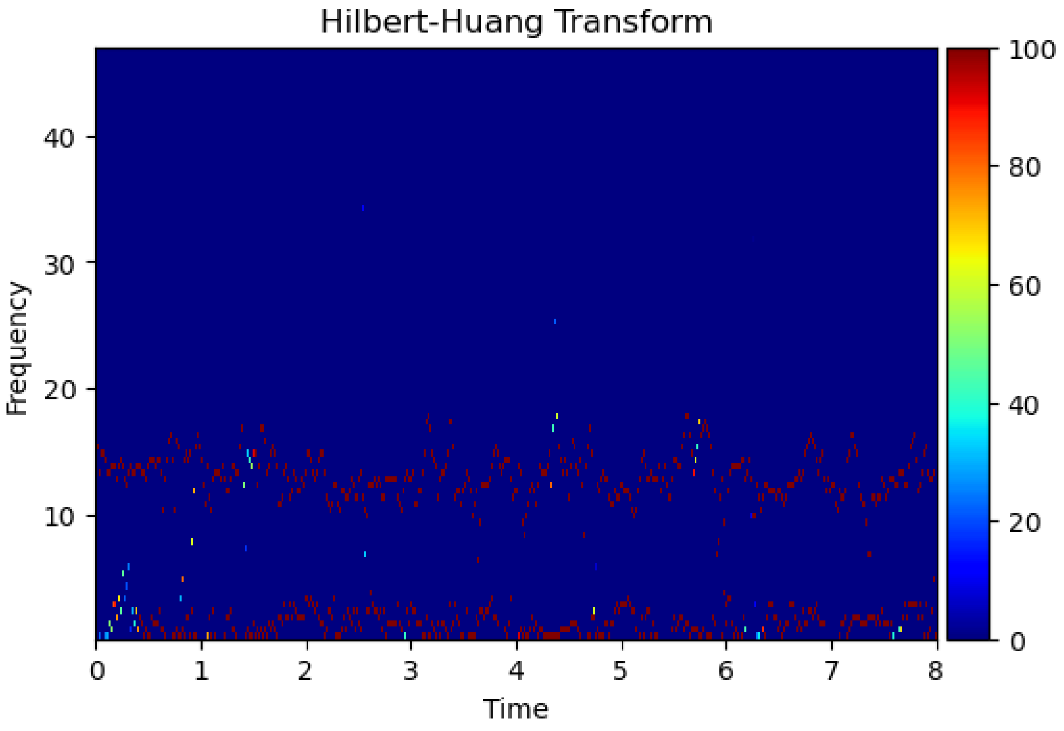

[61]:
```python
fig = plt.figure(figsize=(6, 4))
emd.plotting.plot_hilberthuang(hht, time_vect, freq_bins, cmap='gist_heat_r',
                               time_lims=(0, 8), freq_lims=(0.1, 47), vmax=100,
                               fig=fig, log_y=False)
```

```
plt.savefig('data_eeg_prop_2024_1S/HHT_spectrum-3.svg')
```

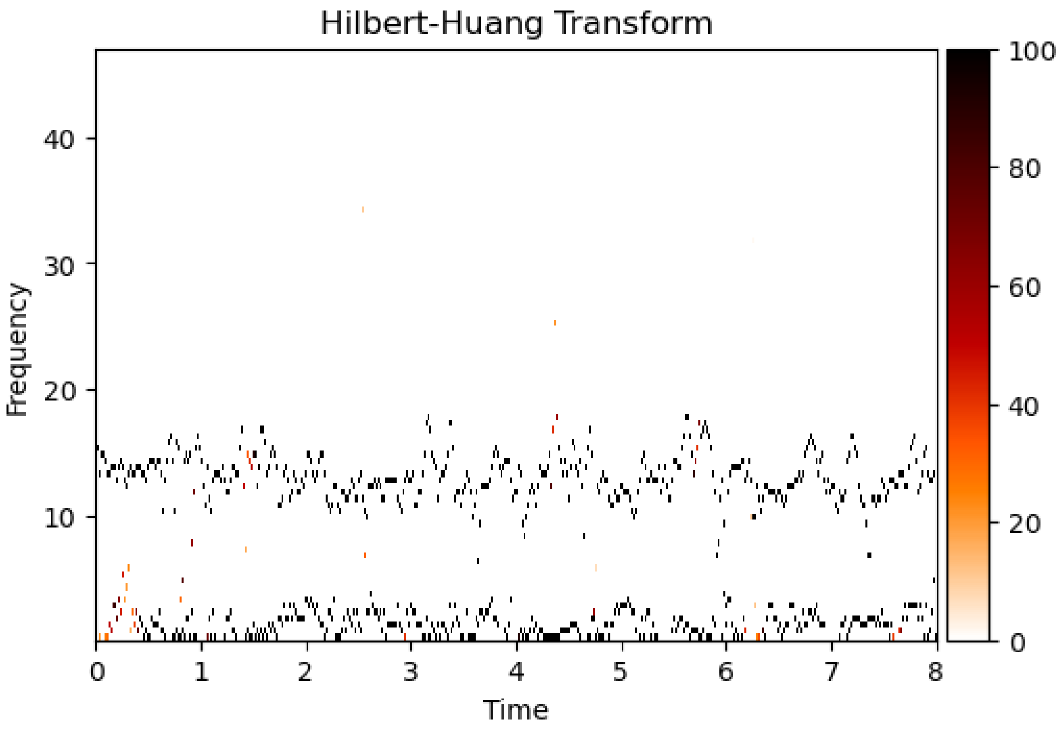

11 #whole data graph for 10 min

[62]:
```
T1=1024
```

[63]:
```
time = df_eeg['Time'].values
eeg = df_eeg['eeg'].values
```

[64]:
```
int(len(df_eeg)/T1)
```

[64]: 73

[65]:
```
eeg_list = []
```

[66]:
```
for i in range(int(len(eeg)/T1)):
    eeg_list.append(eeg[i*T1:i*T1+T1])
```

[67]:
```
eeg_list[72]
```

[67]: array([-119.65, -190.05, -155.6 , …, -61.45, -45.35, -92.6])

[68]:
```
# VMD algorythm without graphs
def  VMD2(f, alpha, tau, K, DC, init, tol):
    """
    u,u_hat,omega = VMD(f, alpha, tau, K, DC, init, tol)
    Variational mode decomposition
    Python implementation by Vinícius Rezende Carvalho - vrcarva@gmail.com
    code based on Dominique Zosso's MATLAB code, available at:
    https://www.mathworks.com/matlabcentral/fileexchange/
↪44765-variational-mode-decomposition
    Original paper:
    Dragomiretskiy, K. and Zosso, D. (2014) 'Variational Mode Decomposition',
    IEEE Transactions on Signal Processing, 62(3), pp. 531-544. doi: 10.1109/
↪TSP.2013.2288675.


    Input and Parameters:
    ---------------------
    f       - the time domain signal (1D) to be decomposed
    alpha   - the balancing parameter of the data-fidelity constraint
    tau     - time-step of the dual ascent ( pick 0 for noise-slack )
    K       - the number of modes to be recovered
    DC      - true if the first mode is put and kept at DC (0-freq)
    init    - 0 = all omegas start at 0
                       1 = all omegas start uniformly distributed
                      2 = all omegas initialized randomly
    tol     - tolerance of convergence criterion; typically around 1e-6

    Output:
    -------
    u       - the collection of decomposed modes
    u_hat   - spectra of the modes
    omega   - estimated mode center-frequencies

    # ---------------------
    #  signal  - the time domain signal (1D) to be decomposed
    #
    #  alpha   - the balancing parameter of the data-fidelity constraint
    #
    #  tau     - time-step of the dual ascent ( pick 0 for noise-slack )
    #
    #  K       - the number of modes to be recovered
    #  DC      - true if the first mode is put and kept at DC (0-freq)
    #            DC
    #  init    - 0 = all omegas start at 0
    #                    1 = all omegas start uniformly distributed
    #                    2 = all omegas initialized randomly
    #  tol     - tolerance of convergence criterion; typically around 1e-6
```

```python
    #                    1e-6
    #
    #  Output:
    #  -------
    #  u       - the collection of decomposed modes
    #  u_hat   - spectra of the modes
    #  omega   - estimated mode center-frequencies
    #
    """

    if len(f)%2:
       f = f[:-1]

    # Period and sampling frequency of input signal
    fs = 1./len(f)

    ltemp = len(f)//2
    fMirr =  np.append(np.flip(f[:ltemp],axis = 0),f)
    fMirr = np.append(fMirr,np.flip(f[-ltemp:],axis = 0))

    # Time Domain 0 to T (of mirrored signal)
    T = len(fMirr)
    t = np.arange(1,T+1)/T

    # Spectral Domain discretization
    freqs = t-0.5-(1/T)

    # Maximum number of iterations (if not converged yet, then it won't anyway)
    Niter = 500
    # For future generalizations: individual alpha for each mode
    Alpha = alpha*np.ones(K)

    # Construct and center f_hat
    f_hat = np.fft.fftshift((np.fft.fft(fMirr)))
    f_hat_plus = np.copy(f_hat) #copy f_hat
    f_hat_plus[:T//2] = 0

    # Initialization of omega_k
    omega_plus = np.zeros([Niter, K])


    if init == 1:
        for i in range(K):
            omega_plus[0,i] = (0.5/K)*(i)
    elif init == 2:
        omega_plus[0,:] = np.sort(np.exp(np.log(fs) + (np.log(0.5)-np.
↪log(fs))*np.random.rand(1,K)))
```

```
    else:
        omega_plus[0,:] = 0

    # if DC mode imposed, set its omega to 0
    if DC:
        omega_plus[0,0] = 0

    # start with empty dual variables
    lambda_hat = np.zeros([Niter, len(freqs)], dtype = complex)

    # other inits
    uDiff = tol+np.spacing(1) # update step
    n = 0 # loop counter
    sum_uk = 0 # accumulator
    # matrix keeping track of every iterant // could be discarded for mem
    u_hat_plus = np.zeros([Niter, len(freqs), K],dtype=complex)

    #*** Main loop for iterative updates***

    while ( uDiff > tol and  n < Niter-1 ): # not converged and below␣
↪iterations limit
        # update first mode accumulator
        k = 0
        sum_uk = u_hat_plus[n,:,K-1] + sum_uk - u_hat_plus[n,:,0]

        # update spectrum of first mode through Wiener filter of residuals
        u_hat_plus[n+1,:,k] = (f_hat_plus - sum_uk - lambda_hat[n,:]/2)/(1.
↪+Alpha[k]*(freqs - omega_plus[n,k])**2)

        # update first omega if not held at 0
        if not(DC):
            omega_plus[n+1,k] = np.dot(freqs[T//2:T],(abs(u_hat_plus[n+1, T//2:
↪T, k])**2))/np.sum(abs(u_hat_plus[n+1,T//2:T,k])**2)

        # update of any other mode
        for k in np.arange(1,K):
            #accumulator
            sum_uk = u_hat_plus[n+1,:,k-1] + sum_uk - u_hat_plus[n,:,k]
            # mode spectrum
            u_hat_plus[n+1,:,k] = (f_hat_plus - sum_uk - lambda_hat[n,:]/2)/
↪(1+Alpha[k]*(freqs - omega_plus[n,k])**2)
            # center frequencies
            omega_plus[n+1,k] = np.dot(freqs[T//2:T],(abs(u_hat_plus[n+1, T//2:
↪T, k])**2))/np.sum(abs(u_hat_plus[n+1,T//2:T,k])**2)

        # Dual ascent
```

```
        lambda_hat[n+1,:] = lambda_hat[n,:] + tau*(np.sum(u_hat_plus[n+1,:,:
↪],axis = 1) - f_hat_plus)

        # loop counter
        n = n+1

        # converged yet?
        uDiff = np.spacing(1)
        for i in range(K):
            uDiff = uDiff + (1/T)*np.dot((u_hat_plus[n,:,i]-u_hat_plus[n-1,:
↪,i]),np.conj((u_hat_plus[n,:,i]-u_hat_plus[n-1,:,i])))

        uDiff = np.abs(uDiff)

    #Postprocessing and cleanup

    #discard empty space if converged early
    Niter = np.min([Niter,n])
    omega = omega_plus[:Niter,:]

    idxs = np.flip(np.arange(1,T//2+1),axis = 0)
    # Signal reconstruction
    u_hat = np.zeros([T, K],dtype = complex)
    u_hat[T//2:T,:] = u_hat_plus[Niter-1,T//2:T,:]
    u_hat[idxs,:] = np.conj(u_hat_plus[Niter-1,T//2:T,:])
    u_hat[0,:] = np.conj(u_hat[-1,:])

    u = np.zeros([K,len(t)])
    for k in range(K):
        u[k,:] = np.real(np.fft.ifft(np.fft.ifftshift(u_hat[:,k])))

    # remove mirror part
    u = u[:,T//4:3*T//4]

    # recompute spectrum
    u_hat = np.zeros([u.shape[1],K],dtype = complex)
    for k in range(K):
        u_hat[:,k]=np.fft.fftshift(np.fft.fft(u[k,:]))


    for k in range(1,K+1):
        filename_ifft = "data_eeg_prop_2024_1S/{}_imf-%d.txt" .format(str(n).
↪zfill(3)) %(k)
        np.savetxt(filename_ifft, u[k-1,:])

    #recompute spectrum      IMF
    u_hat = np.zeros((T//2, K),dtype=complex)
```

```
    for k in range(1,K+1):
        u_hat[:, k-1]= ((np.fft.fft(u[k-1,:])).conj()).T

    u_t =u.T

    return (u,u_t,u_hat,omega)
```

12 # VMD K=3, PF=2000

[69]:
```
alpha = 2000
tau = 0
DC = 0
init = 0
tol = 1e-7
K = 2

ut_list = []

for i in range(int(len(eeg)/T1)):
#for i in range(2):
    u,u_t,u_hat,omega = VMD2(eeg_list[i], alpha, tau, K, DC, init, tol)
    ut_list.append(u_t)
```

[70]:
```
sample_rate = 128
seconds = 584
num_samples = sample_rate*seconds
time_vect = np.linspace(0, seconds, num_samples)

IP_list = []
IF_list = []
IA_list = []
f_list = []
hht_list = []

for i in range(int(len(eeg)/T1)):
#for i in range(2):
    IP, IF, IA = emd.spectra.frequency_transform(ut_list[i], sample_rate,␣
 ↪'hilbert')
    IP_list.append(IP)
    IF_list.append(IF)
    IA_list.append(IA)
    freq_range = (0, 64, 128)  # 0 to 64Hz in 128 steps
    f, hht = emd.spectra.hilberthuang(IF_list[i], IA_list[i], freq_range,␣
 ↪sum_time=False)
    f_list.append(f)
```

```
    hht_list.append(hht)

freq_edges, freq_centres = emd.spectra.define_hist_bins(0, 100, 128, 'linear')

# Unweighted HHT per IMF - we replace the instantaneous amplitude values with␣
↪ones
f, spec_unweighted = emd.spectra.hilberthuang(IF_list[0], np.ones_like(IA),␣
↪freq_edges, sum_imfs=False)
```

[71]:
```
plt.figure(figsize=(4, 4))
#plt.subplots_adjust(hspace=0.4)
#plt.subplot(121)
plt.plot(freq_centres, spec_unweighted)
plt.xticks(np.arange(10)*10)
plt.xlim(0, 47)
plt.ylim(0, 800)
plt.xlabel('Frequency (Hz)')
plt.ylabel('Count')
plt.title('unweighted\nHilbert-Huang Transform')
plt.legend(['IMF-1', 'IMF-2', 'IMF-3', 'IMF-4', 'IMF-5', 'IMF-6'],␣
↪frameon=False)
plt.savefig('data_eeg_prop_2024/HHT_freq_1.svg')
```

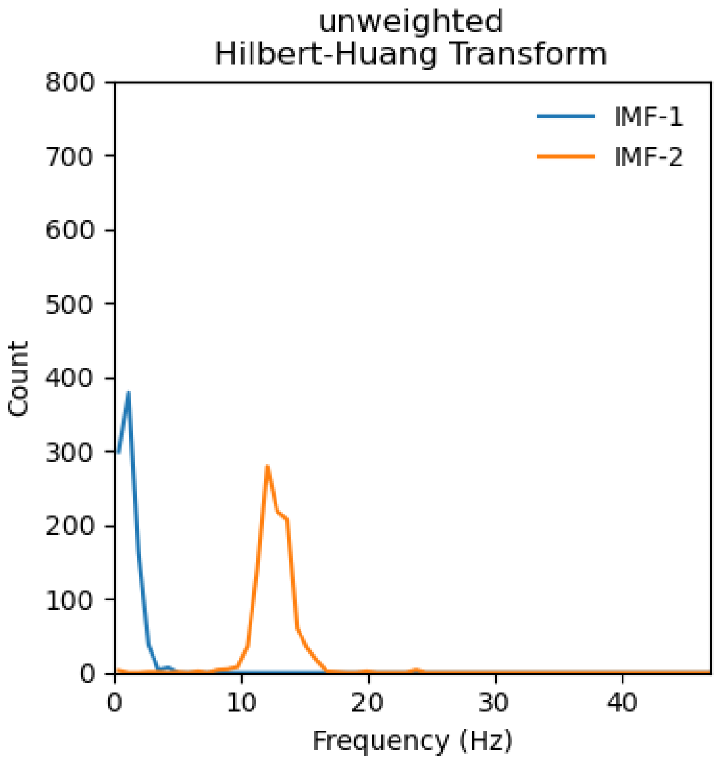

[72]:
```
hht_con = hht_list[0]
for i in range(int(len(eeg)/T1)-1):
    hht_con = np.concatenate([hht_con, hht_list[i+1]], 1)
```

[73]:
```
fig = plt.figure(figsize=(12, 4))
emd.plotting.plot_hilberthuang(hht_con, time_vect, freq_bins, cmap='ocean_r',
                               time_lims=(0, 584), freq_lims=(0.1, 47), vmax=1,
                               fig=fig, log_y=False)
plt.savefig('data_eeg_prop_2024/HHT_spectrum-1_1.svg')
plt.savefig('data_eeg_prop_2024/HHT_spectrum-1_1.png')
```

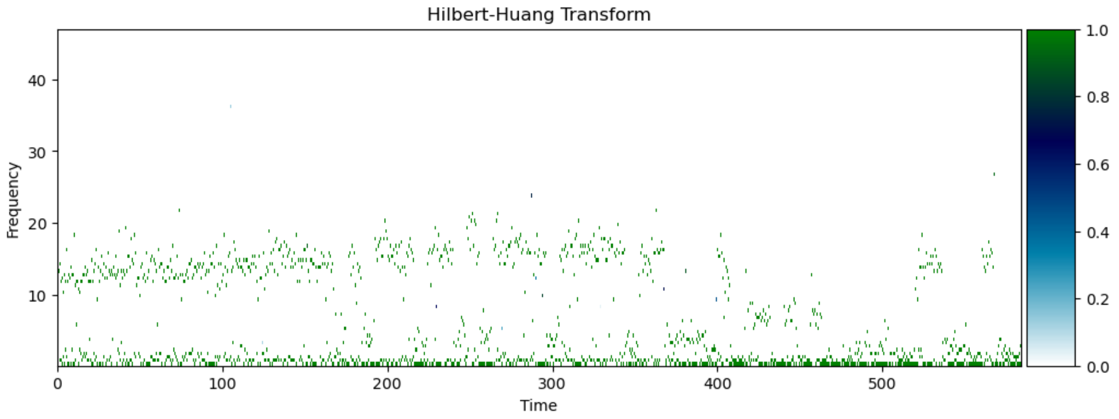

13 # VMD K=3, PF=2000

[74]:
```
alpha = 2000
tau = 0
DC = 0
init = 0
tol = 1e-7
K = 3

ut_list = []

for i in range(int(len(eeg)/T1)):
#for i in range(2):
    u,u_t,u_hat,omega = VMD2(eeg_list[i], alpha, tau, K, DC, init, tol)
    ut_list.append(u_t)
```

[75]:
```
sample_rate = 128
seconds = 584
num_samples = sample_rate*seconds
time_vect = np.linspace(0, seconds, num_samples)
```

```
IP_list = []
IF_list = []
IA_list = []
f_list = []
hht_list = []

for i in range(int(len(eeg)/T1)):
#for i in range(2):
    IP, IF, IA = emd.spectra.frequency_transform(ut_list[i], sample_rate,␣
 ↪'hilbert')
    IP_list.append(IP)
    IF_list.append(IF)
    IA_list.append(IA)
    freq_range = (0, 64, 128)  # 0 to 64Hz in 128 steps
    f, hht = emd.spectra.hilberthuang(IF_list[i], IA_list[i], freq_range,␣
 ↪sum_time=False)
    f_list.append(f)
    hht_list.append(hht)

freq_edges, freq_centres = emd.spectra.define_hist_bins(0, 100, 128, 'linear')

# Unweighted HHT per IMF - we replace the instantaneous amplitude values with␣
 ↪ones
f, spec_unweighted = emd.spectra.hilberthuang(IF_list[0], np.ones_like(IA),␣
 ↪freq_edges, sum_imfs=False)
```

[76]:
```
plt.figure(figsize=(4, 4))
#plt.subplots_adjust(hspace=0.4)
#plt.subplot(121)
plt.plot(freq_centres, spec_unweighted)
plt.xticks(np.arange(10)*10)
plt.xlim(0, 47)
plt.ylim(0, 800)
plt.xlabel('Frequency (Hz)')
plt.ylabel('Count')
plt.title('unweighted\nHilbert-Huang Transform')
plt.legend(['IMF-1', 'IMF-2', 'IMF-3', 'IMF-4', 'IMF-5', 'IMF-6'],␣
 ↪frameon=False)
plt.savefig('data_eeg_prop_2024/HHT_freq_2.svg')
```

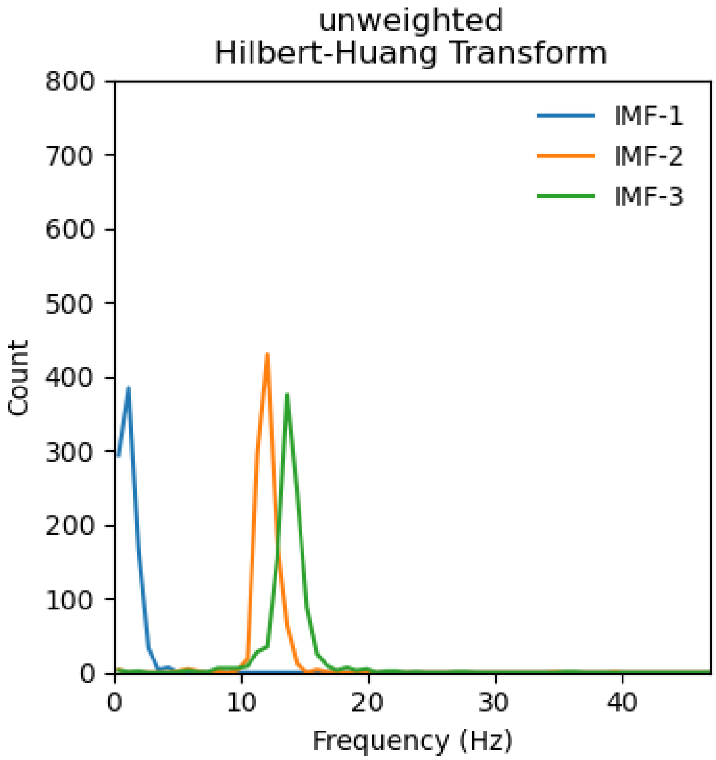

[77]:
```
hht_con = hht_list[0]
for i in range(int(len(eeg)/T1)-1):
    hht_con = np.concatenate([hht_con, hht_list[i+1]], 1)
```

[78]:
```
fig = plt.figure(figsize=(12, 4))
emd.plotting.plot_hilberthuang(hht_con, time_vect, freq_bins, cmap='ocean_r',
                               time_lims=(0, 584), freq_lims=(0.1, 47), vmax=1,
                               fig=fig, log_y=False)
plt.savefig('data_eeg_prop_2024/HHT_spectrum-1_2.svg')
plt.savefig('data_eeg_prop_2024/HHT_spectrum-1_2.png')
```

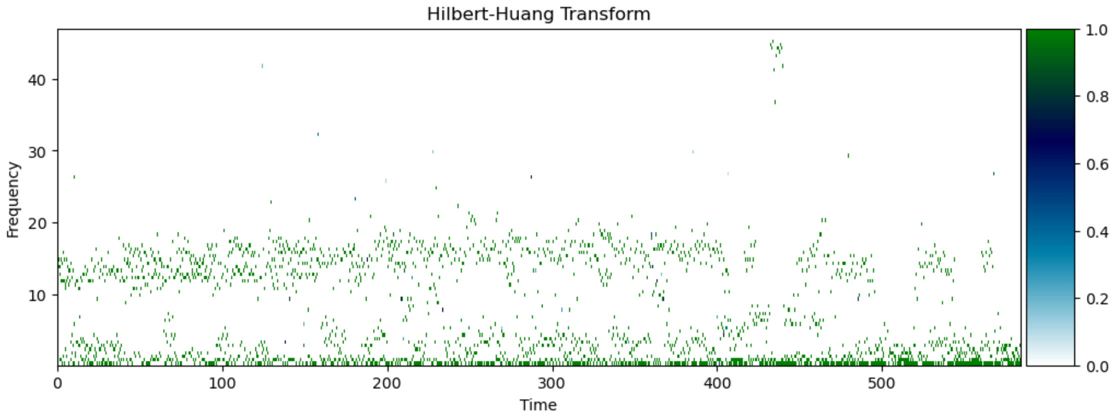

Supplement: Supplementary file 1 [file sensors-24-05749-s001.zip › Supple2_ProgramCode_Python_VMD-GWO.pdf]
