# Supplementary material for: Variational Mode Decomposition Analysis of Electroencephalograms during General Anesthesia: Using the Grey Wolf Optimizer to Determine Hyperparameters
Source: Sensors (Basel). 2024 Sep 4;24(17):5749. doi: 10.3390/s24175749 (PMC11398215; doi:10.3390/s24175749)
Supplement: Supplementary file 1 [file sensors-24-05749-s001.zip › Supple3_ProgramCode2_Processing_code.pdf]

```
// For Float Class, download FloatTable.pde
// from Ben Fry's Visualizing Data (https://benfry.com),
// and put "FloatTable.pde" in the same folder. with the following
GWO.pde & VMD.pde

//*****GWO.pde******
//
*******************************************************************
// Grey Wolf Optimization for Variational Mode Decomposition
//** Teiji Sawa
//** Created: Mar 10, 2024
//
*******************************************************************

//
*******************************************************************
// DATA file selection
String input_datafile = "PRO1_eeg_bis_20210319_EME10.tsv"; //pro-1
//String input_datafile = "PRO2_eeg_bis_20220614_EME10.tsv"; //pro-2
//String input_datafile = "PRO3_eeg_bis_20220628_EME10.tsv"; //pro-3
//String input_datafile = "PRO4_eeg_bis_20220629_EME10.tsv"; //pro-4
//
*******************************************************************

//
*******************************************************************
// Console oputput file selection:
String output_datafile = "Prop4_max_iter20_test.txt";
//
*******************************************************************

//
*******************************************************************
int current_row = 0;
//
*******************************************************************
// GWO SETTING
int  num_particles = 20;
int  max_iter =  20;
int  seed = 1;
float  minx1 = 2;
float  maxx1 = 6;
float  minx2 = 1;
float  maxx2 = 500;
//
*******************************************************************

import com.github.psambit9791.jdsp.transform.Hilbert;
import java.util.Map.Entry;

import java.util.Comparator;
import java.util.Arrays;
import java.util.List;
```

```
import java.util.ArrayList;
import java.util.Collections;
import java.util.Random;
import java.util.*;
import java.io.FileOutputStream;
import java.io.PrintStream;
import java.io.ByteArrayOutputStream;

import org.apache.commons.math3.transform.FastFourierTransformer;
import org.apache.commons.math3.complex.Complex;
import org.apache.commons.math3.transform.TransformType;
import org.apache.commons.math3.stat.Frequency;
import org.apache.commons.math3.transform.DftNormalization;

int T1 = 1024;
//INPUT DATA FILE NAME------------------
public static final Complex ZERO = new Complex(0, 0);
public static final Complex ONE = new Complex(1, 0);
public static final Complex TWO = new Complex(2, 0);
public static final Complex HALF = new Complex(0.5, 0);

Wolf3[] wolf;
Wolf3 alpha_wolf;
Wolf3 beta_wolf;
Wolf3 gamma_wolf;

Hilbert hilb[];
Hilbert hilb_ori;

double[] eeg_x1;
float[] eeg_x1_Hz;
int rowCount;
int columnCount;
double dt = 1/128;

int[] position_2D;
int[] best_position;
int wolf_no;

float fitness;
float fitness2;
float fitness_value;
float fitness_value2;
ArrayList population;
ArrayList population2;

double[][] x_env_double;
double[] x_env_ori_double;

float[] entropy;
double[] entropy_double;
double[][] power_data;
double[] power_data_ori;
double[] p;
```

```
double[] p_ori;
float[] p_float;
float[] p_ori_float;
double entropy_result;

double[][] x_env2_double;
float[] entropy2;
double[][] power_data2;
double[] p2;
float[] p2_float;
double entropy_result2;
double entropy_ori;

Complex[][] fft_result;
Complex[][] f_hat;
Complex[][] fft_result2;
Complex[][] f_hat2;
Complex[] f_eeg;

int X1;
int X2;
int X3;
int Xnew;
int Y1;
int Y2;
int Y3;
int Ynew;

//Initial Variables---------------------
int N = 2000;
//Complex alpha = new Complex(2000.0, 0);
Complex alpha;
Complex position_alpha;

Complex tau = ZERO;
int K = 10;
int optimized_K;
int optimized_alpha;
int DC = 0;
int init = 0;
double tol = 1e-7;

//INPUT DATA FILE NAME------------------
FloatTable eeg_data;

float t[];
double [][] u_t;
double [][] u_t2;
double [][] u_t3;
double [][] ewt;
double[][] imfs;
double x0[];
double[][] imfs_temp;
float[][] imfs_float;
```

```
double[] signal;

Vmd vmd_1;
Vmd vmd_2;
Vmd vmd_3;
Entropy entrop;

FileOutputStream outStr;
PrintStream printStream;
ByteArrayOutputStream baos;

void setup() {

  int  print_flag = 1;

  if (print_flag == 1) {
    println("This goes to the console.");
    try {

      //String file = dataPath("")+output_datafile;
      String file =dataPath("../user/"+output_datafile);

      if (!new File(dataPath("")).isDirectory()) {
        if (!new File(dataPath("")).mkdirs()) {
          System.err.println("Directory creation failed!");
          exit();
        }
      }
      FileOutputStream outStr = new FileOutputStream(file, false);
      PrintStream printStream = new PrintStream(outStr);
      System.setOut(printStream);
      System.setErr(printStream);
    }
    catch (IOException e) {
      System.err.println("Error! Check path, or filename, or
security manager! "+e);
      exit();
    }
    println("This goes to the file");
  }

  eeg_x1 = new double [T1];
  eeg_x1_Hz = new float [T1];

  signal = new double [T1];
  t = new float [2*T1];
  x0 = new double [T1];
  imfs_temp = new double [K][2*T1];
  imfs_float = new float[K][T1];
  imfs = new double [K][2*T1];
  u_t = new double [K][2*T1];
  u_t2 = new double [K][2*T1];
  hilb = new Hilbert[K];
```

```
  x_env_ori_double = new double [T1];
  power_data_ori = new double [T1];
  p_ori = new double [T1];
  p_ori_float = new float [T1];

  int T = 1024;
  float pi = PI;

  eeg_data = new FloatTable("data/" + input_datafile);
  rowCount = eeg_data.getRowCount();
  columnCount = eeg_data.getColumnCount();

  get_eegData();
  signal = eeg_x1;

  double entropy_result = entrop.calculateEntropy(signal);
  println("entropy_result1=:", entropy_result);


  hilb_ori = new Hilbert(signal);
  hilb_ori.hilbertTransform();
  //analytical_signal[j] = hilb[j].getOutput();
  x_env_ori_double = hilb_ori.getAmplitudeEnvelope();

  for (int i=0; i < T1; i++) {
    p_ori[i] = x_env_ori_double[i]/
array_sum_double(x_env_ori_double);
  }
  for (int i=0; i < T1; i++) {
    p_ori_float[i] = (float)(p_ori[i]);
  }

  entropy_ori= ent(p_ori_float);
  println("entropy_ori=", entropy_ori);

  for (int i = 0; i < T1; i++) {
    t[i] = (1/float(T1) * (i + 1));
  }

  println("Preparing Wolfs START:&&&&&&&&&&&&&&&&&&&&&&&&&&&&");
  // create n random wolves

  wolf_no = 0;
  ArrayList<Wolf3> population = new ArrayList<Wolf3>();

  wolf = new Wolf3[num_particles];

  for (int i=0; i < num_particles; i++) {
    wolf[i] = new Wolf3(signal, minx1, maxx1, minx2, maxx2, seed);
  }

  for (int i=0; i < num_particles; i++) {
    wolf[i].init(signal, minx1, maxx1, minx2, maxx2, i);
  }
```

```
  for (int i=0; i < num_particles; i++) {
    population.add(wolf[i]);
  }

  for (int i=0; i < num_particles; i++) {
    println("K-position-pro[", i, "] =",
((Wolf3)population.get(i)).getPosition1());
  }

  for (int i=0; i < num_particles; i++) {
    println("PF-position-pro[", i, "] =",
((Wolf3)population.get(i)).getPosition2());
  }

  for (int i=0; i < num_particles; i++) {
    println("KPF-fitness-pro[", i, "] =",
((Wolf3)population.get(i)).getFitness());
  }

  println("Wolfs K READY!:&&&&&&&&&&&&&&&&&&&&&&&&&&&&");
  println("GWO START:&&&&&&&&&&&&&&&&&&&&&&&&&&&&");

  best_position = gwo_KPF(population, signal, max_iter,
num_particles, minx1, maxx1, minx2, maxx2);

  println("%%%%%%%%%%%%%%%%%%%%%%%%%%");
  println("GWO completed.");
  println("Best solution found:");
  println("best K=:", best_position[0]);
  println("best PF=:", best_position[1]);
  println("%%%%%%%%%%%%%%%%%%%%%%%%%%");
  //  float err = vmd_gwo_3(best_position[0], best_position[1],
signal);
  double err = vmd_gwo_3(best_position[0], best_position[1],
signal);

  println("fitness of best solution = ", err);
  println("End GWO");
  println("=================================");
}

//-------fitness functions---------

float fitness_gwo(float _position) {

  fitness_value = 0.0;

  float xi = _position;
  fitness_value += (xi * xi) - (10 * cos(2 * PI * xi)) + 10;
  println("fitness_value =", fitness_value);

  return fitness_value;
}
```

```
int[] gwo_KPF(ArrayList<Wolf3>_population, double [] _signal, int
_max_iter, int _n, float _minx1, float _maxx1, float _minx2, float
_maxx2) {

  Random rnd = new Random(0);
  //int flag;

  Wolf3 alpha_wolf, beta_wolf, gamma_wolf;
  alpha_wolf = (Wolf3)_population.get(0);
  beta_wolf = (Wolf3)_population.get(1);
  gamma_wolf = (Wolf3)_population.get(2);

  int X1 = 0;
  int X2 = 0;
  int X3 = 0;
  int Xnew = 0;

  int [] best_position;

  println("K-position-pre[0] =",
((Wolf3)_population.get(0)).getPosition1());
  println("K-position-pre[1] =",
((Wolf3)_population.get(1)).getPosition1());
  println("K-position-pre[2] =",
((Wolf3)_population.get(2)).getPosition1());

  println("PF-position-pre[0] =",
((Wolf3)_population.get(0)).getPosition2());
  println("PF-position-pre[1] =",
((Wolf3)_population.get(1)).getPosition2());
  println("PF-position-pre[2] =",
((Wolf3)_population.get(2)).getPosition2());

  println("fitness-pre[0] =",
((Wolf3)_population.get(0)).getFitness());
  println("fitness-pre[1] =",
((Wolf3)_population.get(1)).getFitness());
  println("fitness-pre[2] =",
((Wolf3)_population.get(2)).getFitness());

  Collections.sort(_population, new WolfComparator3());

  // main loop of gwo
  int Iter = 0;
  while (Iter < _max_iter) {
    if (alpha_wolf.getPosition1() > 1) {

      // after every 10 iterations
      if (Iter % 10 == 0 && Iter > 0) {
        println("Iter = " + Iter + " best fitness = " +
alpha_wolf.getFitness());
      }
```

```
      // linearly decreased from 2 to 0
      float a = 2 * (1 - float(Iter) /_max_iter);

      // updating each population member with the help of best three
members
      for (int i = 0; i < _n; i++) {

        int Xnew_old = ((Wolf3)_population.get(i)).position1;
        int Ynew_old = ((Wolf3)_population.get(i)).position2;

        float A1, A2, A3;
        A1 = a * (2 * rnd.nextFloat() - 1);
        A2 = a * (2 * rnd.nextFloat() - 1);
        A3 = a * (2 * rnd.nextFloat() - 1);

        float B1, B2, B3;
        B1 = a * (2 * rnd.nextFloat() - 1);
        B2 = a * (2 * rnd.nextFloat() - 1);
        B3 = a * (2 * rnd.nextFloat() - 1);

        float C1, C2, C3;
        C1 = 2 * rnd.nextFloat();
        C2 = 2 * rnd.nextFloat();
        C3 = 2 * rnd.nextFloat();

        float D1, D2, D3;
        D1 = 2 * rnd.nextFloat();
        D2 = 2 * rnd.nextFloat();
        D3 = 2 * rnd.nextFloat();

        X1 = int(alpha_wolf.position1 - A1 * abs(C1 *
alpha_wolf.position1 - ((Wolf3)_population.get(0)).position1));
        X2 = int(beta_wolf.position1 - A2 * abs(C2 *
beta_wolf.position1 - ((Wolf3)_population.get(1)).position1));
        X3 = int(gamma_wolf.position1 - A3 * abs(C3 *
gamma_wolf.position1 - ((Wolf3)_population.get(2)).position1));
        Xnew += X1 + X2 + X3;

        Y1 = int(alpha_wolf.position2 - B1 * abs(D1 *
alpha_wolf.position2 - ((Wolf3)_population.get(0)).position2));
        Y2 = int(beta_wolf.position2 - B2 * abs(D2 *
beta_wolf.position2 - ((Wolf3)_population.get(1)).position2));
        Y3 = int(gamma_wolf.position2 - B3 * abs(D3 *
gamma_wolf.position2 - ((Wolf3)_population.get(2)).position2));
        Ynew += Y1 + Y2 + Y3;

        Xnew =int(Xnew/3.0);
        println("K-Xnew=", Xnew);

        Ynew =int(Ynew/3.0);
        println("PF-Ynew=", Ynew);

        if (Xnew>=_minx1 && Xnew<=_maxx1 && Ynew>=_minx2 &&
```

```
Ynew<=_maxx2) {
          // fitness calculation of new solution
          double fnew = vmd_gwo_3(Xnew, Ynew, _signal);
          // greedy selection
          if (fnew < ((Wolf3)_population.get(i)).getFitness()) {
            ((Wolf3)_population.get(i)).inputPosition1(Xnew);
            ((Wolf3)_population.get(i)).inputPosition2(Ynew);
            ((Wolf3)_population.get(i)).inputFitness(fnew);
          }
        }
      }
      Collections.sort(_population, new WolfComparator3());

      for (int i=0; i < _n; i++) {
        println("K-position-post[", i, "]=",
((Wolf3)_population.get(i)).getPosition1());
      }

      for (int i=0; i < _n; i++) {
        println("PF-position-post[", i, "]=",
((Wolf3)_population.get(i)).getPosition2());
      }

      for (int i=0; i < _n; i++) {
        println("fitness-post[", i, "]=",
((Wolf3)_population.get(i)).getFitness());
      }

      alpha_wolf = (Wolf3)_population.get(0);
      beta_wolf = (Wolf3)_population.get(1);
      gamma_wolf = (Wolf3)_population.get(2);
      Iter++;
      println("Iter=:", Iter);
    }
  }
  int optimized_K = alpha_wolf.getPosition1();
  int optimized_PF = alpha_wolf.getPosition2();

  println("optimized_alpha-K=:", alpha_wolf.getPosition1());
  println("optimized_alpha-PF=:", alpha_wolf.getPosition2());

  int[] position_2D = {optimized_K, optimized_PF};

  return position_2D;
}

double vmd_gwo_3(int _position1, int _position2, double[] _signal) {

  x_env_double = new double[_position1][T1];
  entropy = new float [_position1];
  entropy_double = new double [_position1];
  p = new double[T1];
  p_float = new float[T1];
```

```
  // alpha x10;
  Complex position_alpha = new Complex(_position2 * 10, 0);

  vmd_3 = new Vmd(_signal, position_alpha, tau, _position1, DC,
init, tol, N);
  u_t3 = vmd_3.vmd(_signal);

  for (int k = 0; k < T1; k++) {
    x_env_double[_position1-1][k] = 0.0D;
  }

  for (int j = 0; j<_position1; j++) {
    entropy[j] = 0.0;
  }

  for (int j = 0; j < _position1; j++) {

    hilb[j] = new Hilbert(u_t3[j]);
    hilb[j].hilbertTransform();
    x_env_double[j] = hilb[j].getAmplitudeEnvelope();

    for (int i=0; i < T1; i++) {
      p[i] = x_env_double[j][i]/array_sum_double(x_env_double[j]);
    }
    for (int i=0; i < T1; i++) {
      p_float[i] = (float)(p[i]);
    }
    entropy[j] = ent(p_float);
    entropy_double[j] = ent_double(p);
  }

  double entropy_double_all = array_sum_double(entropy_double)/
_position1;
  double entropy_min = min(entropy_double);
  double entropy_max = max(entropy_double);

  return entropy_max;
  //return entropy_double_all;
}

float array_sum_float(float[] _array) {
  float _array_sum = 0;

  for (int i=0; i<_array.length; i++) {
    _array_sum += _array[i];
  }
  return _array_sum;
}

float[] append_float(float [] array1, float [] array2) {
  float [] array;
  array = new float [array1.length+array2.length];
  for (int i = 0; i < array1.length; i++) {
    array[i] = array1[i];
```

```
  }
  for (int i = 0; i < array2.length; i++) {
    array[i+array1.length] = array2[i];
  }
  return(array);
}

void imfs_temp(double [][] _imfs) {
  for (int k=1; k < K+1; k++) {
    for (int i=0; i < int(T1); i++) {
      imfs[k-1][i] = _imfs[k-1][i];
      //u_n1_double[i] = u_double[k-1][i];
    }
  }
}

void imfs(double [][] _imfs) {
  for (int k=1; k < K+1; k++) {
    for (int i=0; i < int(T1); i++) {
      imfs_float[k-1][i] = (float) _imfs[k-1][i];
      //u_n1_double[i] = u_double[k-1][i];
    }
  }
}

float log2 (float x) {
  return (log(x) / log(2));
}

// wolf class
class Wolf3 {

  //int dim;
  float minx1;
  float maxx1;
  float minx2;
  float maxx2;
  int seed;

  int position1;
  int position2;

  //float fitness;
  double fitness;
  float rnd;

  //Constructor
  Wolf3 (double[] _signal, float _minx1, float _maxx1, float _minx2,
float _maxx2, int _seed) {
    //dim = _dim;
    signal = _signal;
    minx1 = _minx1;
    maxx1 = _maxx1;
    minx2 = _minx2;
```

```
    maxx2 = _maxx2;
    seed = _seed;
  }

  void init(double[] _signal, float _minx1, float _maxx1, float
_minx2, float _maxx2, int seed) {

    Random rnd;

    int position1;
    int position2;

    //float fitness;
    double fitness;
    this.position1 = 4;
    this.position2 = 2000;

    this.position1 = (int(random(minx1, maxx1)));
    this.position2 = (int(random(minx2, maxx2)));

    this.fitness = vmd_gwo_3(this.position1, this.position2,
signal);
    println("wolf no=: =", wolf_no);
    println("self.position1 =", this.position1);
    println("self.position2 =", this.position2);
    wolf_no += 1;
  }

  public double getFitness() {
    return this.fitness;
  }

  public int getPosition1() {
    return this.position1;
  }

  public int getPosition2() {
    return this.position2;
  }

  public void inputPosition1(int _position1) {
    this.position1 = _position1;
  }

  public void inputPosition2(int _position2) {
    this.position2 = _position2;
  }

  public void inputFitness(double _fitness) {
    this.fitness = _fitness;
  }
}

class WolfComparator3 implements Comparator<Wolf3> {
```

```
  @Override
    public int compare(Wolf3 p1, Wolf3 p2) {
    //if ((p1.getFitness() - p2.getFitness()) < .000000001)
    if ((p1.getFitness() - p2.getFitness()) == 0.0)
      return 0;
    if (p1.getFitness() > p2.getFitness())
      return 1;
    else if (p1.getFitness() < p2.getFitness())
      return -1;
    return 0;
  }
}

float ent(float[] data) {
  float ent = 0;
  for (int i=0; i< data.length; i++) {
    ent += data[i]*log2(data[i]);
  }
  return -abs(ent);
}

double ent_double(double[] data) {
  double ent = 0.0D;
  for (int i=0; i< data.length; i++) {
    ent += data[i]* (Math.log10(data[i])/Math.log10(2));
  }
  return -Math.abs(ent);
}

void get_eegData() {

  for (int j = 0; j < 64; j++) {
    for (int i = 0; i < 16; i++) {
      //x1[j*16+i] = (double) eeg_data.getFloat(rowCount-1-j, i+1);
      eeg_x1[j*16+i] = (double) eeg_data.getFloat(current_row + j +
1, i + 1);
      eeg_x1_Hz[j*16+i] = 0.125*(j*16+i);
    }
  }
}

double min(double[] values) {
  double min = values[0];
  for (double v : values) {
    if (v < min)
      min = v;
  }
  return min;
}

double max(double[] values) {
  double max = values[0];
  for (double v : values) {
```

```
    if (v > max)
      max = v;
  }
  return max;
}


//*****VMD.pde******
import com.github.psambit9791.jdsp.signal.Convolution;

import java.util.Arrays;
import java.util.Comparator;
import java.util.Random;

import com.github.psambit9791.jdsp.transform.Hilbert;

  // Constructor
  Vmd (double [] _signal, Complex _alpha, Complex _tau, int _K, int
_DC, int _init, double _tol, int _N) {
    signal2 = _signal;
    alpha = _alpha;
    tau = _tau;
    K = _K;
    DC = _DC;
    init = _init;
    tol = _tol;
    N = _N;
  }

  double[][] vmd(double [] _signal) {
    // ---------------------
    //  signal  - the time domain signal (1D) to be decomposed
    //  alpha   - the balancing parameter of the data-fidelity
constraint
    //  tau     - time-step of the dual ascent ( pick 0 for noise-
slack )
    //  K       - the number of modes to be recovered
    //  DC      - true if the first mode is put and kept at DC (0-
freq)
    //  init    - 0 = all omegas start at 0
    //                     1 = all omegas start uniformly
distributed
    //                     2 = all omegas initialized randomly
    //  tol     - tolerance of convergence criterion; typically
around 1e-6
    //
    //  Output:
    //  -------
    //  u       - the collection of decomposed modes
    //  u_hat   - spectra of the modes
    //  omega   - estimated mode center-frequencies
    //
    // Period and sampling frequency of input signal
    //-----define--------------
```

```
int save_T;
double fs;
float T_f;
int T2_f;
//Complex alpha= new Complex(2000.0, 0);
int N2;

//double uDiff = tol + 2.2204e-16; //updata step
double uDiff = tol + Math.ulp(1.0); //updata step

int n=1; //loop counter

Complex[] freqs_sub;
Complex[] freqs_square;
Complex[] freqs_square_sub;
Complex sum_uk[];
Complex Alpha[];

Complex[] f_hat;
Complex[] f_hat1;
Complex[] f_hat_plus;
Complex[] f_hat_plus_sub1;
Complex[] f_hat_plus_sub2;
Complex[] f_hat_plus_m1;
Complex[] f_hat_plus_m2;

Complex[][] omega;
Complex[][] omega_plus;
Complex omega_plus_n2;
Complex omega_plus_comp;

Complex[][] lamda_hat;
Complex[] lamda_hat_n1;
Complex[] lamda_hat_n2;

Complex[][] u_hat;
Complex[][] u_hat2;
Complex[][] u;
Complex[][] u_hat_n1;
Complex[] u_hat_n2;
Complex[] u_hat_n2_ishift;

Complex[][][] u_hat_plus;
Complex[] u_hat_plus_n1;
Complex[] u_hat_plus_n2;
Complex[] u_hat_plus_n3;
Complex[] u_hat_plus_n8;
Complex[] u_hat_plus_n9;
Complex[] u_hat_plus_n10;
Complex[] u_hat_plus_n11;
Complex[][] u_hat_plus_n17;
Complex[] u_hat_plus_n18;
Complex[][] u_hat_plus_n20;
```

```
Complex[] u_hat_plus_conj1;
Complex[] u_hat_plus_conj2;

Complex[] u_hat_plus_conj3;
Complex u_hat_plus_conj4;

Complex[] fft1_result;
Complex[][] fft2_result;
Complex[] fft3_result1;
Complex[] fft3_result2;
Complex[] fft3_result3;
Complex[] fft3_result3_t;

double f_mirror[];
double f[];
double freqs[];

double freqs_n1[];
double [][]omega_plus_double;
double[] u_hat_plus_n4;
double[] u_hat_plus_n5;
double u_hat_plus_n6;
double u_hat_plus_n7;
double[] u_hat_plus_n12;
double[] u_hat_plus_n13;
double[] u_hat_plus_n14;
double u_hat_plus_n15;
double u_hat_plus_n16;

double[][] _u_double;
double[][] _u_double1;
double[][] _u_double2;
double[] u_n1_double;

//-----memory keep---------
f_mirror = new double [2*T1];
//eeg = new double [2*T1];
f = new double [2*T1];

freqs = new double [2*T1];
Alpha = new Complex [K];
sum_uk = new Complex [2*T1];

u_hat = new Complex [2*T1][K];
u_hat2 = new Complex [2*T1][K];
u_hat_plus = new Complex [N][2*T1][K];
u_hat_plus_n1 = new Complex [2*T1];
u_hat_plus_n2 = new Complex [2*T1];
u_hat_plus_n3 = new Complex [2*T1];
u_hat_plus_n4 = new double [2*T1];
u_hat_plus_n5 = new double [2*T1];
u_hat_plus_n8 = new Complex [2*T1];
u_hat_plus_n9 = new Complex [2*T1];
u_hat_plus_n10 = new Complex [2*T1];
```

```
u_hat_plus_n11 = new Complex [2*T1];
u_hat_plus_n12 = new double [2*T1];
u_hat_plus_n13 = new double [2*T1];
u_hat_plus_n14 = new double [2*T1];
u_hat_plus_n17 = new Complex [2*T1][K];
u_hat_plus_n18 = new Complex [2*T1];
u_hat_plus_n20 = new Complex [T1][K];

f_hat = new Complex [2*T1];
f_hat1 = new Complex [freqs.length];
f_hat_plus = new Complex [2*T1];
f_hat_plus_sub1 = new Complex [2*T1];
f_hat_plus_sub2 = new Complex [2*T1];
f_hat_plus_m1 = new Complex [2*T1];
f_hat_plus_m2 = new Complex [2*T1];

u = new Complex [K][2*T1];

_u_double = new double [K][2*T1];
_u_double1 = new double [K][T1];
_u_double2 = new double [K][T1];
u_n1_double = new double [2*T1];
u_hat_n1 = new Complex [2*T1][K];
u_hat_n2 = new Complex [2*T1];
u_hat_n2_ishift = new Complex [2*T1];
u_hat_plus_conj1 = new Complex [2*T1];
u_hat_plus_conj2 = new Complex [2*T1];
u_hat_plus_conj3 = new Complex [2*T1];

omega = new Complex [N][K];
omega_plus = new Complex [N][K];
omega_plus_double = new double [N][K];

freqs_square_sub = new Complex [2*T1];
freqs_square = new Complex [2*T1];
freqs_sub = new Complex [2*T1];
freqs_n1 =new double [2*T1];

lamda_hat = new Complex [N][freqs.length];
lamda_hat_n1 = new Complex [freqs.length];
lamda_hat_n2 = new Complex [freqs.length];

fft1_result = new Complex [2*T1];
fft2_result = new Complex [K][2*T1];
fft3_result1 = new Complex [2*T1];
fft3_result2 = new Complex [2*T1];
fft3_result3 = new Complex [2*T1];
fft3_result3_t = new Complex [2*T1];

//---------body--------
signal2 = _signal;
save_T = _signal.length; //signal size 1024 to save_T
fs = 1/(save_T);  //Sampling interval fs is 1/1024
```

```
    // extend the signal by mirroring
    // Specifically, the first half of the signal is inverted
    // and added to the beginning of the original signal,
    // and the second half of the signal is inverted and added to
the tail of the original signal.
    // Then, signal size is 2048 data points.

    for (int i=0; i<int(T1/2); i++) {
      f_mirror[i] = signal2[int(T1/2)-1-i];
    }
    for (int i=int(T1/2); i<int(3*T1/2); i++) {
      f_mirror[i] = signal2[i-int(T1/2)];
    }
    for (int i=int(3*T1/2); i<int(4*T1/2); i++) {
      f_mirror[i] = signal2[int(T1-1)-(i-int(3*T1/2))];
    }

    //double eeg data: f = f_mirror;
    f = f_mirror;  //Store the mirrored EEG in a new variable f

    // Time Domain 0 to T (of mirrored signal)
    T_f = float(f.length); //Store 2048 mirror extension signal data
in T_f

    for (int i=0; i<T_f; i++) {
      t[i] = 1/T_f*(i+1);
    }

    // Spectral Domain discretization freq[]
    for (int i=0; i<T_f; i++) {
      freqs[i] = t[i] - 0.5 - 1/T_f;
    }

    // For future generalizations: individual alpha for each mode
    // penalty factor, balance parameter
    for (int i=0; i<K; i++) {
      Alpha[i] = alpha.multiply(ONE);
    }

    //int N = 500

    // Construct and center f_hat: Fourier transform of the signal
    FastFourierTransformer fft1 = new
FastFourierTransformer(DftNormalization.STANDARD);
    fft1_result= fft1.transform(f, TransformType.FORWARD);

    f_hat = fftshift(fft1_result); //Fourier transform result to
f_hat with zero frequency component shifted to center of spectrum

    f_hat_plus = f_hat;  //Copy f_hat to f_hat_plus

    for (int i=0; i<int(T_f)/2; i++) {
      f_hat_plus[i] = ZERO;  //Initialize 1-1024 data of f_hat_plus
with 0
```

```
    }

    // matrix keeping track of every iterant // could be discarded
for mem
    for (int i=0; i < N; i++) {
      for (int j=0; j < 2*T1; j++) {
        for (int k=0; k < K; k++) {
          u_hat_plus[i][j][k] = ZERO;  //Create a complex zero array
with u_hat_plus.shape:= (500, 2048, 3)
        }
      }
    }

    // Initialization of omega_k
    for (int j = 0; j < N; j++) {
      for (int k = 0; k < K; k++) {
        omega_plus[j][k] = ZERO; //Initialization of frequency
variable omega plus
      }
    }

    if (init == 1) {
      for (int i=1; i < K+1; i++) {
        Complex K_comp = new Complex(K, 0);
        Complex i_comp = new Complex(i, 0);
        omega_plus[0][i-1] =
(HALF.divide(K_comp)).multiply(i_comp.subtract(ONE));
      }
    } else if (init == 2) {
      for (int k = 0; k < K; k++) {
        Complex random_comp = new Complex(random(1), 0);
        Complex fs_comp = new Complex(fs, 0);
        omega_plus[0][k] =
((fs_comp.log()).exp()).add((HALF.log()).subtract(fs_comp.log()).mul
tiply(random_comp));   //missing sort()
      }
    } else {
      for (int k = 0; k < K; k++) {
        omega_plus[0][k] = ZERO;
      }
    }

    if (DC == 1) {
      omega_plus[0][0] = ZERO;
    }

    // start with empty dual variables  Lagrangian multiplier λ
    for (int i = 0; i < N; i++) {
      for (int j=0; j < freqs.length; j++) {
        lamda_hat[i][j] = ZERO;
      }
    }

    for (int i=0; i<freqs.length; i++) {
```

```
      sum_uk[i] = ZERO; //accumulator
    }
    T2_f = int(T_f);

    // ----------- Main loop for iterative updates
    // Update n to minimize u_hat and omega_hat
    // Algorithm 2 Complete optimization of VMD

    while (uDiff > tol && n < N) {
      //Repeat if tolerance of tol convergence criterion is greater
than Diff or less than N=2000.
      //if (uDiff > tol) {
      // update first mode accumulator
      int k = 1;

      for (int j=0; j < freqs.length; j++) {
        u_hat_plus_n1[j] = u_hat_plus[n-1][j][K-1];
        u_hat_plus_n2[j] = u_hat_plus[n-1][j][0];
        sum_uk[j] =
(u_hat_plus_n1[j].add(sum_uk[j])).subtract(u_hat_plus_n2[j]);
        //update spectrum of first mode through Wiener filter of
residuals
        //Wiener filter of residuals 論文の式(27)  u_hat_plus[][][]
        lamda_hat_n1[j] = lamda_hat[n-1][j];
        Complex freqs_comp = new Complex(freqs[j], 0.0);

        //Calculating freqs_square
        freqs_square_sub[j] = freqs_comp.subtract(omega_plus[n-1]
[k-1]);
        freqs_square[j] =
freqs_square_sub[j].multiply(freqs_square_sub[j]);

        //Calculation of u_hat_plus[][][]
        f_hat_plus_sub1[j]
=(f_hat_plus[j].subtract(sum_uk[j])).subtract(lamda_hat_n1[j].divide
(TWO));
        f_hat_plus_sub2[j] =
ONE.add(Alpha[k-1].multiply(freqs_square[j]));
        u_hat_plus[n][j]
[k-1]=f_hat_plus_sub1[j].divide(f_hat_plus_sub2[j]);
      }

      //update first omega if not held at 0
      //Equation (28) in the paper Substitutes the inner product for
the calculation of the integral.

      if (DC == 0) {
        //Computing omega_plus_double[][]
        //np.dot(freqs[T//2:T], (np.square(np.abs(u_hat_plus[n, T//
2:T, k-1]))).T)
        //Squared absolute value of u_hat_plus (n=0, last 1024 data,
k=0th) and inner product of frequency freqs[T//2:T]
        //np.sum(np.square(np.abs(u_hat_plus[n, T//2:T, k-1])))
        //Sum of squares of absolute values of u_hat_plus(n=0, last
```

```
1024 data, k=0th)

        for (int j=0; j < freqs.length/2; j++) {
          freqs_n1[j] = freqs[int(T2_f/2)+j];
          u_hat_plus_n3[j] = u_hat_plus[n][int(T2_f/2)+j][k-1];
          u_hat_plus_n4[j] =
((u_hat_plus_n3[j]).abs())*((u_hat_plus_n3[j]).abs());
        }

        u_hat_plus_n5 = array_transpose_double(u_hat_plus_n4);
        u_hat_plus_n6 = array_sum_double(u_hat_plus_n4);
        u_hat_plus_n7 = array_dot_double(freqs_n1, u_hat_plus_n5);
        omega_plus_double[n][k-1] = u_hat_plus_n7/u_hat_plus_n6;
      }

      for (k=2; k<K+1; k++) {
        //accumulator sum_uk[] calculation
        for (int j=0; j < freqs.length; j++) {
          u_hat_plus_n8[j] = u_hat_plus[n][j][k-2];
          u_hat_plus_n9[j] = u_hat_plus[n-1][j][k-1];

          sum_uk[j] =
(u_hat_plus_n8[j].add(sum_uk[j])).subtract(u_hat_plus_n9[j]);

          //mode spectrum //Update u^_k for all ω ≥0:
          lamda_hat_n2[j] = lamda_hat[n-1][j];
        }

        omega_plus_n2 = omega_plus[n-1][k-1];

        for (int i=0; i < freqs.length; i++) {
          //double freq =  freqs[i];
          Complex freqs_comp = new Complex(freqs[i], 0.0);
          freqs_sub[i] = freqs_comp.subtract(omega_plus_n2);
          freqs_square[i] = freqs_sub[i].multiply(freqs_sub[i]);
        }

        for (int i=0; i < freqs.length; i++) {
          f_hat1[i] = f_hat_plus[i].subtract(sum_uk[i]);
          f_hat_plus_m1[i] =
(f_hat1[i]).subtract(lamda_hat_n2[i].divide(TWO));

          f_hat_plus_m2[i] =
(ONE.add(Alpha[k-1].multiply(freqs_square[i])));
          //u_hat_plus k=0, 1, 2 renew
          u_hat_plus[n][i][k-1]=
f_hat_plus_m1[i].divide(f_hat_plus_m2[i]);
        }

        //center frequencies Update ω^_k:
        for (int j=0; j < freqs.length/2; j++) {
          freqs_n1[j] = freqs[int(T2_f/2)+j];
          u_hat_plus_n10[j] = u_hat_plus[n][int(T2_f/2)+j][k-1];
          u_hat_plus_n11[j] = u_hat_plus[n][int(T2_f/2)+j][k-1];
```

```
          u_hat_plus_n12[j]
=((u_hat_plus_n10[j]).abs())*(((u_hat_plus_n10[j]).abs()));
        }

        u_hat_plus_n13 = array_transpose_double(u_hat_plus_n12);

        for (int j=0; j < freqs.length/2; j++) {
          u_hat_plus_n14[j] =
((u_hat_plus_n11[j]).abs())*(((u_hat_plus_n11[j]).abs()));
        }

        u_hat_plus_n15 = array_dot_double(freqs_n1, u_hat_plus_n13);
        u_hat_plus_n16 = array_sum_double(u_hat_plus_n14);
        omega_plus_double[n][k-1]=u_hat_plus_n15/u_hat_plus_n16;
        omega_plus[n][k-1] = new Complex(omega_plus_double[n][k-1],
0);

        //Dual ascent
        //Thesis formula (29)

        for (int i=0; i < freqs.length; i++) {
          lamda_hat_n1[i] = lamda_hat[n-1][i];
        }

        for (int i=0; i < freqs.length; i++) {
          for (int j=0; j < K; j++) {
            u_hat_plus_n17[i][j] = u_hat_plus[n][i][j];
          }
          u_hat_plus_n18[i] = ZERO;

          for (int j=0; j < K; j++) {
            u_hat_plus_n18[i] =
(u_hat_plus_n18[i]).add(u_hat_plus_n17[i][j]);
          }
          lamda_hat[n]
[i]=lamda_hat_n1[i].add(tau.multiply(u_hat_plus_n18[i].subtract(f_ha
t_plus[i])));
        }
      }

      //loop counter Comparison with convergence criteria
      n = n + 1;
      //println("n=:", n);
      //converged yet?
      uDiff = 2.2204e-16;

      for (int i = 1; i < K+1; i++) {
        for (int j = 0; j < freqs.length; j++) {
          u_hat_plus_n1[j] = u_hat_plus[n-1][j][i-1];
          u_hat_plus_n2[j] = u_hat_plus[n-2][j][i-1];
          u_hat_plus_n3[j] =
u_hat_plus_n1[j].subtract(u_hat_plus_n2[j]);
          u_hat_plus_conj1[j] = u_hat_plus_n3[j].conjugate();
          u_hat_plus_conj2[j] = u_hat_plus_conj1[j].conjugate();
```

```
        }

        u_hat_plus_conj3 = array_transpose(u_hat_plus_conj2);
        u_hat_plus_conj4 = array_dot(u_hat_plus_n3,
u_hat_plus_conj3);
        uDiff = uDiff + 1/float(T2_f)* (float)
((u_hat_plus_conj4).abs());
      }
      uDiff = abs_double(uDiff);
    }
    //println("n=:", n);
    // ------ Postprocessing and cleanup
    // discard empty space if converged early

    N2 = min(N, n);

    for (int i = 0; i < N2; i++) {
      for (int j = 0; j < K; j++) {
        double omega_plus_temp = omega_plus_double[i][j];
        omega_plus_comp = new Complex(omega_plus_temp, 0.0);
        omega_plus[i][j] = omega_plus_comp;
        omega[i][j] = omega_plus[i][j];
      }
    }

    // Signal reconstruction: Calculate IMF from frequency
components by inverse Fourier transform
    for (int i=0; i<T2_f; i++) {
      for (int j=0; j<K; j++) {
        u_hat[i][j] = ZERO;
      }
    }

    for (int i=0; i<int(T2_f/2); i++) {
      for (int j=0; j<K; j++) {
        u_hat_plus_n20[i][j]  = u_hat_plus[N2-1][i+ int(T2_f/2)][j];
        u_hat[int(T2_f/2)+i][j]=(u_hat_plus_n20[i][j]);
        u_hat[int(T2_f/2)-i][j]=(u_hat_plus_n20[i][j]).conjugate();
        u_hat_n1[i][j] = u_hat[T2_f-1][j];
        u_hat[0][j]= (u_hat_n1[i][j]).conjugate();
      }
    }

    for (int j=0; j < K; j++) {
      for (int i=0; i< T2_f; i++) {
        u[j][i] = ZERO;
      }
    }

    for (int k=1; k < K+1; k++) {
      for (int i=0; i < T2_f; i++) {
        u_hat_n2[i] = u_hat[i][k-1];
      }
```

```
      u_hat_n2_ishift = ifftshift(u_hat_n2);  //reverse shift
      FastFourierTransformer fft2 = new
FastFourierTransformer(DftNormalization.STANDARD);
      fft2_result[k-1] = fft2.transform(u_hat_n2_ishift,
TransformType.INVERSE);  //inverse Fourier transform

      for (int i=0; i < T2_f; i++) {
        _u_double[k-1][i] = fft2_result[k-1][i].getReal();
        //_u_double[k-1][i] = fft2_result[k-1][i].abs();
      }
    }

    // remove mirror part
    for (int i=0; i<K; i++) {
      for (int j=int(T2_f/4); j<3*int(T2_f/4); j++) {
        _u_double1[i][j-int(T2_f/4)]=_u_double[i][j];
      }
    }

    //recompute spectrum: Obtain the frequency spectrum from the IMF
by Fourier transform.
    for (int i=0; i< int(T2_f); i++) {
      for (int j=0; j<K; j++) {
        u_hat[i][j] = ZERO;
      }
    }

    for (int k=1; k < K+1; k++) {
      for (int i=0; i < int(T2_f); i++) {
        //imfs_float[k-1][i] = (float) _u_double[k-1][i];
        u_n1_double[i] = _u_double[k-1][i];
      }

      FastFourierTransformer fft3 = new
FastFourierTransformer(DftNormalization.STANDARD);
      fft3_result1 = fft3.transform(u_n1_double,
TransformType.FORWARD);

      fft3_result2 = fftshift(fft3_result1);

      for (int i=0; i< int(T2_f/2); i++) {
        fft3_result3[i] = fft3_result2[i].conjugate();
      }

      fft3_result3_t =array_transpose(fft3_result3);

      for (int i=0; i < int(T2_f/2); i++) {
        u_hat2[i][k-1]= fft3_result3_t[i];
        _u_double2[k-1][i] = u_hat2[i][k-1].abs();
      }
    }
    return _u_double1;
  }
}
```

```
public static int[] argsort(final double[] a) {
  return argsort(a, true);
}

public static int[] argsort(double[] a, boolean ascending) {
  Integer[] indexes = new Integer[a.length];
  for (int i = 0; i < indexes.length; i++) {
    indexes[i] = i;
  }
  Arrays.sort(indexes, new Comparator<Integer>() {
    @Override
      public int compare(final Integer i1, final Integer i2) {
      return (ascending ? 1 : -1) * Double.compare(a[i1], a[i2]);
    }
  }
  );
  return asArray(indexes);
}

public static <T extends Number> int[] asArray(final T... a) {
  int[] b = new int[a.length];
  for (int i = 0; i < b.length; i++) {
    b[i] = a[i].intValue();
  }
  return b;
}

float[] array_square(float _array[]) {
  float _array2[];
  _array2 = new float [_array.length];

  for (int i=0; i<_array.length; i++) {
    _array2[i] = _array[i] * _array[i];
  }
  return _array2;
}

Complex[] fftshift(Complex _complex[]) {
  Complex complex2[];
  complex2 = new Complex [_complex.length];
  for (int i=0; i<_complex.length/2; i++) {
    complex2[_complex.length/2+i] = _complex[i];
  }
  for (int i=_complex.length/2; i<_complex.length; i++) {
    complex2[i-_complex.length/2] = _complex[i];
  }
  return complex2;
}

Complex[] ifftshift(Complex _complex[]) {
  Complex complex2[];
  complex2 = new Complex[_complex.length];
  for (int i=0; i<_complex.length/2; i++) {
```

```
    complex2[_complex.length/2+i] = _complex[i];
  }
  for (int i=_complex.length/2; i<_complex.length; i++) {
    complex2[i-_complex.length/2] = _complex[i];
  }
  return complex2;
}

double[] fftshift_d(double _double[]) {
  double double2[];
  double2 = new double[_double.length];
  for (int i=0; i<_double.length/2; i++) {
    double2[_double.length/2+i] = _double[i];
  }
  for (int i=_double.length/2; i<_double.length; i++) {
    double2[i-_double.length/2] = _double[i];
  }
  return double2;
}

double[] ifftshift_d(double _double[]) {
  double double2[];
  double2 = new double[_double.length];
  for (int i=0; i<_double.length/2; i++) {
    double2[_double.length/2+i] = _double[i];
  }
  for (int i=_double.length/2; i<_double.length; i++) {
    double2[i-_double.length/2] = _double[i];
  }
  return double2;
}

double[] append_double(double [] array1, double [] array2) {
  double [] array;
  array = new  double [array1.length+array2.length];
  for (int i = 0; i < array1.length; i++) {
    array[i] = array1[i];
  }
  for (int i = 0; i < array2.length; i++) {
    array[i+array1.length] = array2[i];
  }
  return(array);
}

Complex[] array_transpose(Complex _array[]) {
  Complex array2[];
  array2 = new Complex [_array.length];
  for (int j=0; j<_array.length; j++) {
    array2[j]=  _array[j];
  }
  return array2;
}

double[] array_transpose_double(double _array[]) {
```

```
    double array2[];
    array2 = new double[_array.length];
    for (int j=0; j<_array.length; j++) {
      array2[j]=  _array[j];
    }
    return array2;
  }

  Complex array_dot(Complex _array1[], Complex _array2[]) {
    Complex dot_result = new Complex(0, 0);
    for (int i=0; i<_array1.length; i++) {
      dot_result = dot_result.add(_array1[i].multiply(_array2[i]));
    }
    return dot_result;
  }

  double array_dot_double(double _array1[], double _array2[]) {
    double dot_result = 0;
    for (int i=0; i<_array1.length; i++) {
      dot_result += (_array1[i]*_array2[i]);
    }
    return dot_result;
  }

  Complex array_sum(Complex _array1[]) {
    Complex sum_result = new Complex(0, 0);
    for (int i=0; i<_array1.length; i++) {
      sum_result = sum_result.add(_array1[i]);
    }
    return sum_result;
  }

  double array_sum_double(double _array1[]) {
    double sum_result = 0;
    for (int i=0; i<_array1.length; i++) {
      sum_result += _array1[i];
    }
    return sum_result;
  }

  double abs_double(double _uDiff) {
    double _temp;
    if (_uDiff >= 0) {
      _temp = _uDiff;
    } else {
      _temp = - _uDiff;
    }
    return _temp;
  }
```
